# Pharmacological chaperone treatment with Cystadane in aspartylglucosaminuria: an open-label, phase Ib/II trial

**DOI:** 10.1038/s41467-026-78752-x

**Published:** 2026-11-11

**Authors:** Minna Laine, Antje Banning, Viljami Sairanen, Günter Lochnit, Timo Roine, Elina L. Harjunen, Ulrika Roine, Anna M. Tokola, Hanna Terinkoski, Daniel Leufkens, Päivi Helenius, Taina H. Autti, Ritva Tikkanen

**Affiliations:** 1 Department of Child Neurology, University of Helsinki and Helsinki University Hospital, Helsinki, Finland; 2 Institute of Biochemistry, Medical Faculty, University of Giessen, Giessen, Germany; 3 Baby Brain Activity Center, Children’s Hospital, Helsinki University Hospital and University of Helsinki, Helsinki, Finland; 4 Department of Radiology, Kanta-Häme Wellbeing Services County, Hämeenlinna, Finland; 5 Radiology, HUS Medical Imaging Center, University of Helsinki and Helsinki University Hospital, Helsinki, Finland; 6 Department of Neuroscience and Biomedical Engineering, Aalto University School of Science, Espoo, Finland; 7 School of Smart Solutions, Metropolia University of Applied Sciences, Vantaa, Finland; 8 Department of Phoniatrics, University of Helsinki and Helsinki University Hospital, Helsinki, Finland; 9 Institute of Medical Informatics, Medical Faculty, University of Giessen, Giessen, Germany

**Keywords:** Drug safety, Neurological disorders, Molecular medicine

## Abstract

This phase Ib/II investigator-initiated, open label trial aims to show the safety and efficacy of Cystadane (betaine) in aspartylglucosaminuria, a lysosomal storage disorder characterized by progressive cognitive decline, in Finnish patients homozygous for the Finnish major mutation, aged between 3 and 17 years at study begin (recruited: 25, analyzed: 21). Oral Cystadane efficacy and safety were studied over 48 months. The primary endpoint of the study, reduction of glycoasparagines in the urine, was quantitatively determined using liquid chromatography/mass spectrometry and normalized to urine creatinine. Urine glycoasparagines are significantly reduced upon Cystadane treatment (by 68.6% ±10.8%; *p* = 0.000), and the study thus meets its primary endpoint. Our data show that Cystadane/betaine efficiently improves the main disease biomarker, and it is safe to use in aspartylglucosaminuria. Further studies should be carried out in a larger patient population over a longer time to demonstrate the effects on disease progression. EudraCT registration number 2017-000645-48.

## Abstract (web summary)

Here, this phase Ib/II interventional clinical trial shows that betaine/Cystadane is safe to use and significantly reduces urinary accumulation of glycoasparagines and increases aspartylglucosaminidase activity in aspartylglucosaminuria.

## Introduction

Aspartylglucosaminuria (AGU, OMIM 208400) is a recessively inherited lysosomal disease characterized by a slowly progressive developmental delay that starts early in childhood and culminates in a severe intellectual disability at early adulthood^1,2^. Neuropsychological findings have revealed a profound cognitive dysfunction already by the age of 7 years, and there is very little cognitive development beyond this age^3^. In magnetic resonance imaging (MRI) studies, characteristic changes in some thalamic regions of AGU patients are present^4^, and accumulation of iron in the thalamus may contribute to the cognitive impairment in AGU^5^.

Pathogenic variants of the *AGA* gene are present in all patients, resulting in a deficiency of the lysosomal hydrolase aspartylglucosaminidase (N4-(β-N-acetylglucosaminyl)-L-asparaginase, EC 3.5.1.26, AGA). Most known AGU patients are of Finnish origin, and a great majority of them is homozygous for a specific AGA protein variant, p.Arg161Gln+Cys163Ser, also called AGU_Fin-major_. AGU patients from elsewhere in the world tend to exhibit their private gene variants, with few exceptions, and about 50 pathogenic variants are known in the literature^6–12^.

At the moment, there are no disease-specific treatments for AGU available, even though several options have been assessed in preclinical and clinical studies, including bone marrow and stem cell transplantation^13–19^, enzyme replacement therapy^20–24^, and gene therapy with retroviruses or adeno-associated viruses^23–25^. In addition, we have developed potential personalized treatments for specific variants, such as pharmacological chaperones (PC), translational read-through drugs, and splice-modulating substances^23–28^. We have shown that betaine functions as a PC for the AGU_Fin-major_ variant and increases the processing of the mutated precursor polypeptide into its active form^26^.

Betaine (tri-methyl glycine; CAS number 107-43-7) is a well-known, naturally occurring food ingredient^29^. In mammals, the two primary physiological roles of betaine are its function as a source of methyl groups and as an organic osmolyte^29^. Consistent with its function as a methyl group donor within the metabolism of methionine, betaine anhydrous has been approved for the treatment of homocystinuria under the trade names Cystadane® (https://www.ema.europa.eu/en/medicines/human/EPAR/cystadane) and Amversio® (https://www.ema.europa.eu/en/medicines/human/EPAR/amversio). Over the years, numerous homocystinuria patients have been treated with betaine, as it provides methyl groups for the re-methylation of homocysteine to methionine by the enzyme betaine-homocysteine-methyltransferase (BHMT), thereby reducing the levels of homocysteine^30,31^. Preclinical studies in peroxisomal Zellweger syndrome have also suggested that betaine may recover peroxisome function in patient cells with a specific missense variant^32,33^. Furthermore, in healthy subjects, betaine can help regulating serum homocysteine levels, and it is beneficial for the treatment of non-alcoholic fatty liver disease^34,35^.

We have previously shown in our in vitro experiments that betaine acts as a PC for some pathogenic missense variants of AGA, including AGU_Fin-major_, the worldwide most common AGU variant^26^. Consistent with its role as a PC, betaine can directly bind to and enhance the folding of the mutated AGA precursor, thus increasing its processing into the active heterotetrameric form. This results in an enhanced AGA activity in AGU patient fibroblasts^26^. As betaine is well tolerated upon oral use and has already been used in pediatric patients^36–39^, our previous in vitro findings encouraged us to perform a phase Ib/II clinical trial with betaine (Cystadane) in Finnish AGU patients homozygous for the AGU_Fin-major_ missense variant to study the safety and clinical efficacy of this drug in AGU.

Here, we show that betaine (Cystadane) is well tolerated in AGU and results in improvements in major disease biomarkers, including a reduction of urine glycoasparagines and an increase in AGA activity in patient blood. Furthermore, cognitive capabilities, assessed with standardized neuropsychological tests, show significant improvements in some subdomains. In magnetic resonance imaging-based analysis, reduction of iron accumulation in specific brain areas is observed. According to our data, betaine may provide an efficient and cost-effective oral treatment for AGU, also in pediatric patients.

## Results

### Phase Ib/II clinical trial design

**Fig. 1:**
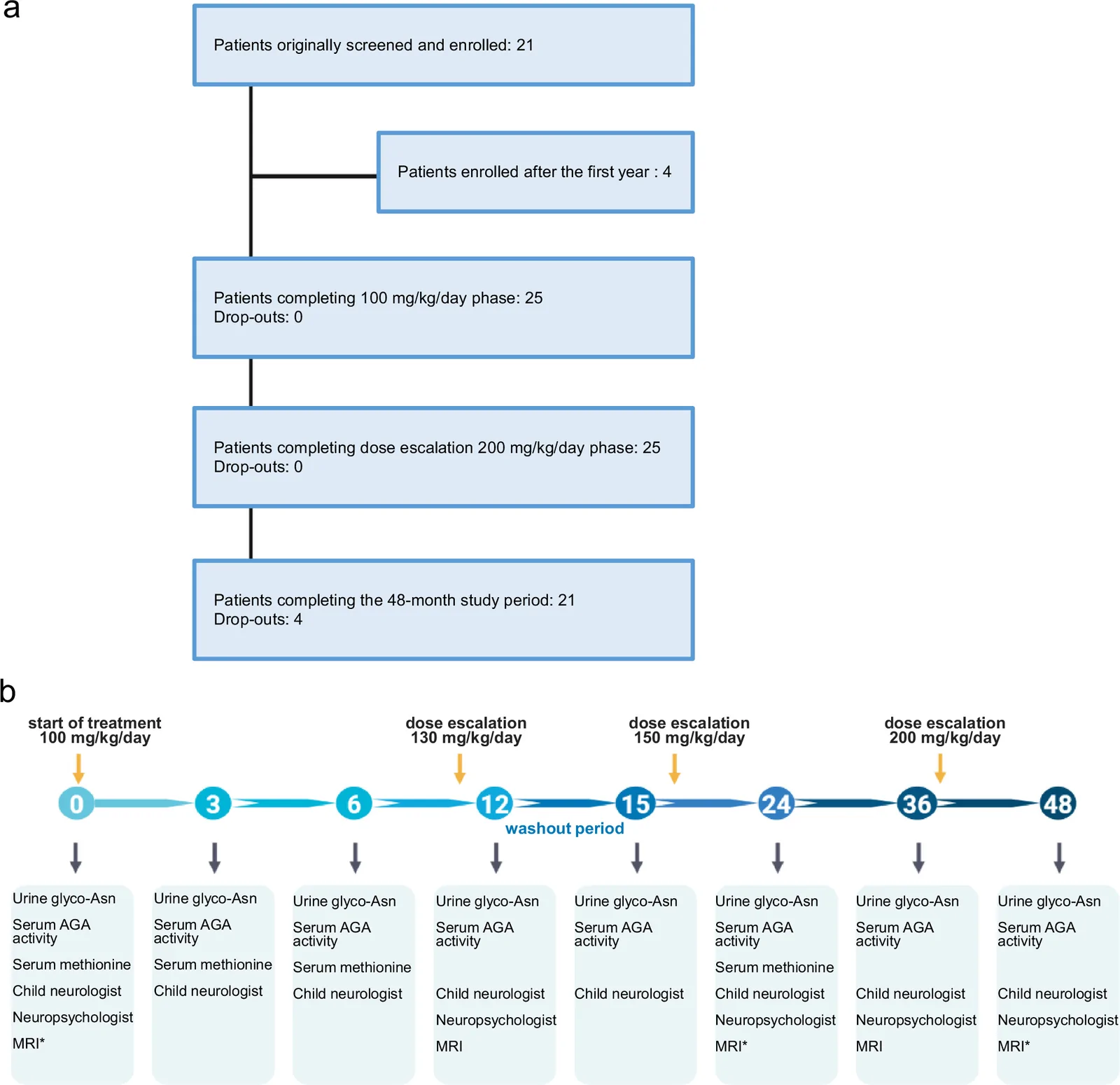
CONSORT diagram and the dose escalation and assessments carried out over the study time. **a** CONSORT diagram showing the study outline. **b** The daily Cystadane dose over the study months and the dose escalation strategy are depicted above the timeline. All patients started with 100 mg/kg/day, divided into two equal portions, and the dose was gradually escalated up to 200 mg/kg/day (or max 12 g/day), which was kept for the final 12 months. Time points of the clinical and biochemical assessments are shown in the boxes below the timeline. MRI* marks the time points of MR imaging for the patients requiring anesthesia. Created in BioRender. Tikkanen, R. (2026) https://BioRender.com/3kh1w5d.

The purpose of this investigator-initiated, single-center, open-label study (EudraCT registration number 2017-000645-48, see https://www.clinicaltrialsregister.eu/ctr-search/trial/2017-000645-48/FI, registration date May 31, 2017) was to explore the efficacy of oral Cystadane (betaine anhydrous) for the treatment of AGU, using a drug repurposing strategy. In addition, the safety and tolerability were assessed. An informed consent was signed by the parents of the participants before study begin. The study was initiated in February 2018 at the Helsinki University Hospital in Finland, after enrolling 21 participants between 7 and 15 years of age (11 females and 10 males). The baseline characteristics of the patients are depicted in Table 1, and a CONSORT diagram is shown in Fig. 1a. Within the original group, there were three sibling pairs. The 21 original study participants remained in the study for 48 months until study completion in April 2022. However, one participant declined the 48-month MRI, urine, and blood tests.

**Table 1:** Baseline characteristics of the study population

Original study population (*n* = 21)	
Age (years)	7.5–15.1
Sex	11 female, 10 male
Weight (kg)	29.0–102
GlcNAc-Asn (mg/g creatinine)	586–2070 (average 1225; reference value healthy individuals: 0)
AGA enzyme activity (mU/L)	0–0.238 (average 0.083; reference value healthy individuals: >2.0)
Serum methionine (µmol/L)	19–54 (average 31, reference value: <70)
WISC-IV (z scores)	−3 ≥ − 1.8 (average −2.7, reference value age-matched controls: 0)
Adaptive skills ABAS-3 (z scores)	−2.67 ≥ 0.53 (average −1.72, reference value age-matched controls: 0)

Patients enrolled at 15 months (*n* = 4)	
Age (years)	3.4–16.9
Sex	2 female, 2 male
Weight (kg)	15.0–92.0
GlcNAc-Asn (mg/g creatinine)	868–3146 (average 1801)
AGA enzyme activity (mU/L)	0.019–0.156 (average 0.062)

The data are divided into the original study population with 21 patients and the four patients enrolled at 15 months.

Further four patients (2 females, 2 males) were included in the study in May 2019, in accordance to our amended protocol. Three of these patients (numbers 22, 23, and 24) were between 3 and 6 years old, whereas patient 25 was 16 years and 11 months old. These four patients were included in the study at 15 months, where the original study patients finished the treatment break. Thus, patients 22–25 only participated in the study for a total of 33–34 months, as the study was closed when the original patients completed the 48-month study plan. In addition, two of these patients were too young (3 and 4 years) for the neuropsychological analyses at baseline. During MRI, these patients required anesthesia and were only imaged bi-annually, resulting in an incomplete data set (0, 24, and 33 months) that was not comparable with the original patient population. These four patients were thus considered as dropouts due to the missing data points at 36 and 48 months, and the data concerning these patients were excluded from the final analysis due to the incomplete data sets.

The age of the study participants at diagnosis varied between 2 and 8 years. The diagnosis of AGU had been carried out prior to the study begin by analysis of urine oligosaccharides (glycoasparagines) and confirmed by sequencing of the *AGA* gene. All enrolled patients are homozygous for the AGU_Fin-major_ variant. Further clinical details (e.g., prior and concomitant medication) of the study participants are depicted in Supplementary Information.

Oral Cystadane treatment was initiated after assessment by a child neurologist (time point 0 months). All patients immediately started Cystadane uptake with 100 mg/kg/day, divided into two equal portions. Since the recommendation for a maximum daily dose increased from 100 mg/kg/day (maximum of 6 g/day) to 200 mg/kg/day (maximum of 12 g/day) during the first year of the study, a dose escalation strategy was applied after approval of the protocol amendment by the local authorities. The dose was increased to 130 mg/kg/day for months 11 and 12 (Fig. 1b). All patients ceased Cystadane uptake between months 13 and 15 (a washout period of 3 months to replace a placebo group). After the break, the participants re-started with the same dose as before the washout period, and after one further month, the daily dose was gradually increased within 1 month, until reaching 150 mg/kg/day. The dose was further adjusted to 200 mg/kg/day within the 3rd year of the study for the 21 original participants (Fig. 1b), whereas the dose escalation was accelerated in the case of the four patients who joined the study later. As the pediatric patients also grew and gained weight over the study period, the absolute amounts of betaine were regularly adjusted according to the current weight.

The study participants underwent regular clinical assessment by a child neurologist at 0, 3, 6, 12, 15, 24, 36, and 48 months. Upon all visits, serum and urine samples were collected for biochemical analyses. Neuropsychological assessment and MRI were done annually, except when anesthesia was required for MRI. Figure 1b shows a timescale of the assessments that were carried out.

### Cystadane is safe and well tolerated in pediatric AGU patients

Cystadane was generally tolerated well, and no drug-related serious adverse events were observed over the study period (Table 2). Shortly after starting Cystadane intake, one family reported temporary gastrointestinal problems (diarrhea) that dissolved within a week. Since periodic diarrhea is very common in AGU patients, it is unclear if these symptoms were related to the medication, and they were recorded as possibly related. Parents of three participants reported that an unpleasant odor of the body and the breath of the patients appeared after some weeks of Cystadane treatment or upon dose escalation, and it was either temporary or persisted over the study period. Some of the adverse events were judged as not related, as they were already present before the study begin. Urine incontinence increased in two patients, and one patient experienced a single epileptic seizure unrelated to the medication. One patient with a pre-existing cardiac valve abnormality had bacteremia due to an accident-related dental problem during the first year of the study, but this was considered as moderate and unrelated to the study drug. The parents only reported this incidence during the next visit to the child neurologist. Two participants had a mild, transient elevation of liver transaminases, and one patient each suffered from insomnia and mood disorder that were considered unrelated to the study substance.

**Table 2:** Adverse events observed over the study course, classified as mild, moderate, or severe

Adverse events	Patients *n* = 25	Severity
**Total overall**	**13**	**Mild, moderate**
**Related overall** **General** -body odor	**3** **3** 3	**Mild** **Mild** Mild
**Possibly related overall** **GI symptoms** -gastroenteritis ac.	**1** 1 1	**Mild** Mild Mild
**Not related/pre-existing overall** **Nervous system** -epileptic seizure -headache **Infection** -bacteremia after tooth trauma **Urinary tract disorder** -incontinence **Liver** -transaminase elevation **Psychiatric disorder** -insomnia -mood disorder	**9** **2** 1 1 **1** 1 **2** 2 **2** 2 **2** 1 1	**Mild, moderate** **Mild, moderate** Moderate Mild **Moderate** Moderate **Mild, moderate** Mild, moderate **Mild** Mild **Moderate** Moderate Moderate

Upon visits to the child neurologists, the parents frequently reported improved sleep and behavioral changes, including reduced aggression and easier communication, after only a few weeks of starting the Cystadane medication. During the first year of betaine treatment, concentration, speech and motor skills of the patients seemingly improved, as subjectively reported by the parents. Only for two of the original 21 patients and one patient joining the study at 15 months, no subjective benefits were reported.

Since Cystadane can cause elevated plasma methionine levels in homocystinuria patients with specific gene variants, plasma methionine levels were determined as a safety measurement (at 0, 3, 6, and 24 months). The levels showed minor increases during Cystadane use, but they generally remained within the reference range (below 70 µmol/L for children ≤16 years). Only one participant, patient 16, showed an increase in plasma Met after 24 months of Cystadane use, but also in this case, the values remained below 100 µmol/L (Supplementary Table 1), and far below the safety limit of 700 µmol/L.

### Efficacy outcomes

#### Urine glycoasparagines are significantly reduced by Cystadane

**Fig. 2:**
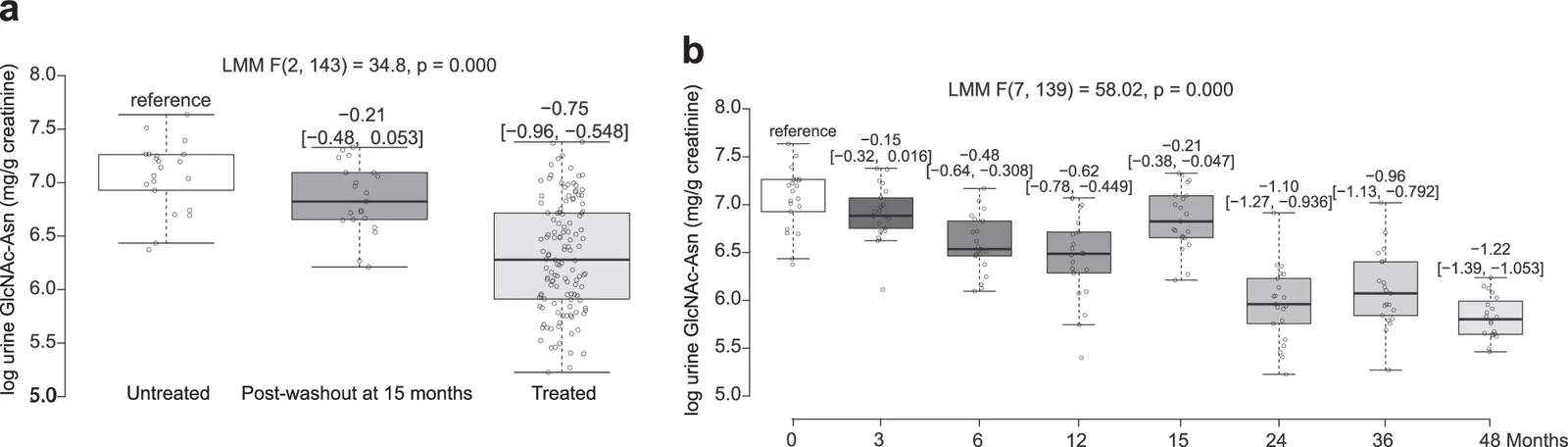
Decrease in urine GlcNAc-Asn during Cystadane treatment. GlcNAc-Asn and creatinine were measured in morning urine at the indicated time points of the study. All samples were measured simultaneously with technical triplicates (number of patients: 21 for other time points, 20 for 48 months). **a** The baseline values (untreated, *n* = 21), post-washout at 15 months (*n* = 21), and the combined treated time points were compared (*n* = 125). **b** Comparison of individual time points (untreated, post-washout, and treated time points 3, 6, 12, 24, 36 months: *n* = 21, 48 months *n* = 20). **a**, **b** The available data points were converted to degrees of freedom, and a statistical analysis was performed to investigate whether betaine treatment had a significant effect on GlcNAc-Asn excretion. Due to violation of the normal distribution assumption, the data were ln-transformed. The graphs show all individual data points and the aggregated trends. Box plots show the median (line within the box) and the 25th–75th percentiles (interquartile box). The whiskers cover 1.5 times the interquartile range. To account for the repeated measures design, a linear mixed model (LMM) was fitted to the data. The LMM was evaluated using a standard ANOVA approach with an F test (F), resulting in two-sided *p*-values. No adjustments for multiple hypothesis testing were performed. Comparison values shown on top of each whisker depict effect size differences to the reference [95%-CI], as estimated by the LLM. Source data are provided as a Source Data file.

The primary endpoint of our study for the clinical efficacy of Cystadane in AGU was the reduction of the excretion of glycoasparagines in the urine. The presence of glycoasparagines in the urine is a major diagnostic biomarker for AGU, and healthy individuals or AGU carriers do not have any measurable amounts of glycoasparagines in the urine. The main accumulation product in AGU, N-acetylglucosamine-asparagine (GlcNAc-Asn), was quantitatively assessed by liquid chromatography-mass spectrometry (LC-MS; raw data provided in Supplementary Data 1 file). Creatinine values in the urine samples were measured; the urine GlcNAc-Asn values were normalized to creatinine and expressed as ln-transformed values for mg GlcNAc-Asn per gram creatinine (Fig. 2a, b, individual values in Supplementary Fig. 1).

Urinary excretion of GlcNAc-Asn was significantly reduced already after 6 months of treatment, and the 3-month washout period (months 13–15) resulted in an increase of urine GlcNAc-Asn, demonstrating that the observed reduction was due to Cystadane/betaine (Fig. 2a, b). Dose escalation after the washout period (Figs. 1 and 2b) further reduced the excretion of GlcNAc-Asn in the urine to 31.4% (±10.8%, min 17.7%, max 54.2%; *p* = 0.000). of the untreated levels at 48 months, demonstrating that Cystadane treatment efficiently and significantly reduced urine GlcNAc-Asn, the major diagnostic biomarker for AGU.

#### AGA activity in serum is significantly increased by Cystadane

**Fig. 3:**
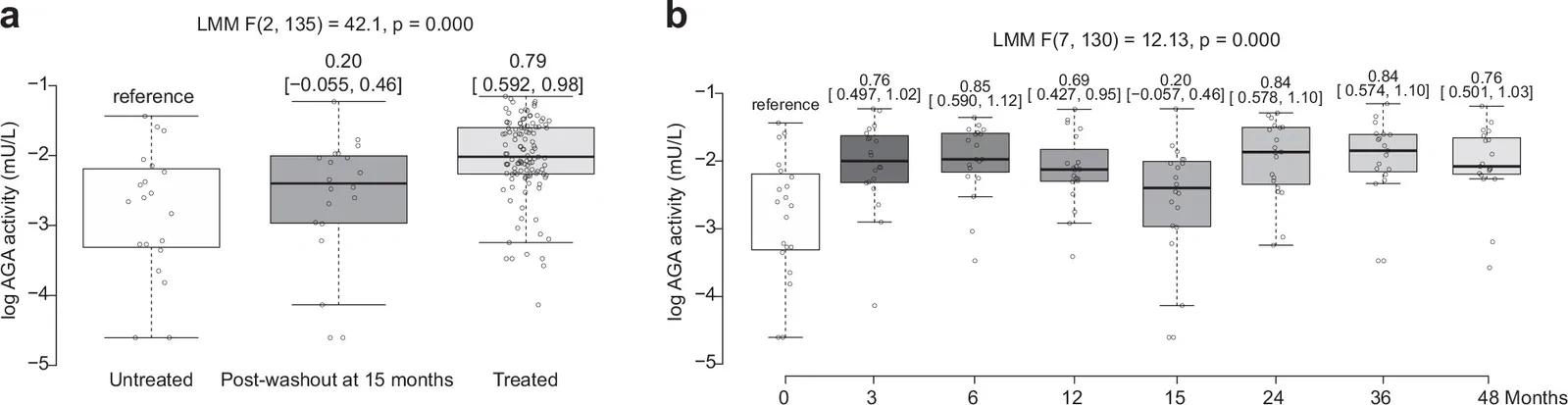
Increased serum AGA enzyme activity over the study course. Serum was collected at the beginning of the study and at the indicated time points. AGA activity was measured simultaneously in all samples with technical triplicates (number of patients: 21 for other time points, 20 for 48 months). A statistical analysis with all available data points was performed as described in Fig. 2. **a** AGA enzyme activity at baseline (0 months, *n* = 20), post-washout at 15 months (*n* = 20), and the combined treated time points (*n* = 117) are shown. **b** Comparison of individual time points (0, 3, 12, 15, and 24 months: *n* = 20 each; 6, 36, and 48 months: *n* = 19 each). **a**, **b** The graphs show all individual data points and the aggregated trends. Box plots show the median (line within the box) and the 25th–75th percentiles (interquartile box). The whiskers cover 1.5 times the interquartile range. To account for the repeated measures design, a linear mixed model (LMM) was fitted to the data. The LMM was evaluated using a standard ANOVA approach with an F test (F), resulting in two-sided *p*-values. No adjustments for multiple hypothesis testing were performed. Comparison values shown on top of each whisker depict effect size differences to the reference [95%-CI], as estimated by the LMM. Source data are provided as a Source Data file.

As a secondary endpoint, the AGA enzyme activity was quantitatively assessed in the serum samples and expressed as mU/L serum. The AGA activity measurement was carried out according to our validated protocol for human serum^40^. Healthy persons exhibit serum AGA activities within the range 2.503–3.897  mU/L^40^. At study begin, there was a large variation in the absolute AGA enzyme activities among the patients, with some patients showing baseline values too low to be measured reliably (Fig. 3a, b, individual data in Supplementary Fig. 2). Since hemolysis inhibits the AGA activity measurement^40–43^, clearly red serum samples (all samples of patient 21, and two further samples: patient 3: 6 months and patient 9: 36 months) were excluded from the data analysis. Patient number 19 refused to give a blood sample at 48 months. Nevertheless, there was a significant increase in the serum AGA activity already after 3 months of Cystadane treatment, whereas the activity dropped close to the untreated level during the washout period (3 months between months 13 and 15). The AGA activity increased again by 1.72 fold after restarting Cystadane intake with dose escalation (Fig. 3b). These data show that Cystadane treatment results in a moderate but significant increase in AGA activity in the patient sera, consistent with the suggested role of betaine as a pharmacological chaperone for AGA^26^.

#### Neuropsychological tests reveal a heterogeneous development

Cognitive development of the participants was assessed annually with Wechsler’s Intelligence Scale for Children IV (WISC-IV), which contains four subscales: verbal comprehension index (VCI), perceptual reasoning index (PRI), working memory index (WMI), and processing speed index (PSI, version B)^44,45^. Since AGU patients exhibit a severe developmental delay^3^, the raw scores of the patients in VCI, PRI, and WMI subscales were compared with the pre-existing data from a standardized Finnish population norm sample of 6-year-old children. For the PSI version B, no reference sample of 6-year-old children exists, as this version is used for children at the age of eight and above. Thus, the reference sample for 8-year old children was used for this subscale. A more detailed analysis of the data of the participants at study begin (0 months) is provided in Harjunen et al.^3^, showing that there is no age-dependent increase of the raw scores among AGU patients.

**Fig. 4:**
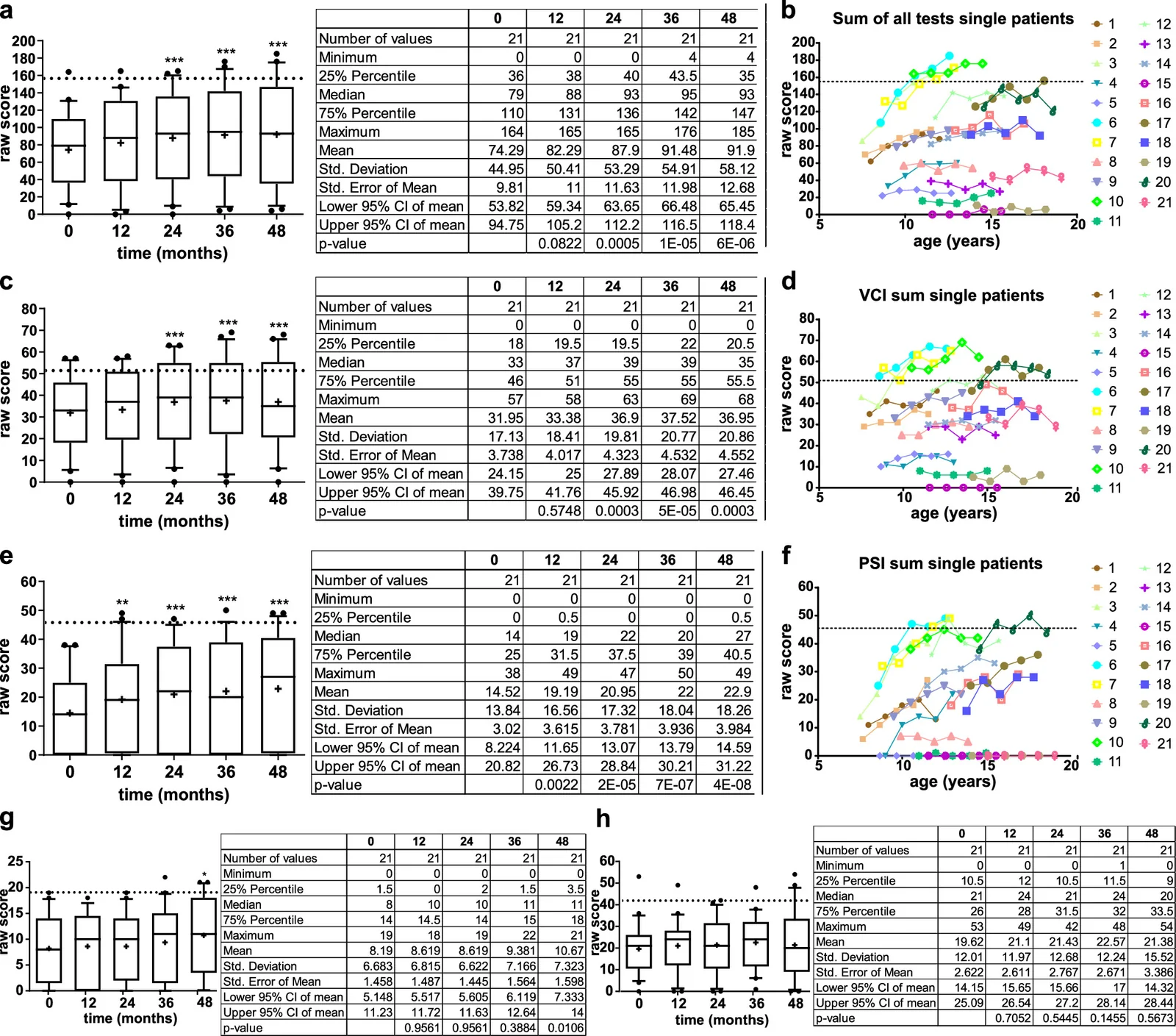
Neuropsychological data: sum of all subscales. All patients were assessed with the Wechsler Intelligence Scale for Children-IV (WISC-IV). Raw scores of all four subscales, i.e., VCI (verbal comprehension), PRI (perceptual reasoning), WMI (working memory), and PSI (processing speed) were added as the total raw score. **a** Boxplots present the median and 25th–75th percentiles; + indicates the mean value. Whiskers show the 10th–90th percentile. Data points outside of the 10th or 90th percentile are shown as dots. ***p < 0.001 vs. 0 months, 1-way ANOVA with Dunnett’s multiple comparisons test. **b** The data from (**a**) are shown for individual patients in relation to the patient age. The dotted lines represent the average raw score of a healthy 6-year old Finnish reference cohort. Raw scores of the indices VCI (**c**, **d**), PSI (**e**, **f**), WMI (**g**), and PRI (**h**), with individual data for VCI (**d**) and for PSI (**f**). Boxplots present the median and 25th–75th percentile; + indicates the mean value. Whiskers show the 10th–90th percentile. Data points outside of the 10th or 90th percentile are shown as dots. *n* = 21. ***p < 0.001, **<0.01, *<0.05 vs. 0 months, 1-way ANOVA with Dunnett’s multiple comparisons test. The exact two-sided *p*-values are shown on the right of the graphs. The dotted lines represent the mean of the 6-year-old (VCI, PRI, and WMI) or 8-year-old (PSI) Finnish reference population. Each patient is represented with an individual color and symbol in (**b**, **d**, **f**). Source data are provided as a Source Data file.

Figure 4a, b show the analysis of the sum of raw scores of the AGU patients within all four WISC-IV subscales over the study time (0, 12, 24, 36, and 48 months). The z scores of the raw score sums at 0 months suggested substantial deficits in the cognitive development of the study participants (average −2.7; range from −3 to −1.8; Table 1). There is a large variation in the raw score sums of the patients at all time points, but there is a significant increase in the raw score sums at 24, 36, and 48 months (Fig. 4a, b), also when analyzed using the LMM transformation model (Supplementary Fig. 3a). Two patients were incapable of performing the tests at 0, 12, and 24 months, and scored very low at later time points (Fig. 4b). However, several patients showed a positive development of the raw score sums over time (Fig. 4b).

Over the study period, there was a significant increase in the raw scores for VCI and PSI subscales within the patient cohort (Fig. 4c–f), whereas the WMI and PRI subscales showed no substantial increase, except for WMI at 48 months (Fig. 4g, h, and Supplementary Fig. 3b, c). At study begin, 14% of the participants reached the level of the 6-year old norm sample (51 points) for the VCI subscale, whereas 29% of them reached this level at 48 months, indicating a positive development (Fig. 4c, d). The VCI subscale consists of three subtests: comprehension, similarities, and vocabulary. Especially the VCI comprehension subtest showed a significant increase beyond 24 months, with 38% of the patients reaching the 6-year-old level (20.5 points) (Supplementary Fig. 4). For the PSI subscale, there was a significant positive development of the raw scores from 12 months onwards (Fig. 4e, f), and the increase in the PSI raw score sums was age independent (Fig. 4f). As the PSI reference norm group, 8-year-old children were used. Of the two subtests of PSI, especially the coding subtest showed significant improvements of the raw scores (Supplementary Fig. 4).

#### Positive development of adaptive skills

**Fig. 5:**
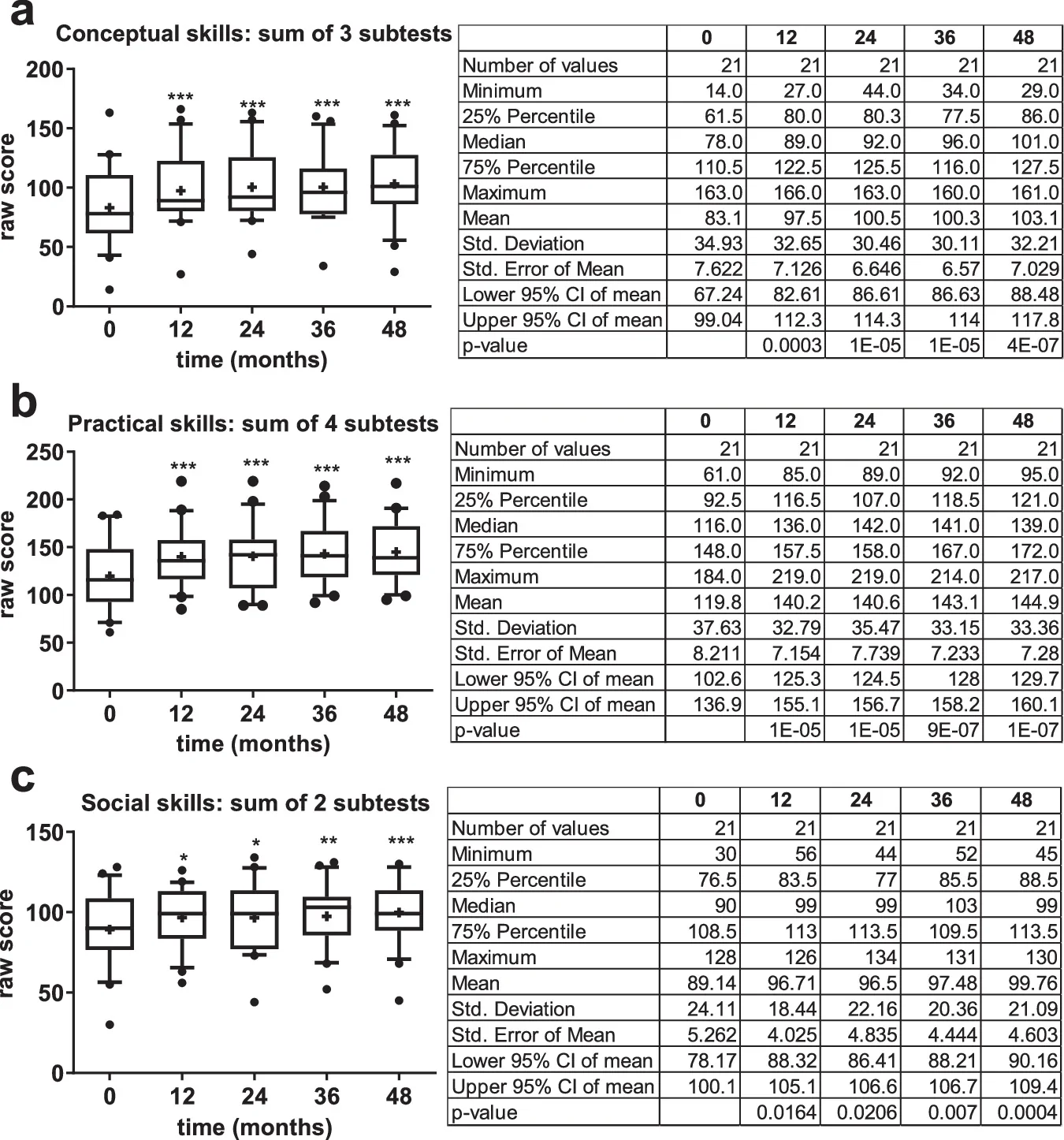
Adaptive skills: ABAS-3 subdomains. The parents annually completed the Finnish version of the ABAS-3 questionnaire during their visit at the neuropsychologist. The raw score sums for the three main domains **a** conceptual skills, **b** practical skills, and **c** social skills are shown. Boxplots present the median and 25th–75th percentiles, + indicates the mean value. Whiskers show the 10th–90th percentiles. Data points outside of the 10th or 90th percentile are shown as dots. Data are shown for 21 patients; *n* = 21 for all time points. ***p < 0.001, **p < 0.01, *p < 0.05, 1-way ANOVA with Dunnett’s multiple comparisons test vs. 0 months. The exact two-sided *p*-values are shown on the right of the graphs. Source data are provided as a Source Data file.

The Adaptive Behavior Assessment System, version 3 (ABAS-3) was used to assess the daily living skills of the participants^46^. At study begin, average z scores of −1.72 (range from −2.67 to 0.53; Table 1) for the participants revealed a high degree of functional impairment. The parents annually filled out this behavioral rating scale, and the raw scores of the questionnaire were assessed. ABAS-3 contains three subdomains (conceptual skills, practical skills, and social skills) that are further divided into subtests. All three subdomains showed a significantly increased sum of the raw scores over the study course (Fig. 5). The development of the raw scores for individual patients is depicted in Supplementary Fig. 5. All subtests of the three subdomains of ABAS-3 also showed a significantly positive development that was already visible after 1 year of treatment (Supplementary Fig. 6).

#### Quality of life questionnaires

The Kid- and Kiddo-KINDL questionnaire (parent version) was used to assess the quality of life in AGU patients. The test consists of six dimensions: physical well-being, emotional well-being, self-esteem, family, friends, and school^47–51^. For our analysis, only the results of the parent versions were used, since AGU patients are unable to complete the questionnaire. The total scores of the patients did not show a statistically significant difference between 0 and 48 months, and no significant differences were found in any of the six subscales (Supplementary Fig. 7 and Supplementary Table 2). Due to the small group size, it was not feasible to interpret the results for different age groups. As a reference group, a German cohort was used, as there are no standard values for children and adolescents from Finland. Nevertheless, in some of the subscales (i.e., physical well-being, self-esteem, family, school), AGU patients achieved similarly high scores as the healthy reference group (Supplementary Fig. 7).

#### Assessment of motor skills as an exploratory analysis

**Fig. 6:**
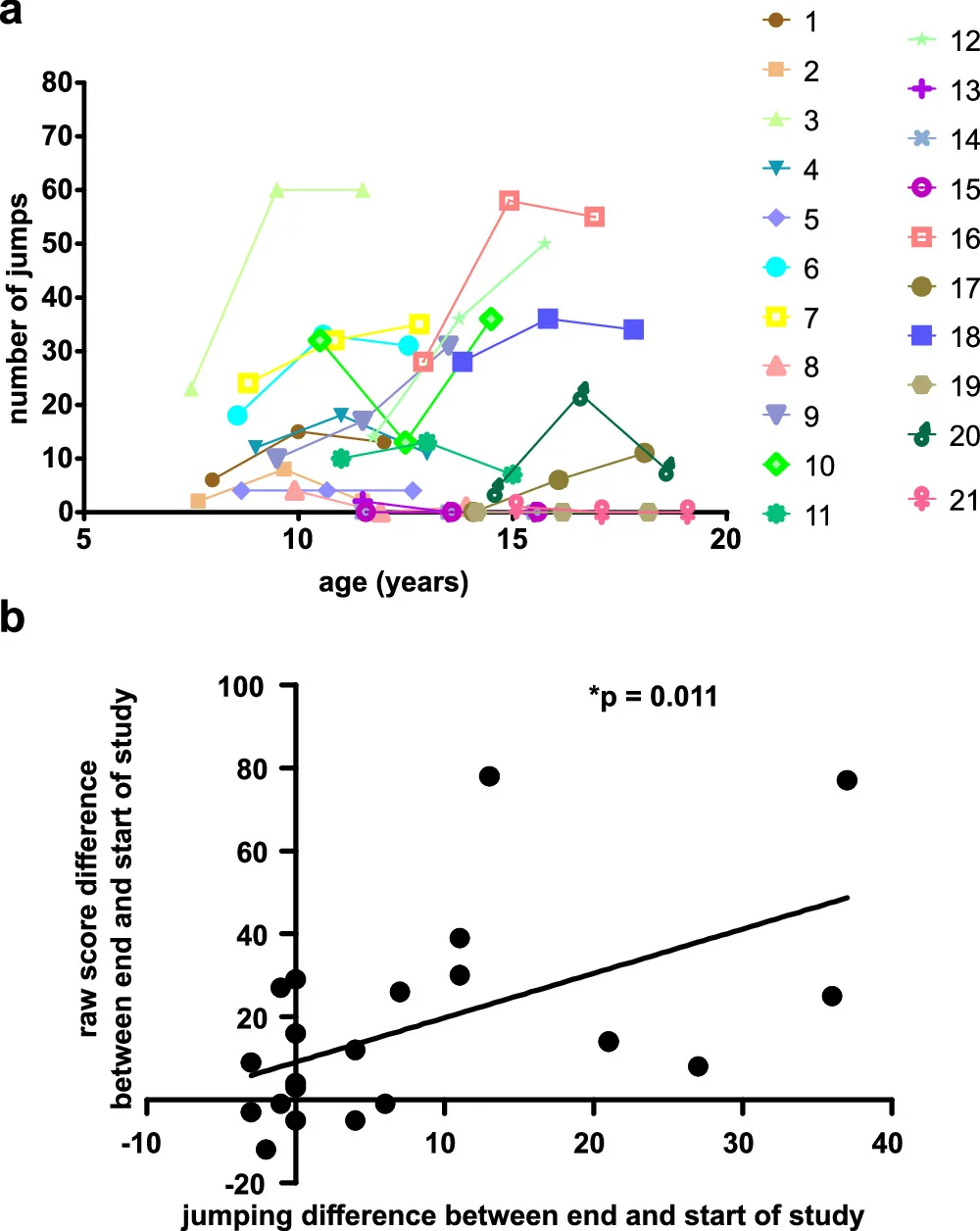
Jumping scores of individual patients, all time points. All patients performed a coordination test and were asked to jump on one leg as many times as possible. The maximum score was reached at 30 times per each leg, a total of 60 times. **a** Individual jumping scores in relation to the patient’s age. Each patient is represented with an individual color and symbol. **b** The results of the WISC-IV and the jumping scores at 48 months were compared with the results at study begin, and the difference was calculated. The raw score difference of WISC-IV was correlated with the difference in the jumping test. *p = 0.0105, two-tailed Pearson correlation, coefficent *r* = 0.5459; 95% confidence interval: 0.1493–0.7912; *R*^2^ = 0.2980. Source data are provided as a Source Data file.

During the visit to the child neurologist, motor skills were assessed as an exploratory outcome (not pre-defined in our study plan) at 0, 24, and 48 months with the task of jumping on one leg. The maximum score was reached upon jumping 30 times/leg (sum of both legs: 60, whereas higher scores were not assessed). The individual patient data are depicted as colored lines in Fig. 6a, and the correlation of the changes in the jumping scores with the raw score changes in the neuropsychological tests (sum of WISC-IV subscales) between baseline and 48 months are shown in Fig. 6b. The motor skills of the AGU patients were heterogeneous at baseline (0 months, Fig. 6a), but they positively correlated with the raw score gains in WISC-IV tests (Fig. 6b). A delay in motor development was present already in many of the youngest AGU patients at this time point, with most patients remaining below the expectation score set by the examiner (total of 10 times, 5 times/leg). Upon completion of the Cystadane treatment (48 months), the scores of especially those patients that were above the expectation score at study begin showed an improvement (Fig. 6b). As overweight is common among AGU patients, especially above the age of 10, it may have an impact on jumping skills. However, as with the neuropsychological findings, there were patients with especially low performance in the jumping scores, which was not dependent on age (Fig. 6a). Some patients (numbers 15 and 19) were unable to perform the requested tasks at any time point.

#### MRI findings

MRI was performed under anesthesia in some patients due to their young age and/or restlessness. For such patients, MRI was only performed bi-annually (0, 24, and 48 months) due to safety issues related to anesthesia, whereas all other patients were imaged at 0, 12, 24, 36, and 48 months. Twelve of the original 21 AGU patients were sedated for the first MR imaging examination, whereas nine patients were imaged without sedation. Eight of the non-sedated patients had motion artifacts in some sequences, and 10 patients were sedated for the last examination. One patient refused the imaging at 48 months. All four participants that joined the study at 15 months were sedated for the three MRIs performed.

**Fig. 7:**
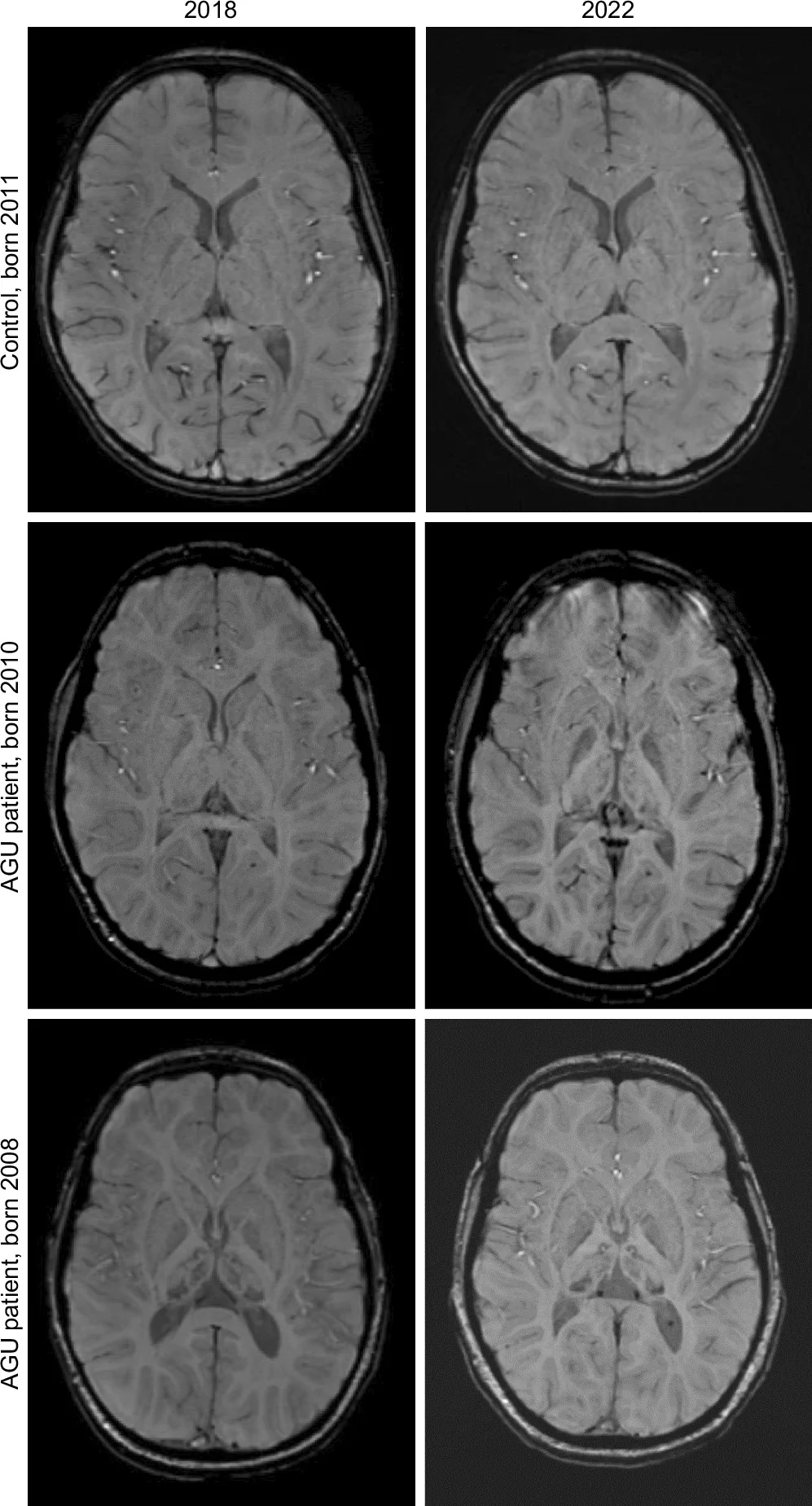
Representative MRI images (SWI sequence) of one control and two AGU patients. Susceptibility-weighted axial images to study begin (2018) and at 48 months (2022) at the level of the basal ganglia and thalami are shown. Uppermost row: a healthy control (born 2011), with normal findings without hypo-intensities in the thalami. Middle row: AGU patient (born 2010) showing a hypo-intensity of the thalami, with a maximum intensity decrease in the pulvinar nuclei. Bottom row: AGU patient (born 2008), whose thalami show overall abnormal signal intensity, especially in the pulvinar regions.

In our original cohort of 21 AGU patients, brain alterations that are typical for AGU were present in all patients at study begin (0 months)^4^. Figure 7 shows representative images of one control and two patients at the beginning and the end of the study. The observed alterations included signal intensity decrease in the thalami, especially in the pulvinar nuclei, periventricular signal intensity increase, and juxta-cortical high-signal foci. In addition, poor differentiation between gray and white matter was observed in all patients, particularly in the parietal and frontal regions, most likely representing delayed myelination. A thin corpus callosum, mildly dilated ventricles or cortical sulci, and cerebellar atrophy were also common findings in the patient group, as described previously^4^. The last MRIs at 48 months did not show any significant changes, neither improvement nor worsening, in visual analysis when compared to the MRIs at study begin. However, in three patients, the myelination had progressed in some brain areas.

#### Iron accumulation in the thalamus as exploratory analysis

**Fig. 8:**
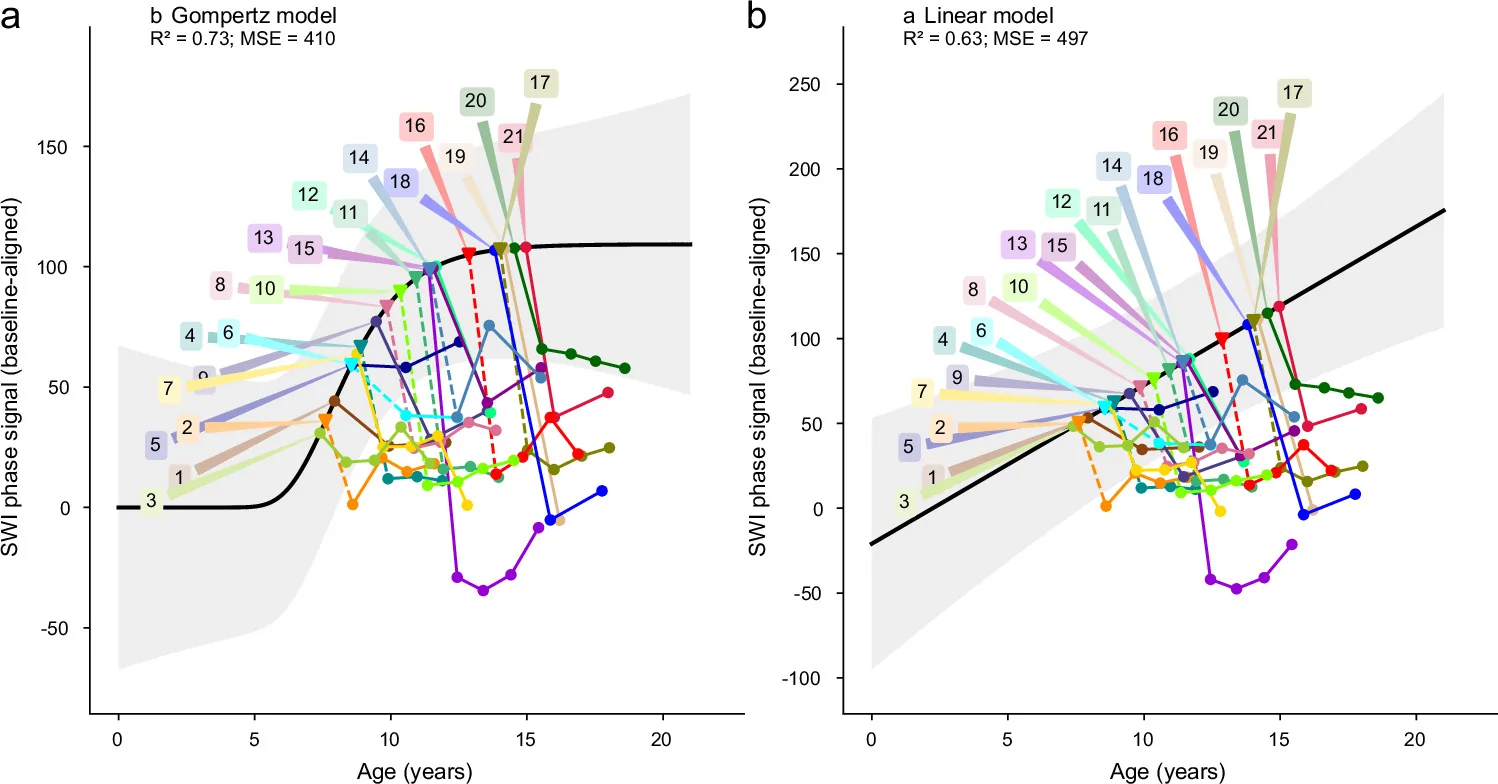
Iron accumulation is reduced in the thalamic regions of AGU patients. Two prediction models were constructed from the thalamic iron accumulation data derived from the SW-based MR images of the study participants at the beginning of the study. According to the Gompertz model (**a**), a plateau is reached around 15 years of age, whereas the linear model (**b**) predicts a continuous increase of iron. The linear model and the Gompertz model were fitted to the extracted phase-signals of susceptibility-weighted images to obtain 95% prediction intervals, coefficient of determination (*R*^2^), and mean-squared error (MSE). For details of the statistical analysis, please refer to Methods. The solid black line shows the predicted SWI phase signal for the respective age; the gray area depicts the 95% prediction interval. Each color represents the data of one patient. The dotted lines connect the fitted values with the measured data points. Source data are provided as a Source Data file.

Our previous findings on our study population have identified an age-dependent accumulation of iron in specific thalamic regions in AGU^5^. Iron analysis was carried out from the brain MRI data as an exploratory outcome not mentioned in our study plan, as the method was originally developed using the 0-month data on our study patients. For the analysis of iron accumulation over the study course, we improved our published method that facilitates a quantitative assessment of iron, based on susceptibility-weighted (SW) brain MRI. As there are no long-term quantitative data on iron accumulation in AGU, we constructed two age-based prediction models, using the data of our 21 AGU patients of different ages at study begin (0 months) (Fig. 8). The quantitative iron accumulation data were derived from the MRI of the 21 original patients. In some patients, the baseline data were not available due to movement artefacts in the MRI images, but these data were fitted using the model assumption values for the respective patient age (marked as dashed lines in Fig. 8). For the patients that required anesthesia during the MRI, data points for 0, 24, and 48 months were available, whereas for the other participants, 5 data points (0, 12, 24, 36, and 48 months) were used. The four patients that joined the study after the first year were not included, as the follow-up time was too short to allow a reliable analysis. Furthermore, the youngest patients (3 and 4 years of age) did not show any substantial iron accumulation at study begin.

The model according to Gompertz postulates that a plateau in iron accumulation is reached upon adolescence (around 15 years of age) (Fig. 8a). For almost all of the study participants, iron accumulation was decreased over the study course, with the values falling outside of the predicted 95% confidence interval area (grey area in Fig. 8). Only two patients (numbers 5 and 21) exhibited a minor increase in iron accumulation. A linear model postulating a continuous iron accumulation (Fig. 8b) supports the findings of the Gompertz model. These data suggest that Cystadane may prevent the accumulation of iron in the thalamic regions of AGU patients.

Taken together, the results of our clinical phase Ib/II study show that oral Cystadane/betaine in the treatment of AGU results in a significant reduction of urine GlcNAc-Asn excretion, with a concomitant increase in the serum AGA activity. In addition, our novel biomarker, iron accumulation in the thalamus, was decreased in most patients over the study course. Furthermore, improvements of cognitive and adaptive skills were detected in most areas in validated neuropsychological tests, WISC-IV and ABAS-3. In addition, Cystadane/betaine is safe and well tolerated in pediatric AGU patients.

## Discussion

The purpose of our investigator-initiated, phase Ib/II study was to explore the efficacy of betaine (Cystadane) in AGU, using a drug repurposing approach. In addition, the safety was assessed. Our study was partly carried out during the worldwide Sars-Cov2 pandemic period, and local restrictions in traveling and meetings between the members of our study group placed specific challenges to our study. However, we managed to complete the study for 21 patients, whereas the four patients enrolled in 2019 did not complete the study due to study closure in April 2022, resulting in numerous missing data points for this group. Therefore, we decided to treat this group as dropouts and only included the original 21 AGU patients for the final data analysis.

An important feature of our study is the study duration of four years, which facilitated the assessment of clinical efficacy. Too often, clinical studies for drug repurposing in rare diseases are either interrupted after 1 or 2 years, or only planned to last for a period that may be too short for assessing the clinical benefits. Our study is distinctive, as the exceptionally long observation time of 4 years allows a more reliable clinical assessment of the benefits of the treatment, even though many of our endpoints showed significant efficacy measures already after a relatively short study period (see below). However, especially the behavioral/neuropsychological and MRI-based endpoints may require longer observation times. Thus, our results stress the importance of a meaningful study design and encourage a longer duration of clinical studies in rare diseases, in which the number of eligible patients is usually low.

The primary endpoint of the study, urine GlcNAc-Asn reduction, was met, as a significant reduction in urine GlcNAc-Asn was observed already after 6 months of treatment. During the 3-month treatment break between months 12 and 15, which was used to replace a placebo group, GlcNAc-Asn accumulation in the urine was increased, but it did not quite reach the baseline level. Dose escalation after the break further enhanced the GlcNAc-Asn reduction in almost all patients. Also, the main secondary efficacy endpoint, serum AGA activity, was met, with a moderate but significant increase in AGA activity already after 3 months in most patients. As with GlcNAc-Asn accumulation, AGA activity returned close to the baseline level during the treatment break, and it increased again after restarting the medication. However, there were large variations in the AGA activity between the patients. Taken together, both AGU-related main surrogate biochemical biomarkers significantly improved during betaine/Cystadane treatment.

Our neuropsychological studies revealed a significant improvement of raw scores in some subscales of the WISC-IV^44,45^ in the patient cohort. Nevertheless, there were substantial differences between the individuals, with some participants being unable to perform some subtests. The development of the raw scores did not correlate with the age of the subject at study begin^3^. Far more, those with the best scores at baseline exhibited the highest increase in the scores during the treatment period. However, previous studies have suggested that AGU patients normally do not show a positive cognitive development between 7 and 15 years of age, and the difference to age-matched, healthy controls, assessed as standardized scores, becomes even higher over the years^3^. Due to this, and different from our original study plan, we decided to focus on the raw scores for WISC-IV, as it would be unreasonable to expect that patients already showing substantial cognitive decline would show improvements that would allow them to catch up with their age-matched controls. Far more, we expected that we might observe a stabilization of the developmental state upon treatment, instead of the expected further decline. However, there was a positive correlation between the improved motor function, assessed with an exploratory jumping test, and the positive development of the raw scores in WISC-IV. This is in sharp contrast with the natural history studies that show an age-dependent cognitive decline that often culminates in severe physical disability and loss of independent skills beyond the age of 25^1,2,52^. Therefore, our findings indicate that Cystadane/betaine treatment exerts a positive effect on motor function and some subdomains of the cognitive capabilities in AGU. To gain further evidence, longitudinal studies would be required, as 4 years may be too short to observe all positive effects of the treatment on cognitive and motor development, especially in older patients.

In addition to WISC-IV, the ABAS-3 questionnaire^46^ filled out by the parents showed significant positive developments in all three domains tested, indicating an improvement of adaptive skills during the treatment. Importantly, two subtests, communication and social, significantly improved, suggesting positive effects on the daily life of the families. Furthermore, the families subjectively reported various daily life changes upon the treatment, such as improved sleep, reduced aggression, and less behavioral problems, even though these parameters were not quantitatively assessed in our study.

We also performed a quality of life questionnaire^48^ over the study course, but the results were inconclusive, as the AGU patients generally were about equally satisfied as the control group with the aspects of life included in the questionnaire. However, this non-disease-specific questionnaire does not contain items that are especially relevant for AGU patients, e.g., sleep and behavioral problems^50,51,53^. Furthermore, the quality of life of AGU children, as evaluated by their parents, was already at a relatively good overall level at the beginning of the study, due to the special support they receive, e.g., at school.

Our study patients exhibited developmental defects typical for AGU patients in brain MRI studies, such as hypointensities in specific thalamic regions^4^. No significant visible changes in brain morphology, neither negative nor positive, were detected between the study begin and 48 months, indicating that the brain pathology did not worsen over the study course. Based on the MRI data collected in our study patients, we have previously identified a novel quantitative surrogate biomarker, iron accumulation, in specific thalamic regions of AGU patients, which strongly correlates with the age of the patient^5^. Based on these MRI data, we constructed two age-associated models to predict iron accumulation in pediatric AGU patients. During the treatment period, iron accumulation was significantly reduced in most patients, and the SWI signals fell below the predicted 95% confidence interval, providing further support for the efficacy of Cystadane in AGU. The direct causative effect of iron accumulation in the progression of developmental delay and loss of cognitive capabilities in AGU has not been studied, but findings from other neurodegenerative diseases, including lysosomal storage disorders, suggest that the brain iron load contributes to the progression of these diseases^54,55^.

Cystadane/betaine was found to be safe, with only minor drug-related adverse effects, such as temporary gastrointestinal symptoms and strange body odor, found only in a few patients, whereas no severe adverse effects were observed. Thus, Cystadane can be considered safe for use in AGU, even for pediatric patients. The limitations of our study include the relatively small and heterogeneous patient population, which is typical for rare diseases. However, we included all suitable (age, genotype) Finnish AGU patients in our study. In addition, we chose not to include a direct comparator group, as splitting the patient population to treatment vs. placebo groups would have resulted in insufficient group sizes and loss of statistical power. Our study patients also represented a large age span and different degrees of cognitive impairment. Optimally, a study focusing on a less heterogeneous sub-population and including an untreated control group should be performed. However, such a study design is hampered by the low number of AGU patients available.

The results of our clinical study show that Cystadane/betaine potentially provides a safe, easy to dose and effective treatment for AGU. Due to the benign adverse effects, Cystadane/betaine would be suitable for long-term therapy, as the nature of AGU would require a life-long treatment. Cystadane is an approved prescription drug, but anhydrous betaine in good quality is freely available as a powder from numerous vendors. Thus, betaine could be an alternative for Cystadane, especially in less developed countries, where Cystadane may not be easily available, resulting in a cost-effective treatment due to the low price of betaine. However, the treatment should always be carried out under the supervision of a clinician.

Our previous cell culture findings have shown that betaine can act as a true pharmacological chaperone for AGA, as it is capable of increasing the activity of mutated AGA^26^. However, the treatment-induced increase in the AGA activity in the serum of our AGU patients was relatively moderate and remained far below the level of an AGU carrier. On the other hand, we measured the AGA activity in the patient sera, as this was the most practical way to collect samples for the measurement. However, the activity increase may well be higher in the cells of the patients, as our assay only measures the active AGA that is secreted from the cells into the blood. Nevertheless, the question arises if the activity increase is enough to explain the positive development observed for other biomarkers, such as urine glycoasparagines.

It is plausible that betaine exhibits further beneficial effects, beyond its function as a direct PC, that indirectly improve cellular survival and functions. The further potential effects of betaine on cellular function may be based on its function as an osmolyte^29,56^. Betaine regulates the cellular hydration state and cell volume, and many metabolic pathways, e.g., protein turnover and carbohydrate metabolism, are sensitive to various kinds of osmotic stress^57^. By protecting the cells against osmotic stress, betaine stabilizes native protein structures^32,58–61^, and it can also function as a chemical chaperone that generally assists in protein folding^62,63^. A positive effect of betaine on the gut microbiota and the brain-gut axis is also conceivable. Improvements of gut microbiota may exhibit positive effects on the behavioral aspects, and the importance of microbiota is well known in, e.g., autism spectrum diseases^64–67^. Thus, betaine may improve intestinal function and affect the diversity of microbiota, as shown in high-salt stressed animals and in an animal model of chronic social defeat stress^68,69^. Due to the potential non-PC functions of betaine in AGU, it thus may also be suitable for other types of variants in AGU, beyond the missense variants amenable to the PC function.

In conclusion, the results of our clinical study show that Cystadane/betaine is a safe and efficient treatment for AGU, for which no approved treatments are currently available. Due to its ease of use and safety, Cystadane/betaine is an excellent candidate for drug repurposing. Further studies should be carried out over a longer treatment period, which would facilitate a further assessment of the effect of the treatment on the cognitive skills of the patients. Furthermore, a long-term clinical study with AGU patients younger than our cohort would reveal if betaine is also capable of preventing the cognitive decline if the intervention is started early in life.

## Methods

### Clinical study conductance and approval

This study was conducted following the guidelines of the Declaration of Helsinki, Good Clinical Practice, and local regulations. The AGU-001 study was registered on May 31, 2017 at European Medicines Agency (EMA, EudraCT number 2017-000645-48; https://www.clinicaltrialsregister.eu/ctr-search/trial/2017-000645-48/FI), and approved by the respective Finnish authorities (Fimea; KLnro 56/ 2017) on June 29, 2017. The original study plan, its amendments, and all additional study documents were approved on October 16, 2017, by the ethical committee for women, children, and for psychiatry of the Helsinki and Uusimaa region, Finland, (permission number HUS/2604/2017). After obtaining all required approvals and permissions, 21 AGU patients were identified and recruited before the study begin on February 02, 2018. Sex and gender of the participants was not relevant for the study design due to the specific features of the study population. The amendments on the original study protocol were approved by the local authorities. The final study plan with the statistical plan and a list of the approved amendments are provided as Supplementary Information.

### Patient eligibility, recruitment, and enrollment

This study was an investigator-initiated, single-center, open-label, phase Ib/II interventional trial involving patients with a genetically confirmed diagnosis of AGU. The patients were identified in collaboration with the Finnish AGU patient organization, Suomen AGU r.y., and recruited by M.L. The study population represents a specific vulnerable population, as all patients were minors (below 18 years of age) who have a neurodevelopmental disorder with intellectual disability, and who are thus not capable of personally giving an informed consent. The informed consent form was signed by a parent or a person who legally represents the patient. The patients did not receive any compensation for their participation. Upon enrollment, the patients received a codified number that was used to track and label all the samples and the data obtained during the study. A database containing patient IDs and the codified numbers was established and kept by Dr. M. Laine in the University Hospital of Helsinki. The identities and personal data of the patients were only known for the medical personnel involved in the study.

All study patients were homozygous for the AGU_Fin-major_ variant, as confirmed by a previous genetic diagnosis. The age at diagnosis varied between 2 and 8 years. In the original study plan (EMA registration), we planned to enroll 15 AGU patients. However, the study plan was amended, as further eligible patients were identified, and 21 patients (age 7–15 years) were enrolled in the study in February 2018 for the duration of 48 months. Further four eligible patients between 3 and 17 years were identified during the first year of the study, and they were enrolled in May 2019 (at 15 months) after a respective amendment of the study plan, approved by the local authorities.

Exclusion criteria were: age above 17 years (amended from above 15 years, as described above), patients who do not carry at least one allele of the AGU_Fin-major_ variant, known adverse reactions against components of Cystadane, a co-medication that would interfere with Cystadane treatment, known history of cerebral oedema, participation in other interventional clinical studies, and prior bone marrow transplantation. However, none of these criteria was met by any of the screened patients. Inclusion and exclusion criteria are presented in the study protocol submitted to EMA and approved by Fimea/local ethical committee. Formal sample size calculation was not performed due to the low number of available patients, the specific characteristic of a rare disease, and the partially explorative character of the study.

### Study design and treatment

This open-label study was carried out with a successive dose escalation strategy. Figure 1a shows a CONSORT diagram for the study flow, and the time points of clinical assessments, blood and urine sampling, and the dose escalation strategy are shown in Fig. 1b. All patients underwent the first clinical assessment by a child neurologist (M.L.) at study begin, and started the oral Cystadane uptake either the same day or a few days later, after baseline MRI. Initially, the dose with 100 mg/kg/day was divided into two equal portions that were taken with food or a beverage. The initial dose was increased to 130 mg/kg/day at month 11 due to change in the recommendation of the maximum daily dose for Cystadane and a preliminary assessment of the biochemical endpoints with positive tendencies, as stated in the statistical plan. A treatment break was implemented for 3 months between months 12 and 15, after which the dose was gradually escalated to 200 mg/kg-day by month 36 (the protocol amendment approved by the local authorities). The dose escalation for the four patients recruited after year 1 was started after 3 months, and the escalation was completed after 24 months.

### Safety and adverse events

One of the objectives of this study was to assess the safety of betaine/Cystadane in the treatment of AGU. As a safety measure, plasma methionine levels were determined during the visits at 0, 3, 6, and 24 months. The safety limit for plasma Met was 700 µmol/L, which is 10 times the upper limit of the normal range. During the visits to the child neurologist, possible adverse events were assessed and recorded by interviewing the parents.

### Pre-defined endpoints and assessments

The primary endpoint of the study was the reduction of GlcNAc-Asn in the urine, and the secondary endpoints were serum AGA activity, absence of severe adverse events, plasma Met level, brain MRI findings, and neuropsychological test (WISC-IV), as well as quality of life surveys, and adaptive skills questionnaires.

### LC-MS based quantification of GlcNAc-Asn

Urine samples of the 21 original study participants were collected at 0, 3, 6, 12, 15, 24, 36, and 48 months (8 time points and samples per patient) and stored frozen as aliquots at −80 °C. Patient number 19 refused to give a urine sample at 48 months (only seven samples available for this patient). The samples were diluted 80-fold with water, and 20 µl of each sample was injected as technical triplicates for the quantification of urine GlcNAc-Asn. LC-MS analysis was performed with a Hypercarb column (3 µm particle size, column length 100 mm, inner diameter 2.1 mm; Thermo Fisher Scientific, Dreieich, Germany). Analytical mode separation was performed with 0.1% formic acid as the mobile phase, at a flow rate of 200 µl/min at 30 °C, 60–80 bar pressure, on an Alliance HT 2790 High-Performance Liquid Chromatography system (Waters GmbH, Eschborn, Germany). The LC equipment was coupled to a Waters Micromass ZQ 4000 mass detector (Waters GmbH) operated in the positive ion detection mode up to 900 m/z. For the quantification of GlcNAc-Asn, a selected ion signal at 358 m/z (corresponding to [GlcNAc-Asn + Na]^+^) was used, and the peak areas were recorded with the MassLynx software, version 4.0 (Waters GmbH). Aberrant read-outs resulting from sampling failures were excluded from the data analysis, resulting in technical duplicates instead of triplicates in some cases (see enclosed Supplementary Data 1 file). Different amounts (39 pmol to 1.2 nmol) of pure GlcNAc-Asn (Santa Cruz Biotechnology, Heidelberg, Germany) were injected in triplicates, and the mean peak areas of the triplicates were used to calculate a calibration curve. The GlcNAc-Asn amounts in the urine samples were determined with the quantification tool of the MassLynx software, using the mean peak area of each urine sample. Urine creatinine was measured as technical triplicates with the Creatinine (Urinary) Colorimetric Assay Kit (Cayman Chemical, Biomol GmbH, Hamburg, Germany) according to the manufacturer’s protocol. The urine GlcNAc-Asn amounts were normalized to creatinine and expressed as mg GlcNAc-Asn per gram creatinine.

### Serum AGA activity

AGA activity was measured from serum samples with our validated fluorometric assay, as described^40^. Reaction mixtures consisted of 20 µL sample and 20 µL of Asp-AMC (25 µM, L-Aspartic acid β-(7-amido-4-methylcoumarin), Santa Cruz Biotechnology) in McIlvain’s phosphate-citrate buffer, pH 6.5. Artificial serum/plasma II (Cat. ASP-002, Biopanda Diagnostics, Belfast, UK) was used as a blank. Samples were incubated for 24 h at 37 °C, after which the reaction was terminated by adding 200 µL McIlvain’s buffer, pH 4.5. All samples were measured as technical triplicates with a Tecan Infinite M200 plate reader and the software Tecan i-control, Version 3.9.1.0 (Tecan, Männedorf, Switzerland), using 355 nm excitation and 460 nm emission wavelengths. AMC (7-Amino-4-methylcoumarin; Sigma-Aldrich, Taufkirchen, Germany) was used for the preparation of standards containing increasing amounts of AMC in artificial serum, ranging from 0 to 100 pmol AMC. Because of the background fluorescence of Asp-AMC, defined amounts of Asp-AMC substrate solution were added so that the total amount of AMC and Asp-AMC was identical in all standards. For the calculation of AGA enzyme activity, the mean blank fluorescence was subtracted from all samples. According to the slope of the standard curve, the AGA activity values are expressed as mU/L (1 mU = 1 nmol/min, 24 h corresponding to 1440 min).

### Magnetic resonance imaging

We imaged 21 patients (11 females and 10 males, 7.4–15.1 years of age) and 21 healthy controls (9 females and 12 males, 7.1–15.2 years) with MR imaging (3 T Magnetom Skyra; Siemens, Erlangen, Germany). The protocol included the following sequences: T1-weighted 3D-MPRAGE (repetition time, TR = 2000 ms, echo time, TE = 2.74 ms, flip angle = 10°, thickness = 1 mm, matrix = 256 × 256), FLAIR 3D (TR = 5000 ms, TE = 386 ms, inversion time, TI = 1800 ms, flip angle = 120°, thickness = 1 mm, matrix =  244 × 244), T2-weighted TSE axial (TR = 4000 ms, TE = 82 ms, flip angle = 150°, thickness = 3 mm, matrix = 448 × 448), diffusion-weighted imaging (TR = 3700 ms, TE = 108 ms, flip angle = 90°, thickness = 2.4 mm, matrix = 98 × 96, in a total of 113 gradient orientations with three diffusion-weightings: 800 s/mm^2^, 1500 s/mm^2^, and 2800 s/mm^2^), and susceptibility-weighted imaging (SWI, TR = 27 ms, TE = 20 ms, flip angle = 15°, thickness = 2 mm, matrix = 256 × 232). Most participants, including patients and controls, underwent annual imaging, except for those requiring anesthesia, who were only imaged bi-annually.

### Neuropsychological testing

The participants were assessed with the Wechsler Intelligence Scale for Children-IV^44,45^ (WISC-IV), which is based on four indices: verbal comprehension (VCI), perceptual reasoning (PRI), working memory (WMI), and processing speed (PSI). Core subtests included similarities, vocabulary, comprehension for VCI; block design, picture concepts, matrix reasoning for PRI; digit span, letter-number sequencing for WMI; and coding, symbol search for PSI. All other tests are age-independent, whereas the PSI tasks differ by age. Children aged 8–16 years complete the PSI standard version B, which was used for the participants of the study. The raw scores were compared with the values of a large Finnish cohort.

### Adaptive skills

Adaptive skills were assessed with the Adaptive Behavior Assessment System, third version (ABAS-3)^46^ using a translation in Finnish. Parents annually scored the different domains (conceptual, social, and practical) of the questionnaire, and the raw scores were calculated.

### Quality of life questionnaire

The Kid- & Kiddo KINDL quality of life questionnaire has been developed in Germany for epidemiological use in children and adolescents aged 3–17 years^48,49^. It consists of 24 questions (4 questions per subscale) that were answered by the parents. The six subscales are: physical well-being, emotional well-being, self-esteem, family, social contacts, and school. Each question addresses experiences from the previous week and was answered by the parents on a scale from 1 to 5 (1 = never, 2 = seldom, 3 = sometimes, 4 = often, and 5 = always). For the analysis, the scores were converted as percentage scale from 0 to 100%. For reference, the results of the German cohort that contains data from 14,836 children and adolescents were used^50,51,53^. In general, younger children achieve higher scores than the 11–17 years age group^50,51,53^.

### MRI–statistical analysis of SWI data

Our previous study demonstrated that the thalamic regions with iron accumulation in AGU patients can be detected using a voxel-wise statistical permutation test^5^. In this study, we revised the previous analysis method in two ways. First, the revised method is more patient-specific, as individual structural T1-image segmentation is used to obtain the location of the thalami^70–72^ and for the study-specific template generation to register all magnetic resonance images to the same coordinate system for the voxel-wise analysis^73^. Second, the revised method is more robust in locating the iron-accumulation regions within the thalami, as it is based on a leave-one-out cross-validation during the statistical permutation test. In each test, one of the patients was left out in the permutation test, and the average of all outcomes was used as the iron-accumulation region in the subsequent analyses. Linear model and the Gompertz model were fitted to the extracted phase signals of susceptibility-weighted images using an in-house Python code^74,75^ to obtain 95% prediction intervals, coefficient of determination (*R*^2^), and mean-squared error (MSE). The code is available under https://github.com/vilsaira/AGU_SWI_analysis and at https://doi.org/10.5281/zenodo.22141812^76^.

### Statistical analysis of biochemical markers

Due to the extremely low prevalence of the disease, a sample size estimate was not performed in advance. The combined data of the 21 original patients at all available time points were analyzed, resulting in an unbalanced panel data set for 21 patients over the eight observation periods with a total of 167 (for urine GlcNAc-Asn) and 157 (for serum AGA enzyme activity) observations. The starting point of the observation was the patient’s baseline value at 0 months (untreated). The Cystadane medication was discontinued between months 12 and 15; the 15-month samples thus representing a post-washout value, whereas the 3, 6, 12, 24, 36, and 48-month data are designated as “treated” samples. Hence, the observations are clustered within a subject, i.e., the observations and their residuals are correlated and not independent. To account for the repeated measures design, a linear mixed model (LMM) was fitted to the data using the software R Core Team (https://www.R-project.org and http://www.rstudio.com) with the packages of Bates et al.^77^, Kuznetsova et al.^78^, and Hothorn et al.^79^. Within this approach, individual patients were considered as random effects, and their scores were compared as a function of the fixed effect of the status untreated (study begin), post-washout (15 months), or treatment. To obtain a normal distribution of the residuals, the data were ln-transformed for the LMM.

Statistical analysis of the neuropsychological data and the adaptive skills was done with 1-way ANOVA (Analysis of Variance) with Dunnett’s multiple comparisons, using GraphPad Prism software version 11 (San Diego, CA, USA). Values of *p* < 0.05 were considered significant (*), while values of *p* < 0.01 were considered very significant (**) and *p* < 0.001 extremely significant (***). The exact *p* values are depicted in the figures and their legends.

### Reporting summary

Further information on research design is available in the Nature Portfolio Reporting Summary linked to this article.

## Supplementary information

Supplementary Information

Description of Additional Supplementary Files

Supplementary Data 1

Reporting Summary

Transparent Peer Review File

## Source data

Source Data

## Data Availability

Individual participant MRI data that underlie the results reported in this article are available after de-identification. These data will be shared with investigators whose proposed use of the data has been approved by an independent ethical committee that should be disclosed upon submitting an application for the data use as disclosed in the application. Applications should be submitted to Ritva.Tikkanen@biochemie.med.uni-giessen.de. The data are available beginning 6 months and ending 3 years following article publication. Study protocol with statistical analysis plan, informed consent forms, and all other de-identified data are available as Supplementary Information. Source data are provided with this paper.

## Code availability

The MRI analysis codes are freely shared via GitHub. https://github.com/vilsaira/AGU_SWI_analysis and https://doi.org/10.5281/zenodo.22141812^76^.
