# Supplementary material for: Pharmacological chaperone treatment with Cystadane in aspartylglucosaminuria: an open-label, phase Ib/II trial
Source: Nat Commun. 2026 Nov 11;17:11604. doi: 10.1038/s41467-026-78752-x (PMC13754814; doi:10.1038/s41467-026-78752-x)
Supplement: Supplementary file 1 [file 41467_2026_78752_MOESM1_ESM.pdf]

Supplementary Information

Clinical Data

AGU Diagnosis. The diagnosis was done by analysis of urine oligosaccharides (glycoasparagines) and was confirmed by genetic testing of the *AGA* gene. In three families, there were two siblings with AGU diagnosis. All enrolled patients have the same homozygous $AGU_{Fin\ major}$ variant.

Other medication. AGU patients typically exhibit severe behavioral problems and use different types of medication. In our cohort, 8 out of 25 patients had concomitant behavioral medication (risperidone, aripiprazole, methylphenidate and/or levomepromazine), and 7 out of 25 patients had at least intermittent, concomitant use of melatonin, hydroxyzine (an antihistamine) and/or carbamazepine for sleeping problems.

Further clinical findings. A common bone problem, genu valgus, was surgically corrected by a metal plate during growth in one or both legs in some patients. Astigmatism is common among AGU patients, and about one-third of the cohort used glasses. Two patients had a previous diagnosis of a mitral valve insufficiency, and one patient had an aortic valve insufficiency.

Supplementary Table 1. Serum Methionine Levels. Met levels in the serum were determined as a safety measure. The values, given as µmol/L, were compared with the age-adequate reference values (below 70 µmol/L for children up to 16 years, and below 89 µmol/L for persons older than 16). The * indicates a value above the upper reference limit. Values below 700 µmol/L were considered safe.

	Serum methionine levels (µmol/L)			
Patient	**0 months**	**3 months**	**6 months**	**24 months**
1	54	31	40	36
2	33	44	31	33
3	36	27	34	30
4	21	26	26	37
5	21	43	41	25
6	29	29	27	30
7	40	32	31	36
8	40	32	31	36
9	36	28	27	30
10	25	43	45	43
11	29	55	37	33
12	27	44	48	41
13	33	32	30	25
14	42	50	42	41
15	19	33	32	33
16	31	42	50	**89***
17	33	37	35	47
18	27	40	40	43
19	30	40	49	38
20	22	34	37	43
21	25	32	26	26

Supplementary Table 2. Scores of the Kid- and Kiddo-KINDL questionnaire. The total scores of the six dimensions for each patient and time point are shown.

Emotional well-being

Patient	Timepoint/months				
	0	12	24	36	48
1	75	75	81	69	50
2	56	75	81	69	63
3	63	69	81	69	75
4	63	69	69	63	69
5	63	63	75	69	75
6	56	50	56	56	56
7	69	69	75	63	75
8	88	88	81	75	81
9	75	75	69	75	75
10	94	88	72	56	50
11	88	75	69	81	75
12	50	75	88	75	88
13	81	94	69	44	69
14	81	75	88	81	75
15	69	72	75	81	75
16	44	69	81	81	75
17	75	69	75	81	88
18	75	75	66	56	69
19	69	69	69	69	69
20	88	81	81	69	75
21	81	94	88	88	88

Family

Patient	Timepoint/months				
	0	12	24	36	48
1	81	63	81	63	69
2	38	63	63	81	69
3	75	75	81	75	81
4	44	63	56	56	44
5	63	69	69	69	69
6	50	44	44	50	38
7	75	69	75	75	88
8	94	94	81	75	88
9	75	75	75	63	63
10	63	69	69	69	44
11	75	81	75	81	69
12	81	81	69	75	75
13	88	88	94	81	94
14	81	69	94	100	94
15	63	66	69	75	75
16	75	81	69	75	88
17	88	63	78	94	100
18	44	81	78	75	50
19	50	81	81	88	88
20	81	63	75	69	69
21	81	81	94	94	84

Physical well-being

Patient	Timepoint/months				
	0	12	24	36	48
1	81	63	81	69	69
2	88	88	94	81	56
3	75	88	88	69	69
4	88	94	88	63	94
5	69	94	88	88	81
6	69	75	63	75	88
7	63	88	81	81	75
8	56	50	88	88	75
9	75	75	63	56	88
10	94	88	69	50	69
11	75	44	63	75	63
12	31	75	88	75	100
13	81	75	81	63	56
14	81	81	81	75	69
15	56	72	88	75	75
16	56	81	100	94	69
17	56	50	69	88	94
18	88	88	88	88	88
19	63	81	81	69	69
20	69	100	75	88	88
21	92	75	100	75	63

School

Patient	Timepoint/months				
	0	12	24	36	48
1	81	94	100	94	75
2	88	88	88	88	88
3	69	88	81	88	88
4	81	88	88	81	88
5	81	100	81	81	88
6	81	69	63	75	69
7	81	88	81	75	88
8	88	88	88	81	88
9	88	88	88	88	88
10	75	88	72	56	88
11	81	81	88	88	100
12	94	88	84	81	100
13	88	81	100	75	88
14	94	81	94	88	88
15	81	81	81	94	75
16	81	81	75	75	75
17	88	81	78	75	94
18	81	84	84	80	75
19	75	75	75	88	88
20	100	88	88	94	81
21	84	88	88	88	88

Self-esteem

Patient	Timepoint/months				
	0	12	24	36	48
1	75	69	81	75	75
2	75	69	69	75	63
3	75	75	75	75	75
4	69	63	69	69	63
5	56	69	69	69	69
6	69	69	50	50	69
7	69	75	75	63	69
8	75	75	75	75	75
9	63	63	56	56	69
10	56	69	72	75	63
11	81	69	81	69	75
12	75	81	81	75	94
13	75	69	69	44	69
14	63	56	50	69	56
15	50	53	56	69	69
16	63	63	63	69	63
17	88	75	75	75	100
18	69	69	59	50	75
19	75	56	69	94	94
20	88	88	75	81	75
21	63	75	88	69	69

Social contacts

Patient	Timepoint/months				
	0	12	24	36	48
1	69	75	75	50	50
2	50	75	75	63	50
3	56	56	63	69	75
4	75	75	69	69	56
5	56	75	69	75	63
6	38	44	44	38	44
7	63	69	69	69	69
8	81	81	81	75	81
9	69	75	59	63	63
10	63	69	59	50	56
11	75	75	69	69	81
12	69	88	84	81	81
13	69	75	69	63	63
14	44	50	69	75	75
15	31	50	69	81	75
16	31	63	56	63	19
17	75	50	66	81	94
18	69	75	75	75	75
19	81	81	75	94	94
20	88	88	81	81	81
21	69	75	81	81	75

Supplementary Figure 1

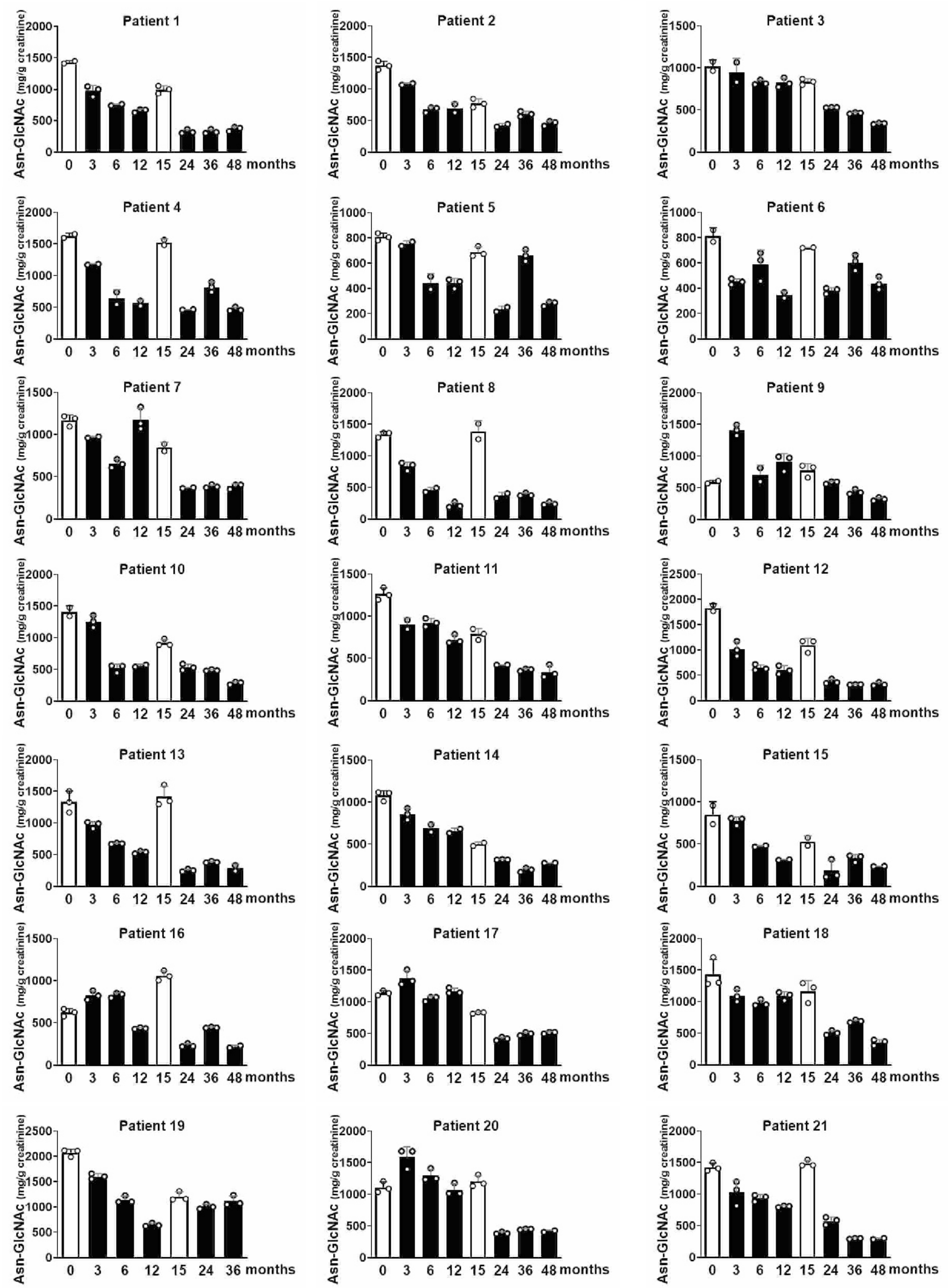

Supplementary Figure 1. Urine GlcNAc-Asn in individual patients. Bar graphs show the amount of excreted GlcNAc-Asn for each of the 21 study participants at all time points, measured as technical triplicates, shown as individual data points. To compensate for unequal fluid intake and urine output, all values were normalized to creatinine. White bars indicate the values prior to treatment (0 months) and after the 3 months washout period (at 15 months). n=1 sample per time point. Bars show mean values + SD. Patient number 19 did not provide a sample for the 48 months time point. Source data are provided with this paper.

Supplementary Figure 2

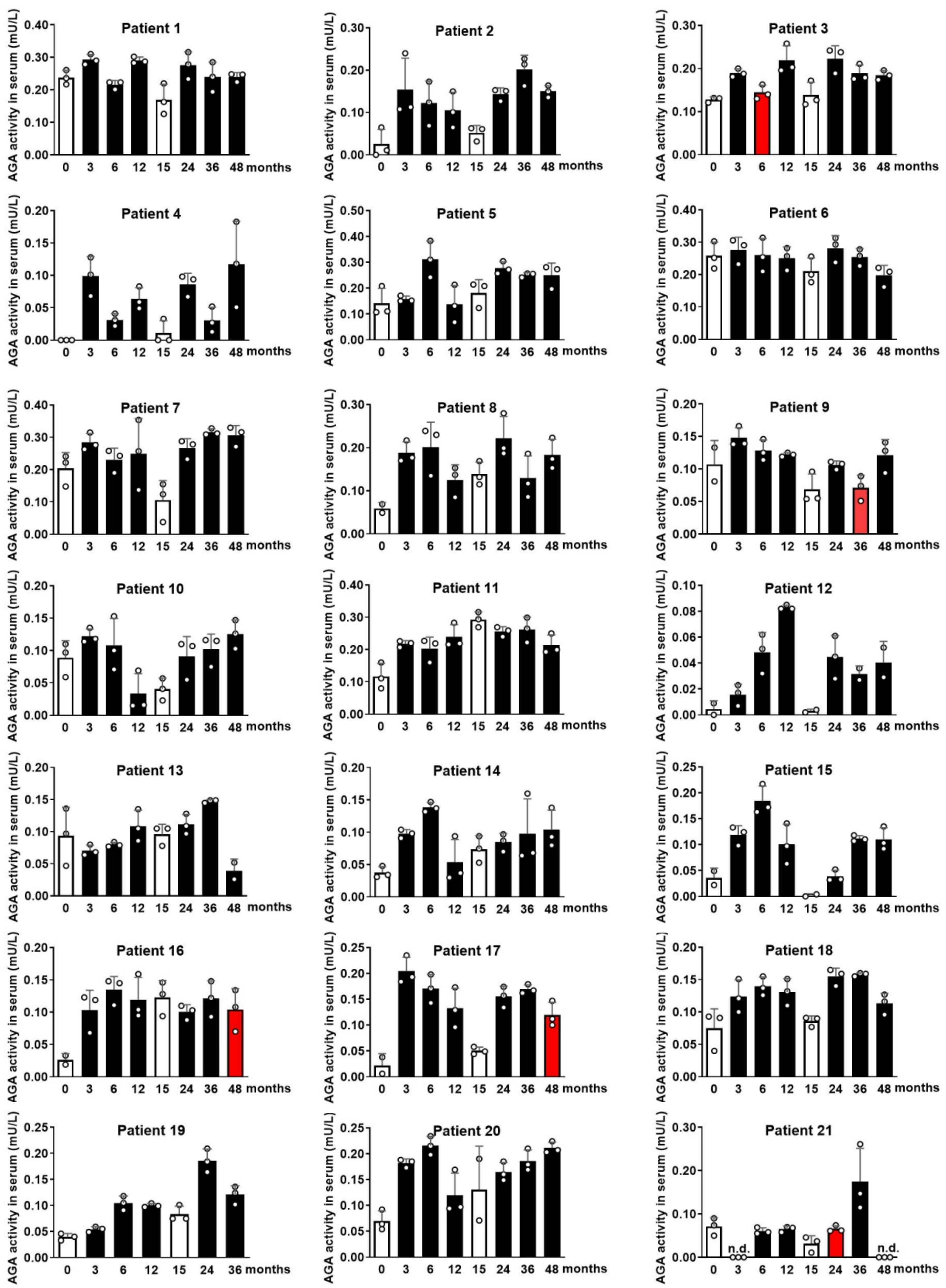

Supplementary Figure 2. Serum AGA activity in individual patients. Bar graphs show the enzyme activity of AGA in the serum for each of the 21 study participants at all time points, measured as technical triplicates that are shown as individual data points. Samples with hemolysis are marked with red bars. White bars indicate the values prior to treatment (0 months) and after the 3 months washout period (at 15 months). In some samples, AGA activity was below detection limit due to strong hemolysis (n.d. = not detectable). n=1 sample per time point. Bars show mean values + SD. Patient number 19 did not provide a sample for the 48 months time point. Source data are provided with this paper.

Supplementary Figure 3

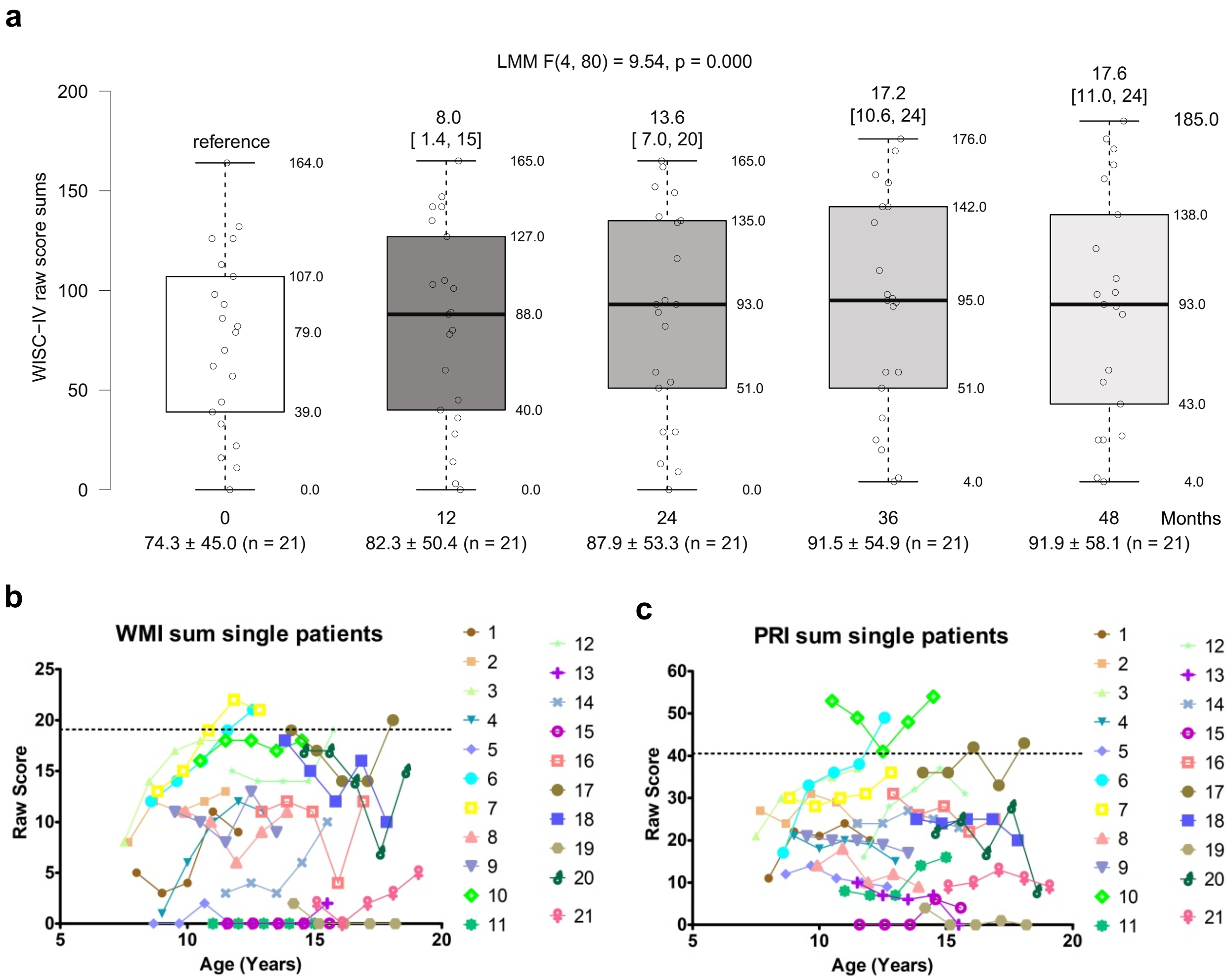

Supplementary Figure 3. The WISC-IV raw score developments over the study course. (a) Total raw scores analyzed using the LMM model, comparison of individual time points is shown. The available data points were converted to degrees of freedom, and a statistical analysis was performed to investigate whether betaine treatment had a significant effect on WISC-IV raw scores. The graphs show all individual data points and the aggregated trends. Box plots show the median (line within the box) and the 25th to 75th percentiles (interquartile box). The whiskers cover 1.5 times the interquartile range. To account for the repeated measures design, a linear mixed model (LMM) was fitted to the data. The LMM was evaluated using a standard ANOVA approach with F-test (F), resulting in two-sided p values. No adjustments for multiple hypotheses testing were performed. Comparison values shown on top of each whisker depict effect size differences to the reference [95%-CI], as estimated by the LMM. n=21 for all time points. Raw scores for the subscales WMI (b) and PRI (c) in relation to the patient age. The dotted lines in b and c represent the 6-year-old Finnish reference group. n=1 for each data point. Source data are provided with this paper.

Supplementary Figure 4

a

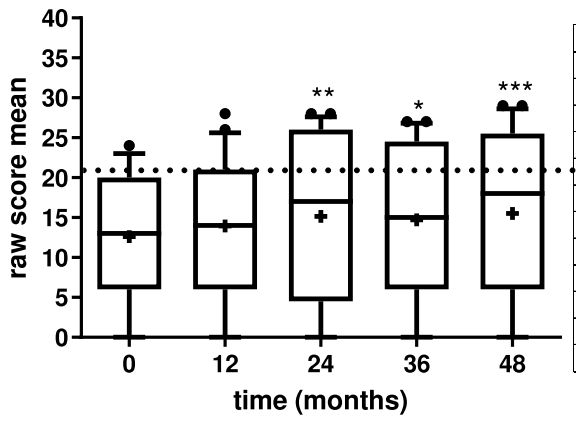

	0	12	24	36	48
Number of values	21	21	21	21	21
Minimum	0	0	0	0	0
25% Percentile	6	6	4.5	6	6
Median	13	14	17	15	18
75% Percentile	20	21	26	24.5	25.5
Maximum	24	28	28	27	29
Mean	12.62	13.95	15.14	14.71	15.52
Std. Deviation	8.225	8.93	10.29	9.461	10.31
Std. Error of Mean	1.795	1.949	2.245	2.065	2.249
Lower 95% CI of mean	8.875	9.887	10.46	10.41	10.83
Upper 95% CI of mean	16.36	18.02	19.83	19.02	20.22
p-value		0.227623	0.004055	0.021734	0.00076

b

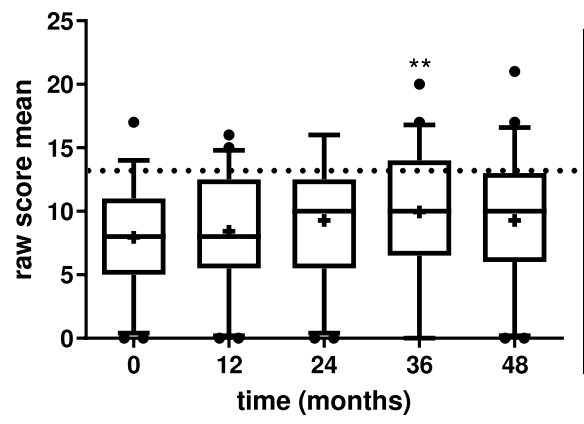

	0	12	24	36	48
Number of values	21	21	21	21	21
Minimum	0	0	0	0	0
25% Percentile	5	5.5	5.5	6.5	6
Median	8	8	10	10	10
75% Percentile	11	12.5	12.5	14	13
Maximum	17	16	16	20	21
Mean	7.952	8.429	9.286	9.952	9.286
Std. Deviation	4.555	4.884	4.931	5.527	5.658
Std. Error of Mean	0.994	1.066	1.076	1.206	1.235
Lower 95% CI of mean	5.879	6.205	7.041	7.437	6.71
Upper 95% CI of mean	10.03	10.65	11.53	12.47	11.86
p-value		0.850602	0.10192	0.005687	0.10192

c

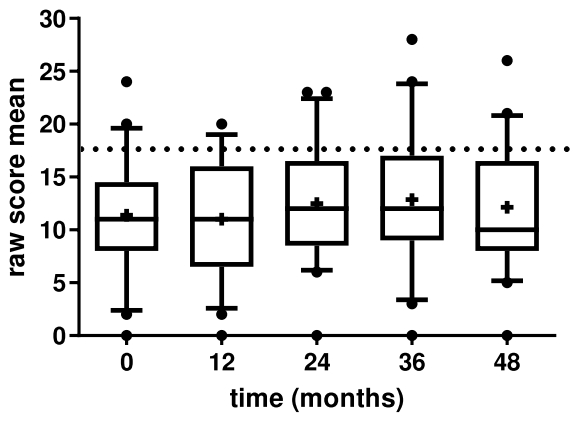

	0	12	24	36	48
Number of values	21	21	21	21	21
Minimum	0	0	0	0	0
25% Percentile	8	6.5	8.5	9	8
Median	11	11	12	12	10
75% Percentile	14.5	16	16.5	17	16.5
Maximum	24	20	23	28	26
Mean	11.38	11	12.48	12.86	12.14
Std. Deviation	5.714	5.675	5.896	7.08	6.093
Std. Error of Mean	1.247	1.238	1.287	1.545	1.33
Lower 95% CI of mean	8.78	8.417	9.792	9.634	9.369
Upper 95% CI of mean	13.98	13.58	15.16	16.08	14.92
p-value		0.961267	0.390077	0.155674	0.694346

d

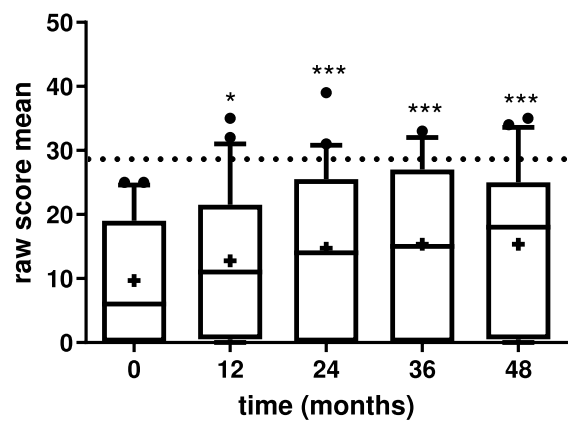

	0	12	24	36	48
Number of values	21	21	21	21	21
Minimum	0.00	0.00	0.00	0.00	0.00
25% Percentile	0.00	0.00	0.00	0.00	0.00
Median	3.00	4.00	6.00	5.00	10.00
75% Percentile	9.00	12.00	10.00	13.00	14.00
Maximum	13.00	18.00	20.00	18.00	18.00
Mean	4.857	6.429	6.238	6.667	7.571
Std. Deviation	5.023	6.653	6.024	6.583	6.735
Std. Error of Mean	1.096	1.452	1.315	1.436	1.47
Lower 95% CI of mean	2.571	3.4	3.496	3.67	4.506
Upper 95% CI of mean	7.143	9.457	8.98	9.663	10.64
p-value		0.2210	0.3252	0.1282	0.0092

e

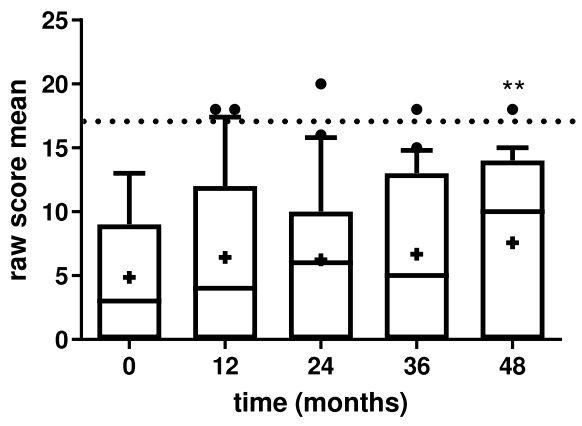

	0	12	24	36	48
Number of values	21	21	21	21	21
Minimum	0.00	0.00	0.00	0.00	0.00
25% Percentile	0.00	0.00	0.00	0.00	0.00
Median	3.00	4.00	6.00	5.00	10.00
75% Percentile	9.00	12.00	10.00	13.00	14.00
Maximum	13.00	18.00	20.00	18.00	18.00
Mean	4.857	6.429	6.238	6.667	7.571
Std. Deviation	5.023	6.653	6.024	6.583	6.735
Std. Error of Mean	1.096	1.452	1.315	1.436	1.47
Lower 95% CI of mean	2.571	3.4	3.496	3.67	4.506
Upper 95% CI of mean	7.143	9.457	8.98	9.663	10.64
p-value		0.2210	0.3252	0.1282	0.0092

Supplementary Figure 4. Individual sub-tests of the VCI and PSI indices. (a) VCI comprehension, (b) VCI similarities, (c) VCI vocabulary, (d) PSI coding, (e) PSI symbol search. Boxplots present the median and 25th -75th percentile, + indicates the mean value. Whiskers show the 10th-90th percentile. Data points outside of the 10th or 90th percentile are shown as dots. Repeated measures 1way ANOVA with Dunnett's multiple comparisons test vs. 0 months. ***p<0.001, **<0.01, *<0.05 vs. 0 months. The exact p values are listed on the right of the graphs, n=21 for all time points. The dotted lines represent the mean of the raw scores of the reference population (VCI: 6 years old, PSI: 8 years old). For the PSI subtest, the 8-year-old reference group was used, as this test is only used for children of 8 years and older. Source data are provided with this paper.

Supplementary Figure 5

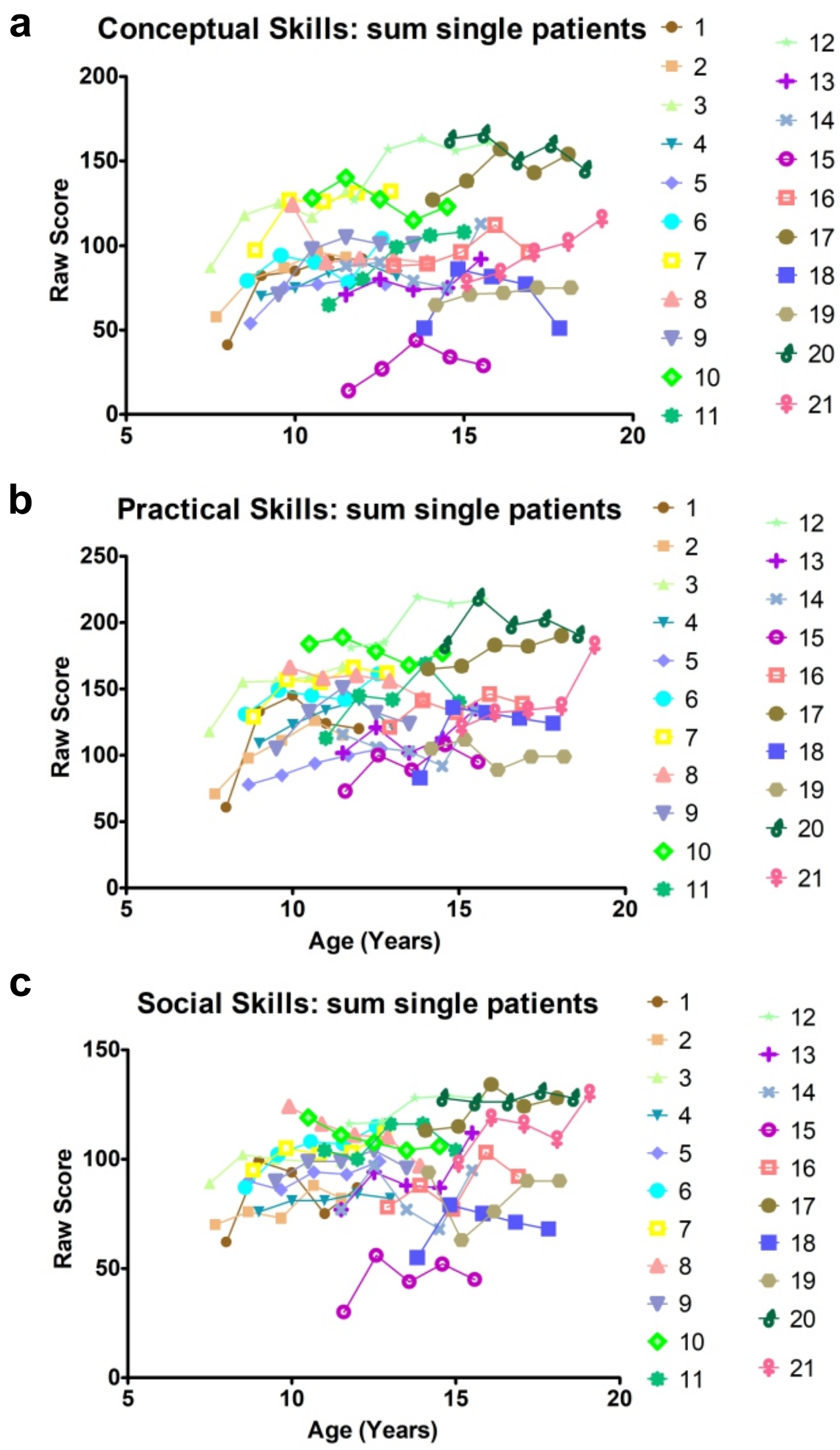

Supplementary Figure 5. The raw score development of adaptive skills over the study course for the individual patients. The parents annually completed the Finnish version of the adaptive behavior assessment system (ABAS-3) questionnaire during their visit at the neuropsychologist. The raw scores over the study course for the three domains (a) conceptual skills, (b) practical skills, and (c) social skills are shown for the individual patients in relation to the patient age. All data points are based on one measurement (n=1). Source data are provided with this paper.

Supplementary Figure 6

a

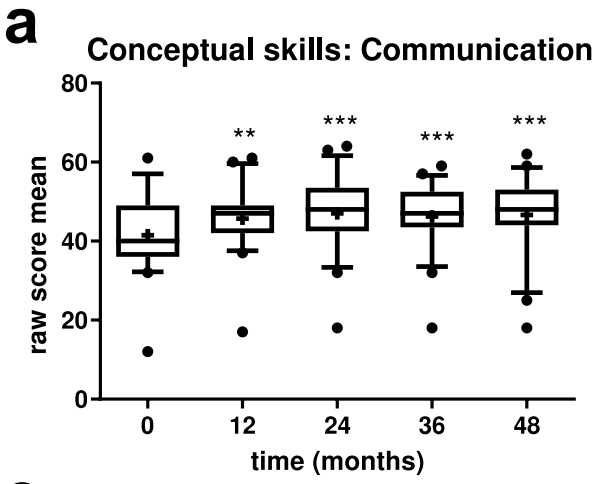

	0	12	24	36	48
Number of values	21	21	21	21	21
Minimum	12	17	21	18	18
25% Percentile	36	42	42.5	43.5	44
Median	40	47	48	47	48
75% Percentile	49	49	53.5	52.5	53
Maximum	61	61	64	59	62
Mean	41.48	45.71	47.05	46.24	46.62
Std. Deviation	10.85	9.15	10.08	9.011	10.47
Std. Error of Mean	2.367	1.997	2.2	1.966	2.284
Lower 95% CI of mean	36.54	41.55	42.46	42.14	41.85
Upper 95% CI of mean	46.41	49.88	51.64	50.34	51.38
p-value		0.003404	7.99E-05	0.000842	0.000286

b

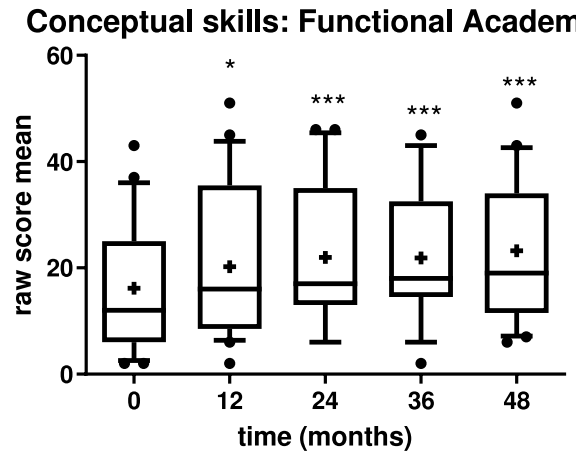

	0	12	24	36	48
Number of values	21	21	21	21	21
Minimum	2	2	6	2	6
25% Percentile	6	8.5	13	14.5	11.5
Median	12	16	17	18	19
75% Percentile	25	35.5	35	32.5	34
Maximum	43	51	46	45	51
Mean	16.19	20.24	21.95	21.86	23.24
Std. Deviation	11.84	14.32	13.6	12.65	13.21
Std. Error of Mean	2.584	3.126	2.967	2.761	2.883
Lower 95% CI of mean	10.8	13.72	15.76	16.1	17.23
Upper 95% CI of mean	21.58	26.76	28.14	27.62	29.25
p-value		0.017225	0.000347	0.000441	1.09E-05

c

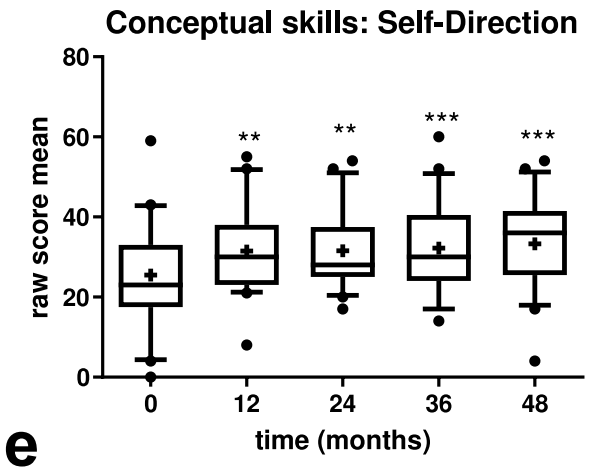

	0	12	24	36	48
Number of values	21	21	21	21	21
Minimum	0	8	17	14	4
25% Percentile	17.5	23	25	24	25.5
Median	23	30	28	30	36
75% Percentile	33	38	37.5	40.5	41.5
Maximum	59	55	54	60	54
Mean	25.48	31.52	31.57	32.24	33.29
Std. Deviation	14.21	11.65	10.14	12.07	12.05
Std. Error of Mean	3.101	2.543	2.212	2.634	2.631
Lower 95% CI of mean	19.01	26.22	26.96	26.74	27.8
Upper 95% CI of mean	31.95	36.83	36.19	37.73	38.77
p-value		0.003369	0.003092	0.000887	0.000106

d

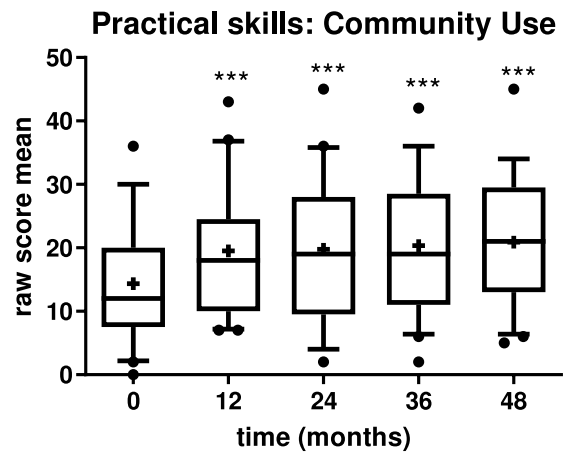

	0	12	24	36	48
Number of values	21	21	21	21	21
Minimum	0	7	2	2	5
25% Percentile	7.5	10	9.5	11	13
Median	12	18	19	19	21
75% Percentile	20	24.5	28	28.5	29.5
Maximum	36	43	45	42	45
Mean	14.33	19.52	19.76	20.33	20.9
Std. Deviation	10.09	10.47	12.04	10.92	10.83
Std. Error of Mean	2.201	2.285	2.627	2.383	2.364
Lower 95% CI of mean	9.742	14.76	14.28	15.36	15.97
Upper 95% CI of mean	18.92	24.29	25.24	25.3	25.84
p-value		0.000689	0.000367	7.59E-05	1.44E-05

e

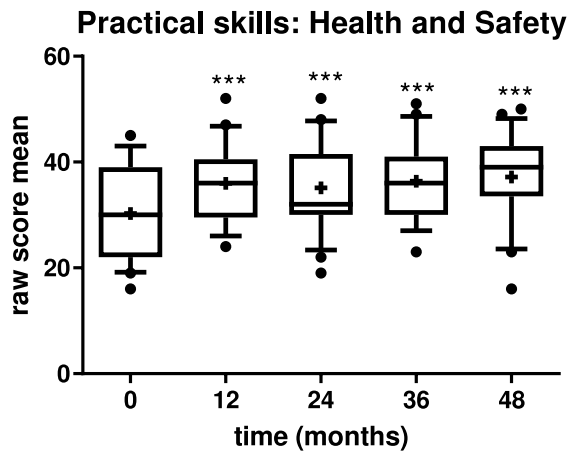

	0	12	24	36	48
Number of values	21	21	21	21	21
Minimum	16	24	19	23	16
25% Percentile	22	29.5	30	30	33.5
Median	30	36	32	36	39
75% Percentile	39	40.5	41.5	41	43
Maximum	45	52	52	51	50
Mean	30.29	35.95	35.14	36.38	37.19
Std. Deviation	8.906	7.599	8.398	7.639	8.34
Std. Error of Mean	1.943	1.658	1.833	1.667	1.82
Lower 95% CI of mean	26.23	32.49	31.32	32.9	33.39
Upper 95% CI of mean	34.34	39.41	38.97	39.86	40.99
p-value		1.09E-05	0.000169	2.36E-06	1.16E-07

f

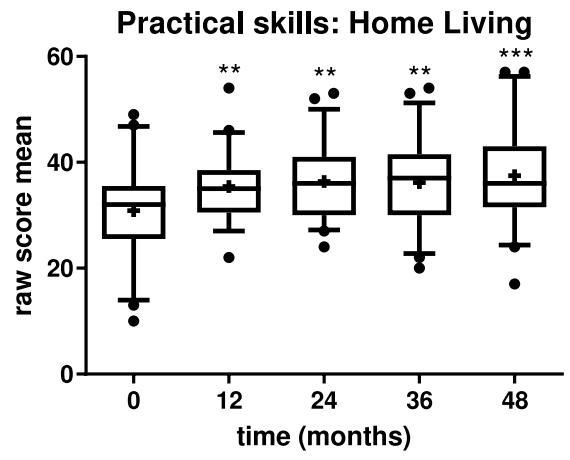

	0	12	24	36	48
Number of values	21	21	21	21	21
Minimum	10	22	24	20	17
25% Percentile	25.5	30.5	30	30	31.5
Median	32	35	36	37	36
75% Percentile	35.5	38.5	41	41.5	43
Maximum	49	54	53	54	57
Mean	30.86	35.43	36.38	36.19	37.48
Std. Deviation	10.43	7.27	7.533	8.761	10.21
Std. Error of Mean	2.276	1.587	1.644	1.912	2.228
Lower 95% CI of mean	26.11	32.12	32.95	32.2	32.83
Upper 95% CI of mean	35.61	38.74	39.81	40.18	42.12
p-value		0.008394	0.00105	0.001626	7.1E-05

g

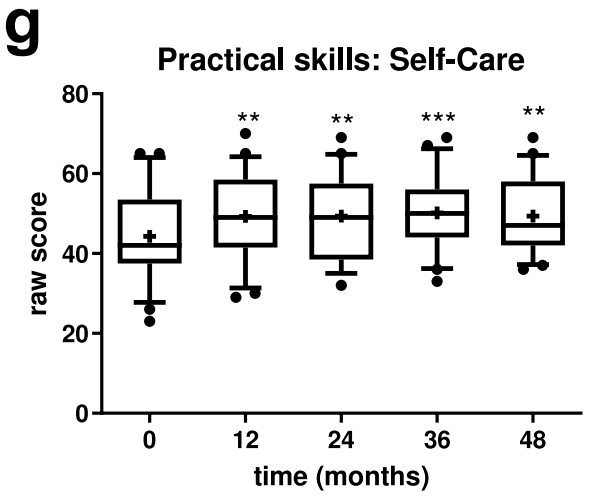

	0	12	24	36	48
Number of values	21	21	21	21	21
Minimum	23	29	32	33	36
25% Percentile	37.5	41.5	38.5	44	42
Median	42	49	49	50	47
75% Percentile	53.5	58.5	57.5	56	58
Maximum	65	70	69	69	69
Mean	44.29	49.29	49.38	50.19	49.33
Std. Deviation	11.52	11.02	10.88	9.569	9.901
Std. Error of Mean	2.514	2.405	2.374	2.088	2.161
Lower 95% CI of mean	39.04	44.27	44.43	45.83	44.83
Upper 95% CI of mean	49.53	54.3	54.33	54.55	53.84
p-value		0.002898	0.002341	0.000344	0.002606

h

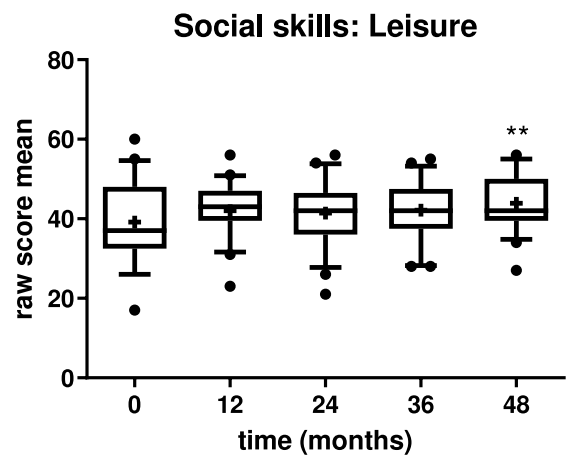

	0	12	24	36	48
Number of values	21	21	21	21	21
Minimum	17	23	21	28	27
25% Percentile	32.5	39.5	36	37.5	39.5
Median	37	43	42	42	42
75% Percentile	48	47	46.5	47.5	50
Maximum	60	56	56	55	56
Mean	39.19	42.05	41.43	42.14	43.9
Std. Deviation	10.85	7.256	8.617	7.908	7.476
Std. Error of Mean	2.367	1.583	1.88	1.726	1.631
Lower 95% CI of mean	34.25	38.74	37.51	38.54	40.5
Upper 95% CI of mean	44.13	45.35	45.35	45.74	47.31
p-value		0.14648	0.329607	0.127321	0.004925

i

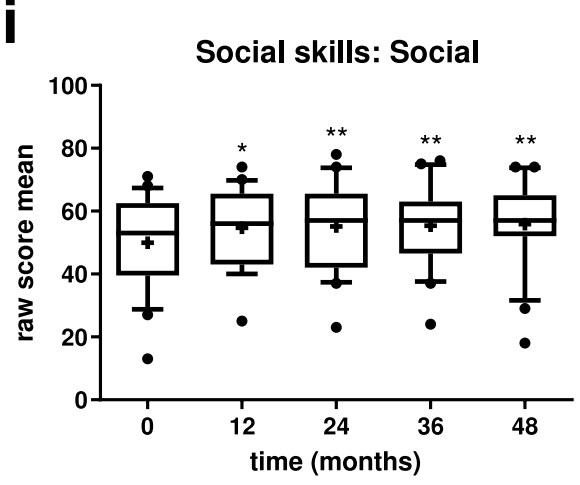

	0	12	24	36	48
Number of values	21	21	21	21	21
Minimum	13	25	23	24	18
25% Percentile	39.5	43	42	46.5	52
Median	53	56	57	57	57
75% Percentile	62.5	65.5	65.5	63	65
Maximum	71	74	78	76	74
Mean	49.95	54.67	55.14	55.33	55.86
Std. Deviation	14.56	12.58	14.07	12.96	14.28
Std. Error of Mean	3.178	2.744	3.07	2.827	3.116
Lower 95% CI of mean	43.32	48.94	48.74	49.44	49.36
Upper 95% CI of mean	56.58	60.39	61.55	61.23	62.36
p-value		0.013502	0.005558	0.00383	0.001312

Supplementary Figure 6. The raw score development over the study course for the ABAS-3 subscales. The subtests of the domains conceptual skills (a: communication, b: functional academics, c: self-direction), practical skills (d: community use, e: health and safety, f: home living, g:self-care) and social skills (h: leisure, i: social) were further analyzed. Boxplots present the median and the 25th -75th percentile, + indicates the mean value. Whiskers show the 10th-90th percentile. Data points outside the 10th-90th percentile are shown as dots. ***p<0.001, **<0.01, *<0.05 vs. 0 months. The exact p values are listed on the right of the graphs, n=21 for each time point. Repeated measures 1way ANOVA with Dunnett's multiple comparisons test vs. 0 months.
Source data are provided with this paper.

Supplementary Figure 7

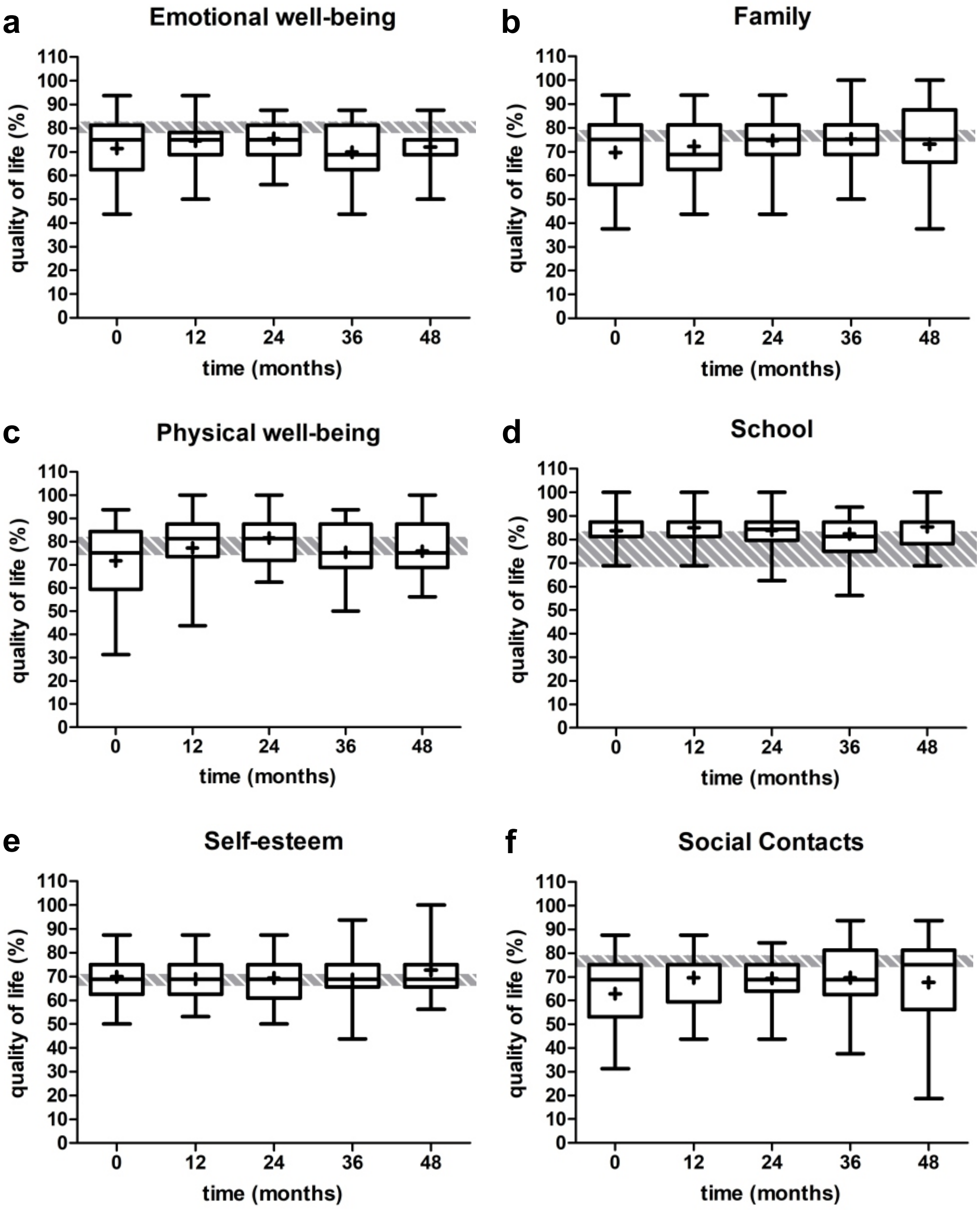

Supplementary Figure 7: Results from the KINDL quality of life analysis. The parents annually completed the parent-version of the KINDL-questionnaire. The results were converted to a 0 – 100% scale. The reference range of a healthy German cohort (children from 7 to 17 years) for each subscale is shown in shaded grey. (a) Emotional well-being (ref. range 79.1% - 82.3%); (b) Family (ref. range 76.3% - 79.8%); (c) Physical well-being (ref. range 73.3% - 80.5%); (d) School (ref. range 69.0% - 82.6%); (e) Self-esteem (ref. range 67.1% - 70.8%); (f) Social contacts (76.7% - 78.3%). Boxes show the 25th to 75th percentiles, and the line within the box the median value. The + depicts the mean, and whiskers indicate the minimum and maximum values. n=21 for all time points. Repeated measures 1way ANOVA with Dunnett's multiple comparisons test vs. 0 months. Source data are provided with this paper.

Open-label study to evaluate efficacy and safety of Cystadane for the treatment of aspartylglucosaminuria

EudraCT number: 2017-000645-48

Sponsor's Trial Identification Code: AGU-001, Version 1

Following major changes of the study protocol were approved by the local authorities in 2018 and 2019:

- The maximum possible number of patients to be included was increased first to 21 and then to 25 (from 15 patients that were estimated in the EMA registration form), due to identification of further potential participants, of which 21 were included in the study for 48 months, and 4 further ones after 15 months. The last four, however, never finished the study period of 48 months.

- The possible age range was expanded to 3 – 17 years (instead of 7 to 15 years).

- The measurement of AGA activity was done using serum (instead of white blood cells or plasma). This change was necessary due to sample handling and storage by the laboratory obtaining the blood samples.

- Dose escalation strategy was adjusted, as the maximum recommended daily dose of Cystadane was increased to 200 mg/kg/day (instead of 100 mg/kg/day at study begin).

- Interval of safety tests (Met in the blood) was adjusted (no test at 12 months).

- Use of age- and gender-matched controls for the MRI studies was included.

- Anna Tokola, Timo Roine and Ulrika Roine were included as persons to analyze the MRI data.

- Dr. G. Lochnit was added as a person to perform urine glycoasparagine measurement. This change (as compared to the registration at EMA) was already present in the first version of the written study plan sent to Fimea and the local ethical committee. It was necessary to change this point since the Finnish commercial diagnostics laboratory mentioned in the EMA submission did not offer the test as of 2018. Therefore, we changed the strategy already in the final study plan submitted to the local authorities.

- Dr. Elina Harjunen (psychologist) was added as a person to carry out the neuropsychological assessments & questionnaires, in addition to Päivi Helenius.

- Further time points for neuropsychological assessments & questionnaires were added, so that these studies were carried out annually (instead of biannually).

In the enclosed complete study plan, these changes are highlighted in yellow.

CLINICAL TRIAL PLAN

Open-label study to evaluate efficacy and safety of Cystadane for the treatment of aspartylglucosaminuria

Sponsors: Minna Laine and Ritva Tikkanen

EudraCT number: 2017-000645-48

Sponsor's Trial Identification Code: AGU-001

Sponsors

	Sponsor and Principle Clinical Investigator	Sponsor and Principal Scientific Investigator
Name and function	**Minna Laine, MD, PhD, child neurologist**	**Ritva Tikkanen, Professor of Biochemistry and Molecular Biology**
Address		
E-mail		
Phone		

Summary

The clinical study suggested here aims at testing the efficacy of Cystadane in Aspartylglucosaminuria in a pediatric patient cohort of 21-25 AGU patients between 3 and 17 years in Finland. Treatment with Cystadane/betaine has been shown to result in an increased AGA enzyme activity and improved lysosomal pathology in patient fibroblasts that carry the point mutations $AGU_{Fin\text{-}major}$ or Thr122Lys. The primary endpoint measurement is the excretion of glycoasparagines in urine, which is generally used as a prime clinical diagnostic criterion for AGU. Further, secondary endpoints are the measurement of AGA activity in serum, plasma Met concentration for safety, absence of severe adverse effects, neuropsychological evaluation and brain MRI. The study will be continued for a total of 48 months in order to be able to evaluate the benefit of Cystadane therapy for psychomotor retardation in this slow-progressing disease.

I Background of the study

Aspartylglucosaminuria (AGU) is a lysosomal storage disorder that is characterized by progressive mental retardation. AGU is caused by mutations in the gene encoding for aspartylglucosaminidase (AGA), a lysosomal enzyme that participates in one of the final steps in the degradation of cellular glycoproteins. In the absence of AGA activity, glycoasparagines (glyco-Asn) accumulate in the lysosomes of patient cells, and are abundantly detected also in the urine. AGU patients are born normal, but the symptoms manifest during early childhood as delays in the general development such as speech and motor skills (Arvio & Arvio 2002, Arvio and Mononen 2016). The diagnosis for AGU is usually done by analyzing urine oligosaccharides and confirmed by genetic analysis of the *AGA* gene. Upon magnetic resonance imaging (MRI), changes typical for AGU can be observed (e.g. thalamic changes in T2-based imaging, and delayed myelination; see Tokola et al. 2015).

AGU is a progressive disease, and there currently are no curative treatment options available for AGU. Previously, attempts for bone marrow transplantation (BMT) have been made, but the benefits of this treatment were very modest, as compared to the potential risks. In an AGU knockout mouse model, studies on BMT were also performed, and a reduction of glyco-Asn in the cells of the nervous system were observed, when the transplantation was performed early enough (3-week-old mice), and the donor mouse was a healthy wildtype, and not a carrier mouse (with 50% AGA activity). The genotype may also have affected the human trials when relatives were used as donors. Furthermore, gene therapy studies on AGU mice using adenoviruses injected into brain tissue or the ventricles have also been carried out, in addition to enzyme replacement studies, but none of these have advanced in clinical trials (Laine et al. 1999 & 2004; Virta et al. 2006).

AGU is enriched in the Finnish population, and owing to a founder effect, almost all of the Finnish AGU patients carry a specific mutation, designated as $AGU_{Fin\text{-}major}$, in at least one allele, and most patients are thus homozygous for this mutation that causes a replacement of two amino acids. The Cys163Ser amino acid exchange in the AGA enzyme is the pathogenic variant of the disease, whereas Arg161Gln is an associated, non-pathogenic variant (Ikonen et al. 1991).

AGA enzyme is synthesized as a single polypeptide chain that immediately after synthesis becomes processed into two subunits, called α and β. The amino-terminal Thr206 of the β-subunit is located in the catalytic center of the AGA enzyme, directly participating in the cleavage of glycoasparagines, and the processing into the subunits is thus a prerequisite for the AGA enzyme activity (Tikkanen et al. 1996, Saarela et al. 1998).

The processing of the AGA precursor occurs autocatalytically by an intramolecular rearrangement of the strained scissile peptide bond between Asp205 and Thr206 upon dimerization of two AGA precursor molecules, resulting in the final active $(\alpha\beta)_2$ hetero-tetrameric form. In order to become processed, the AGA precursor has to be correctly folded. Most AGU mutations, including $AGU_{Fin\text{-}major}$, cause a local folding defect and structural instability, preventing the autocatalytic cleavage into the subunits, and the AGA precursor remains as a single polypeptide (Banning et al. 2016). The active site of the $AGU_{Fin\text{-}major}$ mutant precursors is intact, and the mutant polypeptide is relatively stable, and even becomes transported into

lysosomes. However, due to the lack of processing into subunits, it does not show any AGA activity (Banning et al. 2016).

Pharmacological chaperones (PC) are usually small molecules that can bind to their target proteins and stabilize their structures. In the case of diseases that are caused by protein misfolding due to mutations, PC compounds facilitate stabilization and correct folding of the mutant enzymes. The concept of pharmacological chaperone therapy (PCT) has emerged in recent years, providing novel treatment options for e.g. monogenic lysosomal and peroxisomal diseases (Parenti et al. 2015). PCs thus can increase the enzyme activity in patient cells and help to clear the substrate accumulation, relieving the lysosomal pathology that causes the disease.

The group of Dr. Tikkanen has been able to identify potential PC compounds that result in a substantial increase of AGA enzyme activity in fibroblasts from AGU patients homozygous for $AGU_{Fin\text{-}major}$ mutation. One of these compounds is betaine or trimethylglycine (Fig. 1). We were able to reach about 30% of the AGA enzyme activity measured in control fibroblasts from a healthy person (Banning et al. 2016). Although the enzyme activity in AGU fibroblasts after betaine treatment does not reach the level of healthy persons, it is close to the activity that could be expected in cells of a carrier (50%), who would not exhibit any symptoms of the disease. Furthermore, it is known that even a lower degree of residual lysosomal enzyme activity can result in amelioration of disease symptoms in lysosomal storage disorders (Conzelmann & Sandhoff 1983). In accordance, we observed a clear improvement of lysosomal pathology upon treatment of AGU fibroblasts with betaine (Banning et al. 2016).

A direct link to this publication: http://www.nature.com/articles/srep37583

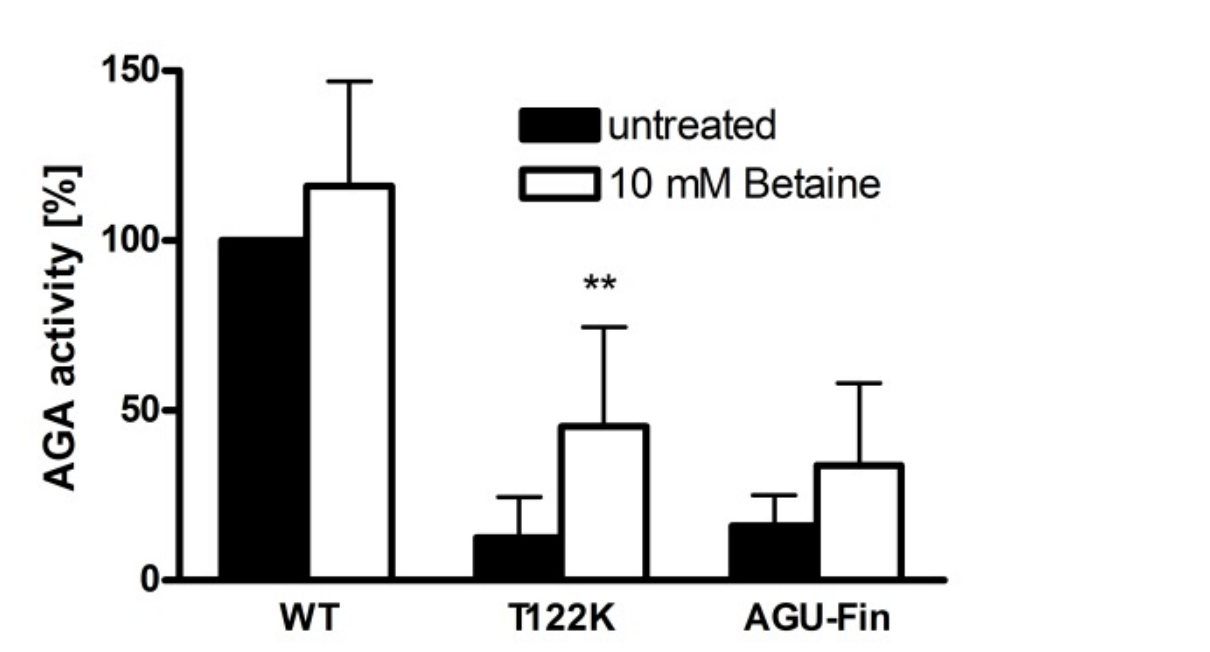

Fig. 1. Effect of betaine treatment on AGA enzyme activity in AGU patient fibroblasts treated in culture with betaine. Left graph shows the AGA enzyme activity (%) as compared to non-treated control cells. Right graph shows the relative fold-increase in AGA activity as compared to the respective untreated cells.

Betaine (trade name Cystadane) functions as a chaperone for its target molecules, and Cystadane has been approved for clinical use in homocystinuria. It is tested in a phase 3 clinical trial for the peroxisomal Zellweger spectrum disorder (ZSD). In the case of ZSD, betaine has been demonstrated to improve peroxisomal function in patient cells that carry a Gly834Asp-mutation in the gene encoding for the PEX1 protein (Zhang et al. 2010). PEX1 participates in the transport of peroxisomal proteins, and its malfunction results in peroxisomal aberrations. Gly834Asp-mutation results in misfolding of PEX1, which can be ameliorated by the PC function of betaine/Cystadane.

Please refer to the European Medicines Agency (EMA) pages about Cystadane for further details:

https://www.ema.europa.eu/documents/product-information/cystadane-epar-product-information_en.pdf

II Study Design, Objectives, Methods and Outline

The objectives of this study are to evaluate the safety and efficacy of Cystadane (anhydrous betaine) in the treatment of aspartylglucosaminuria. The study will be carried out as an interventional study with a maximum of 25 AGU patients. It is not possible to test the substance in the existing AGU animal model, as the two existing AGU knockout mouse models are based on either complete gene ablation or mimic an AGU mutation with a large genomic deletion of the AGA gene. Thus, both animal models are incompatible with our PCT approach, as no enzyme precursor is synthesized at all. The study substance, Cystadane, is in clinical use for homocystinuria, and it is actively studied in a phase 3 clinical study in peroxisomal diseases. Thus, abundant safety information and data for dosing in pediatric patients are available.

Cystadane treatment in AGU may prevent or slow down the progression of the disease. The study will be continued for 4 years, as long as results obtained during the first year are promising, and no major adverse effects appear. However, AGU is a slowly progressing disease, and a short intervention time may not be sufficient to prove efficacy.

Patient selection for the study

A prerequisite for the effect of the treatment to be tested in this study is that the patient cells exhibit the unprocessed precursor form of the AGA polypeptide, which will, with the aid of the chaperone function of betaine, be processed into the active enzymatic form. In our laboratory studies, betaine has proven efficacy (= increased AGA enzyme activity) in fibroblasts of patients who carry the $AGU_{Fin\text{-}major}$ or the Thr122Lys variant. Betaine shows no effect on the enzyme activity in the cells of patients who exhibit the $AGU_{Fin\text{-}minor}$ mutation. Thus, only patients who carry the $AGU_{Fin\text{-}major}$ mutation in both alleles will be recruited into our study. Because AGU is a progressive disease, we plan to recruit a maximum of 25 patients that are not older than 17 years, so that the benefits of the treatment can be measured. In older patients, assessment of efficacy may be difficult due to the advanced developmental regression and severe handicap. Patient sex is not relevant for the study. Patients who have undergone a bone marrow transplantation are not eligible for the study. The study will be carried out as an open-label study due to the small number of eligible patients, as blinding and division into two groups would not result in reliable data due to the small size of the groups.

Our group is actively working together with the patient organization (Suomen AGU r.y.) to identify suitable patients. At study begin, 21 AGU patients aged 7 to 15 years will be recruited, and further 4 patients (age 3 to 17 years) can be recruited for the final 3 years, should the results of the first year be promising. In addition, age- and sex-matched controls will be imaged for the MRI brain imaging studies.

Before the treatment begin, urinary glyco-Asn and AGA activity in the serum of the patients will be determined. In addition, MRI imaging of the brain, neuropsychological tests (WISC-IV), as well as a visit at the child neurologists, including family anamnesis, will be carried out. The parents will fill out questionnaires concerning adaptive skills and quality of life.

Cystadane is a medicine in powder form, and it can be orally administrated twice a day, mixed in food or beverage. The efficacy of the treatment will be measured according to the above measures that will be carried out during the visits at the child neurologist, with specific intervals. The study does not involve a placebo group, as there are not enough of suitable AGU patients for two study groups. Placebo will be replaced by a treatment break of 3 months (washout period) after 12 months of treatment, and after the 3-month break, urinary glyco-Asn and serum AGA activity will be measured to determine the reversible effects related to the treatment. We expect that due to the treatment break, the reduction in urinary glyco-Asn and the possible increase in AGA activity that were caused by the treatment will revert close to the level observed at study begin. If the preliminary data from the biochemical analysis are promising and no severe adverse effects are observed, the study will continue for a period of 4 years in total, so that reliable data for the disease progression can be observed.

The detailed description of the study design (with time points, possible adverse effects, methodology, and statistical plan) as well as the references are found in the appendix “Technical description”.

Currently, there are no treatment options for AGU that would aim at preventing the disease progression. The current study is thus a unique opportunity to test an easy-to-use, generally well-tolerated and non-invasive treatment for AGU. In cell culture experiments, use of betaine improves the AGA enzyme activity in AGU patient cells even close to a level in AGU carriers. Typically of lysosomal diseases, even a minor increase in the enzyme activity in patient cells may show a dramatic effect on the disease phenotype (threshold effect). Therefore, it is plausible that the chaperone treatment described in this study may slow down or even prevent the disease progression in AGU.

Tasks of the members of the clinical study group

Name	Contact	Role in the study
Minna Laine, MD, child neurologist	Department of Child Neurology, University of Helsinki and Helsinki University Hospital, P.O. Box 347, FI-00029 Helsinki, Finland	Sponsor, medical doctor<br><br>Responsible for the clinical aspects & child neurologist visits of the patients
Ritva Tikkanen, PhD, professor of biochemistry and molecular biology	Institute of Biochemistry, Medical Faculty, University of Giessen, Friedrichstrasse 24, D-35392 Giessen, Germany	Sponsor, responsible for the biochemical analyses of patient samples (urinary glyco-Asn, AGA-activity)
Taina Autti, MD, professor of radiology	HUS Medical Imaging Center, Radiology, University of Helsinki and Helsinki University Hospital, P.O. Box 340, FI-00029 Helsinki, Finland	MRI studies
Anna Tokola, MD, radiologist	HUS Medical Imaging Center, Radiology, University of Helsinki and Helsinki University Hospital, P.O. Box 340, FI-00029 Helsinki, Finland	MRI studies
Päivi Helenius, PhD, neuropsychologist	Department of Child Neurology, University of Helsinki and Helsinki University Hospital, P.O. Box 347, FI-00029 Helsinki, Finland	Neuropsychological testing, quality of life questionnaires, adaptive skills questionnaire
Elina Harjunen, psychologist		Neuropsychological testing, quality of life questionnaires, adaptive skills questionnaire

TECHNICAL DESCRIPTION OF THE STUDY

Persons Involved in the Study

Minna Laine, MD, PhD, child neurologist. PI for the clinical studies, responsible for the clinical care of the patients and compliance to the study protocol. She will carry out the child neurologist's examinations of the study patients, and coordinates the clinical part of the study. She is also responsible for the data protection, and collection and storage of patient-specific clinical data.

Ritva Tikkanen, PhD, Prof. of Biochemistry and Molecular Biology. PI for the scientific part of the studies. She will be responsible for supervising and analyzing the biochemical assays in patient samples (esp. urine glyco-Asn and serum AGA activity).

ML and RT are responsible for the reports to authorities, interpretation and publication of the study data.

Taina Autti, MD, Prof. of Radiology. Will be responsible for the MRI imaging studies and data analysis thereof.

Anna Tokola, MD, Radiologists. Will carry out and interpret the MRI imaging studies under the supervision of T. Autti.

Ulrika Roine, MD, M.Sc. (Tech.) and Timo Roine M.Sc. (Tech.). Will retrospectively perform tractography and tract-based spatial statistics to analyze brain white matter microstructure, and brain connectivity network analyses on the MRI imaging data**.**

Päivi Helenius, PhD, Neuropsychologist. Will design, carry out and interpret the neuropsychological evaluation, and adaptive skills and quality of life questionnaires. Supervises Elina Harjunen in her work.

Elina Harjunen, psychologist. Will perform most of the neuropsychological testing and analyzes the quality of life questionnaires under the supervision of P. Helenius.

Antje Banning, PhD, Senior Scientist. Will be responsible for the measurement and analysis of the serum AGA activity and urine creatinine values, as well as for the normalization of the urine glycoasparagine data.

Günter Lochnit, PhD, Professor of Biochemistry. Will be responsible for the quantitative measurement of urine glycoasparagines by mass spectrometry.

Helsinki University Hospital is one of the main hospitals in Finland, fully equipped for all parts of the clinical study. Prof. Tikkanen (Univ. of Giessen, DE) is the head of a research laboratory, and the methods for the measurement of AGA enzyme activity are established in her group. Urine glycoasparagine measurement is established in the Institute of Biochemistry (Prof. Lochnit). In addition to the persons mentioned above, further clinical personnel such as anesthetists, nurses etc., and technical personnel will be involved in the study as necessary.

STUDY DESIGN

Study outline

The clinical trial will be performed as a single-center, open label study, without randomization or placebo control. Due to the limited number of eligible patients, randomization and division of the cohort into separate groups is not feasible. Furthermore, as no alternative treatment is available and the disease is severe and progressive, placebo control should be considered unethical.

The study will be conducted at a single center at the University Hospital of Helsinki, Finland; mainly at the Peijas Hospital Unit in Vantaa. The study will enroll 21-25 patients who have been diagnosed with aspartylglucosaminuria. The patients will be between 3 and 17 years of age, and exhibit the $AGU_{Fin\text{-}major}$ mutation in a homozygous form. The patients will receive a codified number that will be used to track all the samples and biochemical data obtained during the study. A database containing patient IDs and codified numbers will be established in University Hospital of Helsinki, and is kept by Dr. Minna Laine.

The study period will be 48 months (4 years), divided into 2 study periods: exploratory phase of maximum 12 months, followed by a treatment interruption of 3 months, and a further treatment period of 33 months. A substantially shorter intervention time might not be sufficient to show the clinical benefit in terms of structural changes of the brain and developmental measures. However, further patients can be recruited during the study months 1-15. In this case, the patients will be included in the study for about 33-36 months total.

Blood and urine samples will be obtained on the day of the visit at the child neurologists at the clinical laboratory of the Peijas Hospital, labeled with the patient code numbers, and the tubes will be kept at -80°C freezer in the laboratory unit. The samples are later transferred to University of Giessen, Germany, for biochemical analysis (urine glyco-Asn, serum AGA activity). Separate samples (plasma) will be obtained for the safety test (plasma methionine), and immediately sent to HUSlab (an accredited clinical laboratory of the Helsinki University Hospital) for the measurement. For the biochemical analysis performed in Giessen, the codified patient numbers will be used, whereas the safety tests will be performed using the patient identification data (name and social security number).

Analysis of endpoints, and study timepoints

Prior to the start of the Cystadane treatment, the patients will be assessed by a child neurologist (Dr. M. Laine), and a neuropsychologist (Dr. Päivi Helenius or a member of her clinical team). In addition, brain MRI will be performed by Dr. T. Autti and her team. Blood and urine samples will be obtained for the analysis of urine glycoasparagines, serum AGA activity and plasma methionine.

The primary endpoint to measure the therapeutic efficacy is the amount of glycoasparagines secreted in the urine, normalized to urine creatinine. Secondary endpoints are the AGA activity in serum, neuropsychological evaluation and brain MRI scans. Plasma methionine levels are measured purely as safety test. These endpoint measurements are elucidated below in more detail. (See Table 1 for the schedule summary.)

Before starting the administration of Cystadane, urine glycoasparagines and AGA activity in patient serum are determined quantitatively in each patient, which will be used as a reference value for therapy success in both phases of the study. Furthermore, the concentration of methionine in the blood plasma will be measured. This is a safety measure, and the aim is to keep the Met plasma concentration below 700 umol/l throughout the treatment. Higher concentrations are acceptable if no symptoms pointing to drug-related adverse side effects are present.

The above measurements are performed in a standardized clinical laboratory (methionine: Huslab) or in the laboratory of Dr. Tikkanen with experience in the methods (AGA activity measurement, which is not available as a standard clinical test). Urine glycoasparagines will be measured by means of mass spectrometry by Dr. G. Lochnit in the Institute of Biochemistry in Giessen, as this is also not available as a standard clinical test. In addition, MRI scan of brains, a neuropsychological evaluation, child neurologist's examination and parent interview will be performed. Parents are also asked to fill in a modified "quality of life" questionnaire and an adaptive skills questionnaire.

After 3 months, the patients will again visit the child neurologist, and the blood and urine tests will be performed to monitor the therapeutic effect (urine glycoasparagines, serum AGA activity) and safety (methionine conc.).

6 months after initiation of the trial, the measurements will again be performed as after 3 months intervention. Child neurologist's examination will be performed at time points 0 months, 3 months, 6 months, 12 and 15 months, 24 months, and once a year after that. At the same time, a urine sample for glycoasparagine and a serum sample for AGA activity will be obtained at all time points.

Due to their young age (3-7 years), some patients will need anesthesia for the MRI scan, at least in the very beginning of the trial. Also older patients may need anesthesia due to fear and/or restlessness. The MRI will be performed once a year (0, 12, 24, 36 and 48 months), if possible without anesthesia, otherwise every other year. Anesthesia will be performed by an experienced child anesthetist. Neuropsychological evaluation and quality of life questionnaires will be performed at time points 0, 12, 24, 36 and 48 months after initiation of the treatment.

Table 1. Study timepoints.

Endpoint/ Time point	Urine glyco-Asn	Serum AGA activity	Plasma Met	Child Neurologist	Neuropsychological evaluation	Brain MRI
0 months	X	X	X	X	X	X
3 months	X	X	X	X		
6 months	X	X	X	X		
12 months	X	X	-	X	X	X
15 months	X	X	-	X		

**24 months**	**X**	**X**	**X**	**X**	**X**	**X**
**36 months**	**X**	**X**	**-**	**X**	**X**	**X**
**48 months**	**X**	**X**	**-**	**X**	**X**	**X**

Patient Inclusion and exclusion criteria

Inclusion criteria

- Patients below the age of 17 years, who have aspartylglucosaminuria
- Preferably homozygous for the Finnish major AGU mutation, $AGU_{Fin\text{-}major}$
- Written informed consent of the parents
- No known hypersensitivity or allergy against betaine
- Compliance of the patient

Exclusion criteria

- Age above 17 years at study begin
- Patients who do not carry at least one allele of the Finnish major mutation
- Known adverse reactions against betaine or components of Cystadane
- Known history of cerebral oedema
- Participating in other interventional clinical studies
- Patients who have undergone bone marrow transplantation
- Co-medication which would interfere with administration of betaine/Cystadane

Ethical Issues

The required statements of the local ethics committee will be obtained before the study begin and upon any possible major amendments. A thorough discussion with all participating families will be carried out individually, explaining the study rationale, design and risks etc. A written informed consent form will be signed by all participant families. The participants have the right to step down and cancel their participation at any time point.

The study population represents a specific vulnerable population, as all the patients are minors (below 18 years of age) who are mentally handicapped and thus not capable of personally giving an informed consent. Special care will be taken upon patient inclusion to take this fact into consideration. The consent form will be signed by a parent or other person that legally represents the patient.

The study will strictly adhere to ICH-GCP and/or local GCP regulations and all laws, rules, guidelines and regulations applicable to the Study. The study has been registered in a clinical study registry (EudraCT registration number: 2017-000645-48). The country of regulatory guidelines is Finland, and the clinical study application will be thus submitted to Fimea.

Treatment regime and schedule for clinical assessments

Cystadane is already in clinical use for homocystinuria. Therefore, the risk and safety data obtained in the previous studies will be utilized here for initial dosing. Cystadane powder (obtained from Orphan Europe) will be administrated orally as a suspension with a suitable food/drink such as yoghurt or juice. A measuring spoon will be provided for the correct dose.

Study period 1 (exploratory phase), from 0 to 6 - 12 months

The purpose of the initial exploratory phase is to obtain information about the safety of Cystadane (indicated by absence of serious adverse effects), and to assess the natural variation in the biochemical parameters (AGA activity, urine glycoasparagines) within the patient cohort prior to and during the initial treatment period of 6-12 months. We will use the data from 3, 6 and possibly 12 months after treatment start to define the potential response range and to obtain quantitative data for deciding how to assess the efficacy. In addition, within and after this initial phase, the dose may be adjusted. Figure 1 shows a summary of the trial phases and time points.

Figure 1. Summary of study phases and time points.

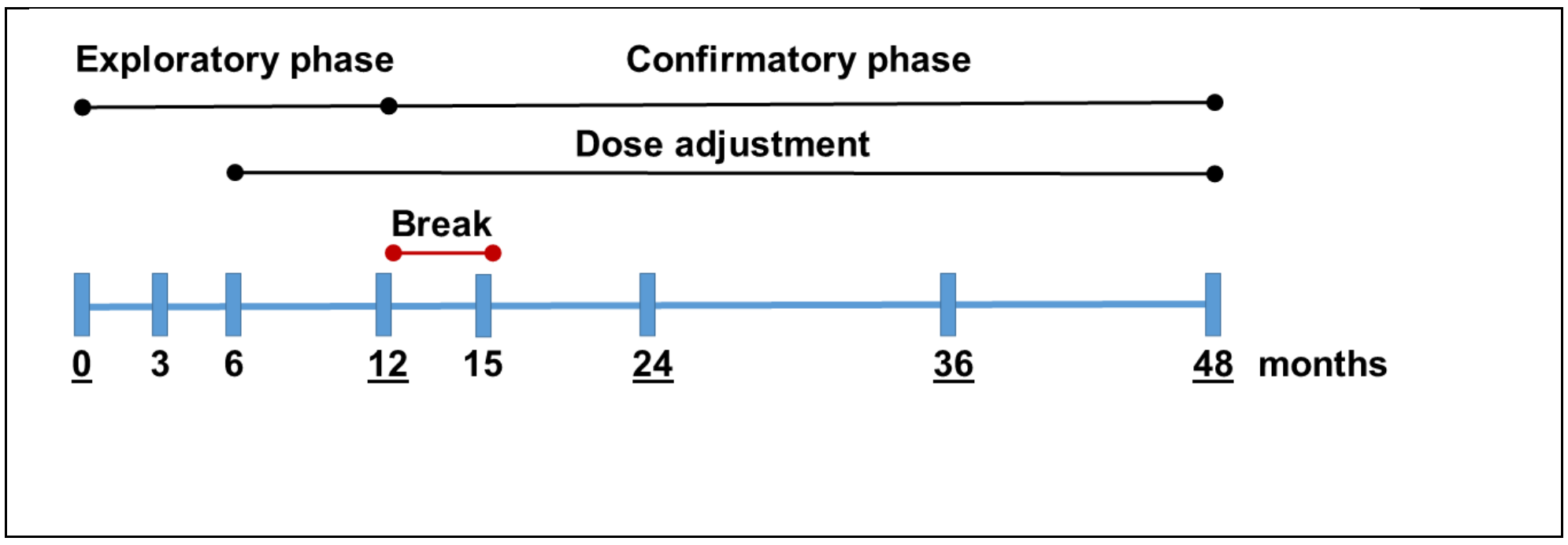

During the first 6-9 months of period 1, Cystadane will be administrated in all patients at 100 mg/kg/day, divided into two equal doses. Maximum dose will be 6 g/day, divided into 2 daily doses. After this period, dose escalation will be performed (up to 200 mg/kg/day or 12 g/day) in order to assess the maximum possible response.

After 6 months and 12 months of treatment, the biochemical data will be analyzed in order to assess the natural variation range of biochemical parameters within the cohort (0 months compared to 6 months or 12 months), and the decision about the dose escalation for the second phase of the study will be made, based on the observed response (changes in the biochemical parameters). Since the cohort consist of pediatric patients whose weight will considerably change during the study, a dose adjustment will be necessary. Furthermore, the initial maximum dose of 6 g/day may not be sufficient to induce a treatment response in the

older/heavier patients. The current recommended dose for Cystadane is up to 200 mg/kg/day or 12 g/day. This amount can even be exceeded, if required.

After the 12 months of treatment, there will be a 3-month break in the treatment. The purpose of this break is to see if the urine glycoasparagines start to rise and the serum AGA activity declines upon interruption of the intervention. This treatment break is used to replace the placebo treatment arm. Latest after the treatment break, the patients will move into period 2 (confirmatory phase with possible further dose adjustments) of the study.

Study period 2 (confirmatory phase with dose escalation), from 12-15 months onwards, until 48 months

The second period of the study will be started with a dose equal to the one that was administrated before the break. The first further dose escalation can be done after 1 month of restarting the study drug. Based on the observations from the study period 1 and patient parameters (weight, response during the phase 1), the individual dose will be further escalated for each patient to obtain a maximum response. The maximum dose will be 200 mg/kg/day, divided into two portions. This amount can even be exceeded if required. Should signs of adverse effects be observed upon dose escalation, the patient will return to previous dose until symptoms disappear, after which the dose will again be increased. In case further adverse effects appear upon dose escalation, the patient will remain until the end of the study at the dose level that was well tolerated.

Dose adjustment for the patients will be performed as follows: dose will be increased in increments of e.g. 30-50 mg/kg/day every 3 months, and the response will be measured at 24 and 36 months (judged as increase in serum AGA activity at 24 months or later, and reduction in urine glycoasparagines at 24 months or later), until dose reaches the maximum dose of 200 mg/kg/day, whichever happens first. If the response proves insufficient at 24 months, this amount can even be exceeded if required.

The treatment according to this dosing regime will continue for until 48 months from the start of the study. For the patients possibly recruited later into the study, the treatment may be continued for 33-36 months only.

METHODS FOR EFFICACY PARAMETERS

Primary Efficacy Endpoint: Urine Glycoasparagines

Urine glycoasparagines by HPLC/ESI-MS

All urine samples are obtained, if possible, as a morning urine. To exclude variations in the degree of urine concentration, glycoasparagine values will be normalized to creatinine. Urine glycoasparagines will be measured using quantitative high-performance liquid chromatography/electrospray mass spectrometry (HPLC/ESI-MS, see Table 2). Diluted urine samples (20 µl of 1/80 dilution in water) is injected into HPLC column, followed by MS analysis. Since N-Acetylglucosamine-asparagine (GlcNAc-Asn) is the main accumulation product, quantitative measurement will be performed on the peak containing this product. GlcNAc-Asn standards will be used to allow a quantitative analysis of the peaks, based on the peak area.

Triplicate measurements are performed and data are expressed as pmol GlcNAc-Asn per µl of urine. Typical running conditions are presented in Table 2. The method and conditions may be modified and improved, if necessary.

Table 2. HPLC/MS analysis of urine samples.

Column<br>Particle Size, Length × inner diameter	Hypercarb<br>3µm, 100x2.1 mm
Separation mode descriptions	analytical
Mobile Phase	Solvent A: 0.1% formic acid in water
Elution conditions	0 -10 min 100% Solvent A
Flow rate	200 µl/min
Injection	20 µl
Column temperature	30°C
Pressure	60-80 bar
HPLC system	Alliance HT Waters 2790 with Waters micromass ZQ 4000<br>DAD 210-400 nm<br>Detection: +ESI mass traces<br>204 (HexNAc+)<br>336 (GlcNAc-Asn H+)<br>358 (GlcNAc-Asn Na+)
Sample and sample preparation	Urine diluted 1 to 80 with water

Normalization of the data to creatinine

Urine glycoasparagine concentration will be normalized to creatinine to avoid variations caused by the diluteness of the urine sample. Urine samples are stored at -80°C until analysis. Samples are thawed on ice and shaken 10 times prior to use. Urine samples are diluted tenfold in H_2O (100 µl of urine + 900 µl of H_2O). For creatinine measurement the “Creatinine (urinary) Colorimetric Assay Kit” (Cayman Chemical) is used according to the manufacturers protocol. In a clear 96-well plate, 15 µl of the diluted sample is mixed with 150 µl of Alkaline Picrate Solution. The plate is covered and incubated on a shaker for 10 minutes at room temperature. Then the absorbance is read at 490 nm (initial absorbance). Thereafter, 5 µl of acid solution is added to each well. The covered plate is incubated on a shaker for another 20 minutes at room temperature. The absorbance is read again at 490 nm (final absorbance). A creatinine standard curve ranging from 2-20 mg/dl is prepared according to the manufacturer’s protocol. All samples and standards are measured in triplicate.

For calculation, the average final absorbance is subtracted from the average initial absorbance to receive the corrected absorbance. According to the standard curve, values are expressed as mg/dl. The intra-assay coefficient of variation (CV) is 2.8%. The inter-assay coefficient of variation is 1.3%.

The data obtained from the quantitative mass spectrometry analysis are normalized using the obtained creatinine values. The final output of the normalized urine glyco-Asn analysis will be given as mg Asn-GlcNAc per g creatinine ± standard deviation.

Secondary Endpoints

Serum aspartyglucosaminidase activity

Serum AGA enzyme activity will be measured using a fluorometric enzyme activity assay described in Banning et al. 2016, with modifications. 20 µl or patient serum will be used for the assay, which will be carried out in triplicates.

Serum samples are stored at -80°C until analysis. Samples are thawed on ice and vortexed prior to use. Per sample, three 0.5 ml tubes are prepared containing 20 µl of serum and 20 µl of 50 µM Asp-AMC substrate solution (L-Aspartic acid β-(7-amido-4-methylcoumarin; Santa Cruz sc-211699). The substrate is initially dissolved in 1 M HCl at 1 mM concentration. Out of this stock solution, the 50 µM substrate solution is prepared in McIlvains phosphate-citrate buffer pH 6.5. Triplicate blank samples are prepared containing 20 µl of isotonic NaCl solution and 20 µl of the substrate Asp-AMC solution. AMC (7-Amino-4-methylcoumarin, Sigma Aldrich A9891) is used as standard and dissolved in isotonic NaCl solution. For a standard curve, 5 tubes are prepared containing 20 µl of standard, ranging from 0.75 - 15 pmol AMC/µl, and 20 µl of Asp-AMC substrate solution. As an internal control, 10 µl of serum of a healthy donor is mixed with 10 µl of isotonic NaCl solution and 20 µl of Asp-AMC substrate solution. All tubes are vortexed and incubated at 37°C for 24 h. After 24 h, all tubes are centrifuged for 10 sec to collect condensed fluid at the bottom of the tube. Reactions are stopped by addition of 200 µl of McIlvain's phosphate-citrate buffer pH 4.5. All tubes are vortexed. 200 µl out of each tube are pipetted into a black 96-well plate. Plates are measured immediately (excitation: 355 nm, emission: 460 nm) with a Tecan Infinite m200 plate reader. For calculation of the enzyme activity, the mean blank fluorescence is subtracted. According to the slope of the standard curve, values are expressed as mU/L (1 U = 1 µmol/min). Data are shown as mean ± standard deviation. The intra-assay coefficient of variation (CV) is 2.7% for samples of healthy donors and 20.3% for patient samples.

Samples that are hemolytic (red in color) will be excluded from the final data analysis since the presence of hemoglobin in the sample inhibits the AGA enzyme activity and no reliable data can be obtained. The method and conditions may be modified and improved, if necessary.

The final output of the serum AGA activity analysis will be given as mU/L ± standard deviation.

Brain Imaging studies

Magnetic Resonance Imaging (MRI) studies will be performed in the patients, as well as in age- and sex-matched healthy children. Conventional, structure-defining sequences will be obtained as follows: T1-weighted 3D series, Flair 3D series, T2-weighted axial series, SWI series and diffusion-weighted series. The imaging data will be obtained at 0 months and appr. every 12 months from thereon in both controls and patients. The data at 0 months are compared with the control probands, whereas the later time points will be analyzed by comparing the data with the 0 months of the same patient. Previous own published data are available about the typical brain changes observed in AGU patients (Tokola et al. 2015), which will be useful for the analysis. Further analysis of the MRI data may be performed to dissect brain microstructural changes and brain connectivity networks, as feasible.

Neuropsychological studies

All children are tested with Wechsler's Intelligence Scale for Children IV (WISC-IV; Wechsler Intelligence Scale for Children – Fourth Edition. Wechsler D. 2004. San Antonio, TX: Harcourt Assessment). WISC-IV provides an overall measure of intelligence. It consists of four subscales: Verbal Comprehension Index (VCI), Perceptual Reasoning Index (PRI), Working Memory Index (WMI) and Processing Speed Index (PSI). Together, the four subscales provide Full Scale Intelligence Quotient (FSIQ). Verbal Comprehension includes core subtests Similarities (SI), Vocabulary (VO) and Comprehension (CO). The core subtests for Perceptual Reasoning are Block Design (BD), Picture Concepts (PC) and Matrix Reasoning (MR). Working Memory consists of Digit Span (DS) and Letter-Number Sequencing (LN). The core subtests for Processing Speed are Coding (CO) and Symbol Search (SS).

Longitudinal changes in the neuropsychological test scores (WISC-IV raw scores for each test) are analyzed using repeated measures ANOVAs with time as a within-subjects factor (before treatment, 1 year, 2 years, 3 years and 4 years). Separate ANOVAs are performed to determine the short-term (1 year), and long-term (2-4 years) effects of the intervention.

SAFETY PARAMETERS

The safety data will be summarized for all subjects within the study population and assessed as absence of serious adverse events (SAE).

Observed adverse events will be summarized as:

- All adverse effects (mild, moderate, severe)
- Serious adverse effects
- Events judged to be related/possibly related/unrelated to the intervention
- Events leading to discontinuation of the treatment

Any serious adverse effects will be adequately and immediately reported to authorities.

Safety will be biochemically assessed by measuring the methionine concentration in the blood plasma. Increased methionine levels and increased intracranial pressure have been reported to be a rare side effect of Cystadane in the patients with homocystinuria with cystathionine-beta-synthase gene defect. Thus, it is highly unlikely that these symptoms will be observed in AGU patients during the treatment. In case of such symptoms (vomiting and headache in the morning, vision problems or excess increase of plasma methionine conc.) the treatment will be discontinued until symptoms disappear. After that, the treatment will be started again, but if symptoms reoccur, the treatment will be stopped permanently, and the patient will be excluded from the study. Any history of prior intracranial pressure symptoms will be an exclusion criterion.

STATISTICAL ANALYSIS PLAN AND METHODOLOGY

Statistical Plan was prepared by: Dr. Jörn Pons-Kühnemann, Institute for Medical Informatics, Medical Statistics, University of Giessen, Rudolf-Buchheim-Str. 6, D-35392 Giessen, Germany

Statistical Methods and Data Analysis

In the planned interventional clinical trial on AGU patients, urine glycoasparagine levels (as primary endpoint) and AGA enzyme activity in the patient serum (as secondary endpoint) will be determined at different time points (0, 3, 6, 12, 15, 24, 36 and 48 months) after starting the intervention with Cystadane, as described in the clinical trial plan. The study is a longitudinal study with repeated measurements, where the observations are clustered within a subject. This means that the observations and their residuals are correlated and not independent. Due to the repeated measurement design, a linear mixed model will be applied to the data using SPSS Statistics for Windows Version 25 (IBM Corp. Released 2017. Armonk, NY: IBM Corp.). To control the covariance structure of the residuals for a single subject, the repeated covariance type AR(1), a first-order autoregressive structure with homogenous variances was chosen. To reach normal distribution of residuals, the data are ln-transformed. Estimated marginal means for the fitted models and 95% confidence intervals will be calculated. Treatment differences are described by estimated differences and their 95% confidence intervals. All means and confidence intervals are Bonferroni adjusted within the analysis for each outcome. Differences and boundaries of confidence interval are anti-ln-transformed for the presentation of the results.

The planned study is divided into two phases. The data obtained during the first, exploratory phase (0 to up to 12 months) will be preliminarily analyzed and used to estimate the potential degree of change in the urine glyco-Asn and serum AGA activity required for the demonstration of statistically significant efficacy. In the second phase of the study, with possible dose escalation and adjustment, the data required for the demonstration of efficacy are obtained and calculated using the statistical model above.

Dropouts and Missing Data

The study population consist of patients with verified diagnosis for aspartylglucosaminuria, who are homozygous for the major Finnish AGU mutation $AGU_{Fin-major}$ that results in the Cys163Ser substitution in the AGA enzyme. Patients between 3 and 17 years of age are eligible to participate. Patients who have undergone an allogenic bone marrow transplantation are excluded from the study (not enrolled).

If a subject is excluded from the study due to safety issues or withdrawal from the study after the treatment start, all sample data obtained until this time point can be included in the final study data, or considered as dropouts. Upon patient family request, all unused samples collected so far will be destroyed.

If a clear reason is identified why a specific patient sample needs to be excluded from the data analysis (e.g. hemolysis of the serum sample or other sample quality issues), the data concerning this specific sample will be excluded, whereas all other data points concerning the same patient can be included.

Determination of Sample Size

AGU is a rare disease with main representation in Finland. With the help of the Finnish patient organization Suomen AGU r.y. and clinicians, we have identified all known AGU patients in Finland that are between 3 and 17 years of age and exhibit the suitable genotype (homozygous for the major Finnish AGU mutation $AGU_{Fin\text{-}major}$). Thus, our sample size is limited by the number of known cases.

Summary of the Subjects

Numbers for subject disposition will be provided for the following categories:

- Number of subjects included in the study upon baseline visit
- Number of subjects withdrawn before treatment completion
- Number of subjects recruited later than within 6 months of recruitment of the first patient
- Number of subject excluded due to safety issues
- Number of subjects who have completed the study

Further data to be collected include the age, gender and *AGA* genotype, as well as standard medical data such as patient weight and height. Relevant prior or concomitant medication will be recorded. Prior medication refers to medication that was started and completed prior to baseline, concomitant medication refers to medication that was started before baseline and continued during the study. Further medical history will eventually be collected, when relevant to the study.

Efficacy Analyses

Assessment of efficacy will be based mainly on the primary efficacy variable data (urine glycoasparagine reduction) and the secondary efficacy variable (serum AGA activity).

Primary Efficacy Variable: Urine Glycoasparagines

The primary endpoint is a decrease in the urine glycoasparagine amounts (as measured by mass spectrometry) within the treatment period. The urine glycoasparagine amount during treatment will be compared with the baseline value (0 months, pre-treatment) of the respective patient. Values are represented as mg Asn-GlcNAc per g creatinine ± standard deviation. The changes in the values can be expressed either as absolute values, or as relative values. This is necessary since the absolute values within the patient cohort are likely to be heterogeneous even in the baseline. The absolute values are plotted as a function of study time point for each patient, and the change in urine Asn-GlcNAc is calculated.

Efficacy will be determined as a statistically significant reduction in the urine glycoasparagine amounts (given as mg Asn-GlcNAc per g creatinine) as compared to the baseline value (at 0 months).

Secondary Efficacy Variable

Serum AGA activity

The secondary endpoint is the increase in serum AGA enzyme activity (measured using the enzyme activity assay) within the treatment period. The serum AGA activity during treatment will be compared with the baseline value (0 months, pre-treatment) of the respective patient. Values are represented as mU/l ± standard deviation. The changes in the values can be expressed both as absolute values or as relative values. This may be necessary since the absolute values within the patient cohort are likely to be heterogeneous even in the baseline. The absolute values are plotted as a function of study time point for each patient, and the change in serum AGA activity is calculated.

Efficacy will be determined as a statistically significant increase in the serum AGA activity (given as mU/l AGA activity) as compared to the baseline value (at 0 months).

Further Secondary Endpoints

Brain Imaging studies

Suitable statistical methods will be used for analyzing the MRI data. The data will be considered as confirmatory information that is not necessarily required for efficacy assessment. However, these data will be important in judging if the study drug exhibits a positive effect on the brain structure, which would imply that the drug is able to reach the brain.

Neuropsychological studies

Longitudinal changes in the neuropsychological test scores (WISC-IV raw scores for each test, ABAS-3) are analyzed using repeated measures ANOVAs with time as a within-subjects factor (before treatment, 1 year, 2 years, 3 years and 4 years). Separate ANOVAs are performed to determine the short-term (1 year), and long-term (2-4 years) effects of the intervention.

The data will be considered as confirmatory information that is not necessarily required for efficacy assessment. However, these data will be important in judging if the study drug exhibits a positive effect on behavior and mental/physical capabilities, which would imply that the drug is able to reach the brain.

Bibliography

Arvio P, Arvio M (2002) Progressive nature of aspartylglucosaminuria. Acta Paediatr. 2002;91(3):255-7.

Arvio M, Mononen I (2016) Aspartylglycosaminuria: a review. Orphanet J Rare Dis. 11(1):162.

Banning A, Gülec C, Rouvinen J, Gray SJ, Tikkanen R. (2016) Identification of Small Molecule Compounds for Pharmacological Chaperone Therapy of Aspartylglucosaminuria. Sci. Rep. 6, 37583; doi: 10.1038/srep37583

Conzelmann E, Sandhoff K (1983) Partial enzyme deficiencies: residual activities and the development of neurological disorders. Dev Neurosci. *6*:58–71.

Laine, M., Ahtiainen, L., Rapola, J., Richter, J., and Jalanko, A. (2004). Bone marrow transplantation in young aspartylglucosaminuria mice: improved clearance of lysosomal storage in brain by using wild type as compared to heterozygote donors. Bone Marrow Transplant 34, 1001-1003.

Laine, M., Richter, J., Fahlman, C., Rapola, J., Renlund, M., Peltonen, L., Karlsson, S., and Jalanko, A. (1999). Correction of peripheral lysosomal accumulation in mice with aspartylglucosaminuria by bone marrow transplantation. Exp Hematol 27, 1467-1474.

Ikonen E, Baumann M, Gron K, Syvanen AC, Enomaa N, Halila R, Aula P, Peltonen L (1991). Aspartylglucosaminuria: cDNA encoding human aspartylglucosaminidase and the missense mutation causing the disease. EMBO J *10*, 51-58.

Parenti G, Andria G, Ballabio A (2015). Lysosomal storage diseases: from pathophysiology to therapy. Annu Rev Med *66*, 471-486.

Saarela, J., Laine, M., Tikkanen, R., Oinonen, C., Jalanko, A., Rouvinen, J., and Peltonen, L. (1998). Activation and oligomerization of aspartylglucosaminidase. J Biol Chem *273*, 25320-25328.

Tikkanen, R., Riikonen, A., Oinonen, C., Rouvinen, R., and Peltonen, L. (1996). Functional analyses of active site residues of human lysosomal aspartylglucosaminidase: implications for catalytic mechanism and autocatalytic activation. EMBO J *15*, 2954-2960.

Tokola A, Åberg LE, Autti TH (2015) Brain MRI findings in aspartylglucosaminuria. J Neuroradiol. 42(6):345-57. doi: 10.1016/j.neurad.2015.03.003.

Virta, S., Rapola, J., Jalanko, A., and Laine, M. (2006). Use of nonviral promoters in adenovirus-mediated gene therapy: reduction of lysosomal storage in the aspartylglucosaminuria mouse. J Gene Med 8, 699-706.

Zhang R, Chen L, Jiralerspong S, Snowden A, Steinberg S, Braverman N (2010). Recovery of PEX1-Gly843Asp peroxisome dysfunction by small-molecule compounds. Proc Natl Acad Sci USA *107*, 5569-5574.

Annex 1: Clinical trial Application Form

REQUEST FOR AUTHORISATION OF A CLINICAL TRIAL ON A MEDICINAL PRODUCT FOR HUMAN USE TO THE COMPETENT AUTHORITIES AND FOR OPINION OF THE ETHICS COMMITTEES IN THE COMMUNITY

To be filled in by the applicant
The questions in this form for the request for authorisation from the Competent Authority are also relevant for the opinion from an Ethics Committee (it represents module 1 of the form for applying to an ethics committee) and can be used as part of that application. Please indicate the relevant purpose in a box below.

REQUEST FOR AUTHORISATION TO THE COMPETENT AUTHORITY: **Yes •**
REQUEST FOR OPINION OF THE ETHICS COMMITTEE: **No •**

A. TRIAL IDENTIFICATION

A.1	Member State in which the submission is being made:		**Finland - Fimea**
A.2	EudraCT number:		**2017-000645-48**
A.3	Full title of the trial:		
	**English**	**Open-label study to evaluate efficacy and safety of Cystadane for the treatment of aspartylglucosaminuria**	
	**Finnish**	**Cystadane aspartyyliglukosaminurian hoidossa: tehoa ja turvallisuutta koskeva tutkimus**	
A.3.1	Title of the trial for lay people, in easily understood, i.e. non-technical, language:		
	**English**	**Cystadane in the treatment of AGU**	
	**Finnish**	**Cystadane AGU-taudin hoidossa**	
A.3.2	Name or abbreviated title of the trial where available:		
	**English**	**Cystadane in the treatment of AGU**	
	**Finnish**	**Cystadane AGU-taudin hoidossa**	
A.4	Sponsor's protocol code number, version and date[1]:		
A.4.1	Sponsor's protocol code number:		**AGU-001**
A.4.2	Sponsor's protocol version:		**1**
A.4.3	Sponsor's protocol date:		**2017-04-19**
A.5	Additional international study identifiers (e.g. WHO, ISRCTN[2] , US NCT Number[3]) if available		
A.5.1	ISRCTN number:		
A.5.2	US NCT number:		
A.5.3	WHO Universal Trial Number (UTN):		
A.5.4	Other Identifier:		
A.6	Is this a resubmission?		**No •**
	If 'Yes', indicate the resubmission letter[4]:	**First Submission**	
A.7	Is the trial part of an agreed Paediatric Investigation Plan?		**No •**
A.8	EMA Decision number of Paediatric Investigation Plan:		

B. IDENTIFICATION OF THE SPONSOR RESPONSIBLE FOR THE REQUEST

**B.1**	**SPONSOR**	
B.1.1	Name of organisation:	**Minna Laine**
B.1.2	Name of the person to contact:	
B.1.2.1	Given name	**Minna**
B.1.2.2	Middle name	
B.1.2.3	Family name	**Laine**
B.1.3	Address:	
B.1.3.1	Street address	**Peijas hospital, Sairaalakatu 1/ P.O.Box 900**
B.1.3.2	Town/city	**Vantaa**
B.1.3.3	Post code	**00029 HUS**
B.1.3.4	Country	**Finland**
B.1.4	Telephone number:	
B.1.5	Fax number:	
B.1.6	E-mail:	**minna.laine@hus.fi**

**B.2**	**LEGAL REPRESENTATIVE[5] OF THE SPONSOR IN THE COMMUNITY FOR THE PURPOSE OF THIS TRIAL** (if different from the sponsor)
B.2.1	Name of organisation:
B.2.2	Name of person to contact:
B.2.2.1	Given name
B.2.2.2	Middle name
B.2.2.3	Family name
B.2.3	Address:
B.2.3.1	Street address
B.2.3.2	Town/city
B.2.3.3	Post code
B.2.3.4	Country
B.2.4	Telephone number:
B.2.5	Fax number:
B.2.6	E-mail:

**B.3**	**STATUS OF THE SPONSOR:**	
B.3.1	Commercial:	**No •**
B.3.2	Non commercial:	**Yes •**

**B.4**	**Source(s) of Monetary or Material Support for the clinical trial (repeat as necessary):**	
B.4.1	Name of organisation:	**Orphan Europe SARL**
B.4.2	Country:	**France**

**B.5**	**Contact point[6] designated by the sponsor for further information on the trial**	
B.5.1	Name of organisation:	**University of Giessen**
B.5.2	Functional name of contact point (e.g. "Clinical Trial Information Desk"):	**Prof. Dr. Ritva Tikkanen**
B.5.3	Address:	
B.5.3.1	Street address	**Friedrichstrasse 24**
B.5.3.2	Town/city	**Giessen**
B.5.3.3	Post code	**35392**
B.5.3.4	Country	**Germany**
B.5.4	Telephone number:	**49 641 9947 420**
B.5.5	Fax number:	
B.5.6	E-mail: (use a functional e-mail address rather than a personal one)	**ritva.tikkanen@biochemie.med.uni-giessen.de**

B.1	SPONSOR	
B.1.1	Name of organisation:	**Prof. Ritva Tikkanen**
B.1.2	Name of the person to contact:	
B.1.2.1	Given name	**Ritva**
B.1.2.2	Middle name	**Hannele**
B.1.2.3	Family name	**Tikkanen**
B.1.3	Address:	
B.1.3.1	Street address	**Friedrichstrasse 24**
B.1.3.2	Town/city	**Giessen**
B.1.3.3	Post code	**D-35392**
B.1.3.4	Country	**Germany**
B.1.4	Telephone number:	**49 641 9947 420**
B.1.5	Fax number:	
B.1.6	E-mail:	**ritva.tikkanen@biochemie.med.uni-giessen.de**

B.2	**LEGAL REPRESENTATIVE[5] OF THE SPONSOR IN THE COMMUNITY FOR THE PURPOSE OF THIS TRIAL** (if different from the sponsor)
B.2.1	Name of organisation:
B.2.2	Name of person to contact:
B.2.2.1	Given name
B.2.2.2	Middle name
B.2.2.3	Family name
B.2.3	Address:
B.2.3.1	Street address
B.2.3.2	Town/city
B.2.3.3	Post code
B.2.3.4	Country
B.2.4	Telephone number:
B.2.5	Fax number:
B.2.6	E-mail:

B.3	STATUS OF THE SPONSOR:	
B.3.1	Commercial:	**No •**
B.3.2	Non commercial:	**Yes •**

B.4	Source(s) of Monetary or Material Support for the clinical trial (repeat as necessary):	
B.4.1	Name of organisation:	**Orphan Europe SARL**
B.4.2	Country:	**France**

B.5	Contact point[6] designated by the sponsor for further information on the trial	
B.5.1	Name of organisation:	**Prof. Dr. Ritva Tikkanen**
B.5.2	Functional name of contact point (e.g. "Clinical Trial Information Desk"):	**Scientific Coordinator**
B.5.3	Address:	
B.5.3.1	Street address	**Friedrichstrasse 24**
B.5.3.2	Town/city	**Giessen**
B.5.3.3	Post code	**D-35392**
B.5.3.4	Country	**Germany**
B.5.4	Telephone number:	**49 641 9947 420**
B.5.5	Fax number:	
B.5.6	E-mail: (use a functional e-mail address rather than a personal one)	**ritva.tikkanen@biochemie.med.uni-giessen.de**

C. APPLICANT IDENTIFICATION, (please tick the appropriate box)

**C.1**	**REQUEST FOR THE COMPETENT AUTHORITY**		
C.1.1	Sponsor		**Yes •**
C.1.2	Legal representative of the sponsor		
C.1.3	Person or organisation authorised by the sponsor to make the application		
C.1.4	Complete the details of the applicant below even if they are provided elsewhere on the form:		
C.1.4.1	Name of Organisation:	**Minna Laine**	
C.1.4.2	Name of contact person:		
C.1.4.2.1	Given name	**Minna**	
C.1.4.2.2	Middle name		
C.1.4.2.3	Family name	**Laine**	
C.1.4.3	Address:		
C.1.4.3.1	Street address	**Peijas hospital, Sairaalakatu 1/ P.O.Box 900**	
C.1.4.3.2	Town/city	**Vantaa**	
C.1.4.3.3	Post code	**00029 HUS**	
C.1.4.3.4	Country	**Finland**	
C.1.4.4	Telephone number:		
C.1.4.5	Fax number:		
C.1.4.6	E-mail:	**minna.laine@hus.fi**	
C.1.5	Request to receive a copy of CTA data as XML:		
C.1.5.1	Do you want a copy of the CTA form data saved on EudraCT as an XML file?	**Yes •**	
C.1.5.1.1	If Yes provide the e-mail address(es) to which it should be sent (up to 5 addresses):<br>**ritva.tikkanen@biochemie.med.uni-giessen.de**<br>**minna.laine@hus.fi**		
C.1.5.1.2	Do you want to receive this via password protected link(s)[7]?	**No •**	
If you answer No to question C.1.5.1.2 the .xml file will be transmitted by less secure e-mail link(s)			

D. INFORMATION ON EACH IMP

Information on each 'bulk product' before trial-specific operations (blinding, trial specific packaging and labelling) should be provided in this section for each investigational medicinal product (IMP) being tested including each comparator and each placebo, if applicable. ***For placebo go directly to D.8****. If the trial is performed with several products use extra pages and give each product a sequential number in D.1.1. If the product is a combination product, information should be given for each active substance.*

**D.1**	**IMP IDENTIFICATION**	
Indicate which of the following is described below, then repeat as necessary for each of the numbered IMPs to be used in the trial (assign numbers from 1-n):		
D.1.1	This refers to the IMP number:	**PR1**
D.1.2	IMP being tested	**Yes •**
D.1.3	IMP used as a comparator	**No •**

**D.2**	**STATUS OF THE IMP**	
D.2.1	Has the IMP to be used in the trial a marketing authorisation?	**Yes •**
**If the IMP has a marketing authorisation in the Member State concerned by this application, but the trade name and marketing authorisation holder are not fixed in the protocol, go to section D.2.2.**		
D.2.1.1	If 'Yes', specify the product to be used in the clinical trial:	
D.2.1.1.1	Trade name	**Cystadane anhydrous**
D.2.1.1.1.1	EV Product Code (where applicable)	**EMEA/H/C/000678**
D.2.1.1.2	Name of the Marketing Authorisation Holder:	**Orphan Europe SARL**
D.2.1.1.3	Marketing Authorisation number (if Marketing Authorisation granted by a Member State):	**EU/1/06/379/001**
D.2.1.1.4	Is the IMP modified in relation to its Marketing Authorisation?	**No •**
D.2.1.1.4.1	If 'Yes', please specify:	
D.2.1.2	The country that granted the Marketing Authorisation	**European Union**
D.2.1.2.1	Is this the Member State concerned with this application?	**Not Answered •**

D.2.2	Situations where an IMP to be used in the CT has a Marketing Authorisation in the Member State concerned, but the protocol allows that any brand of the IMP with a Marketing Authorisation in that Member State be administered to the trial subjects and it is not possible to clearly identify the IMP(s) in advance of the trial start	
D.2.2.1	In the protocol, is treatment defined only by active substance?	**No •**
D.2.2.1.1	If 'Yes', give active substance in D.3.8 or D.3.9	
D.2.2.2	In the protocol, do treatment regimens allow different combinations of marketed products used according to local clinical practice at some or all investigator sites in the MS?	**No •**
D.2.2.2.1	If 'Yes', give active substance in D.3.8 or D.3.9	
D.2.2.3	The products to be administered as IMPs are defined as belonging to an ATC group[9]	**Yes •**
D.2.2.3.1	If 'Yes', give the ATC group of the applicable authorised codes in the ATC code field (level 3 or the level that can be defined) in D.3.3	
D.2.2.4	Other:	**No •**
D.2.2.4.1	If 'Yes', please specify:	

D.2.3	IMPD submitted:	
D.2.3.1	Full IMPD:	**Yes •**
D.2.3.2	Simplified IMPD:	**No •**
D.2.3.3	Summary of product characteristics (SmPC) only:	**No •**
D.2.4	Has the use of the IMP been previously authorised in a	**No •**

	clinical trial conducted by the sponsor in the Community?	
D.2.4.1	If 'Yes' specify which Member States:	
D.2.5	Has the IMP been designated in this indication as an orphan drug in the Community?	**Yes •**
D.2.5.1	If 'Yes', give the orphan drug designation number[10]**:**	**EU/3/01/045**

D.2.6	Has the IMP been the subject of scientific advice related to this clinical trial?	**No •**
D.2.6.1	If 'Yes' to D.2.6, please indicate source of advice and provide a copy in the CTA request:	
D.2.6.1.1	CHMP[11]?	**No •**
D.2.6.1.2	National Competent Authority?	**No •**

**D.3**	**DESCRIPTION OF THE IMP**	
D.3.1	Product name where applicable[12]:	
D.3.2	Product code where applicable[13]:	
D.3.3	ATC codes, if officially registered[14]:	**A16AA06**
D.3.4	Pharmaceutical form (use standard terms):	**Oral powder**
D.3.4.1	Is this a specific paediatric formulation?	**No •**
D.3.5	Maximum duration of treatment of a subject according to the protocol: **48 months**	
D.3.6	Dose allowed:	
D.3.6.1	For first trial only:	
	Specify per day or total	**Total •**
	Specify total dose (number and unit):	
	Route of administration (relevant to the first dose):	
D.3.6.2	For all trials	
	Specify per day or total	**Per day •**
	Specify total dose (number and unit):	**6 g gram(s)**
	Route of administration (relevant to the maximum dose):	**Oral use**
D.3.7	Routes of administration (use standard terms):	**Oral use**

D.3.8	Name of each active substance (INN or proposed INN if available):
D.3.9	Other available name for each active substance (provide all available):
D.3.9.1	CAS[15] number
D.3.9.2	Current sponsor code
D.3.9.3	Other descriptive name
D.3.9.4	EV Substance code
D.3.9.5	Full Molecular formula
D.3.9.6	Chemical/biological description of the Active Substance
D.3.10	Strength (specify all strengths to be used):
D.3.10.1	Concentration unit:
D.3.10.2	Concentration type ("exact number", "range", "more than" or "up to"):
D.3.10.3	Concentration (number).

D.3.11	Type of IMP	
Does the IMP contain an active substance:		
D.3.11.1	Of chemical origin?	**No •**
D.3.11.2	Of biological / biotechnological origin (other than Advanced Therapy IMP (ATIMP)?	**Yes •**
Is this a:		
D.3.11.3	Advanced Therapy IMP (ATIMP)?	**No •**
D.3.11.3.1	Somatic cell therapy medicinal product[16]?	**No •**
D.3.11.3.2	Gene therapy medicinal product[17]?	**No •**

D.3.11.3.3	Tissue Engineered Product[18]?	**No •**
D.3.11.3.4	Combination ATIMP (i.e. one involving a medical device[19])?	**No •**
D.3.11.3.5	Has the Committee on Advanced Therapies issued a classification for this product?	**No •**
D.3.11.3.5.1	If 'Yes' please provide that classification and its reference number:	
D.3.11.4	Combination product that includes a device, but does not involve an Advanced Therapy?	**No •**
D.3.11.5	Radiopharmaceutical medicinal product?	**No •**
D.3.11.6	Immunological medicinal product (such as vaccine, allergen, immune serum)?	**No •**
D.3.11.7	Plasma derived medicinal product?	**No •**
D.3.11.8	Extractive medicinal product?	**No •**
D.3.11.9	Recombinant medicinal product?	**No •**
D.3.11.10	Medicinal product containing genetically modified organisms?	**No •**
D.3.11.10.1	Has the authorisation for contained use or release been granted?	**No •**
D.3.11.10.2	Is it pending?	**No •**
D.3.11.11	Herbal medicinal product?	**No •**
D.3.11.12	Homeopathic medicinal product?	**No •**
D.3.11.13	Another type of medicinal product?	**No •**
D.3.11.13.1	If 'another type of medicinal product' specify the type of medicinal product:	
D.3.12	Mode of action (*free text*[20])	
D.3.13	Is it an IMP to be used in a first-in-human clinical trial?	**No •**
D.3.13.1	If 'Yes', are there risk factors identified, according to the guidance FIH?[21]	

**D.4**	**SOMATIC CELL THERAPY INVESTIGATIONAL MEDICINAL PRODUCT (NO GENETIC MODIFICATION)**	
D.4.1	Origin of cells	
D.4.1.1	Autologous	**No •**
D.4.1.2	Allogeneic	**No •**
D.4.1.3	Xenogeneic	**No •**
D.4.1.3.1	If 'Yes', specify the species of origin:	
D.4.2	Type of cells	
D.4.2.1	Stem cells	**No •**
D.4.2.2	Differentiated cells	**No •**
D.4.2.2.1	If 'Yes', specify the type (e.g. keratinocytes, fibroblasts, chondrocytes…):	
D.4.2.3	Others:	**No •**
D.4.2.3.1	If others, specify:	

**D.5**	**GENE THERAPY INVESTIGATIONAL MEDICINAL PRODUCTS**	
D.5.1	Gene(s) of interest:	
D.5.2	In vivo gene therapy:	**No •**
D.5.3	Ex vivo gene therapy:	**No •**
D.5.4	Type of gene transfer product	
D.5.4.1	Nucleic acid (e.g. plasmid):<br>If 'Yes', specify if:	**No •**
D.5.4.1.1	Naked:	**No •**
D.5.4.1.2	Complexed	**No •**
D.5.4.2	Viral vector:	**No •**
D.5.4.2.1	If 'Yes', specify the type: adenovirus, retrovirus, AAV, …:	
D.5.4.3	Others	**No •**

D.5.4.3.1	If others, specify:	
D.5.5	Genetically modified somatic cells:	**No** •
If 'Yes', specify the origin of the cells:		
D.5.5.1	Autologous:	**No** •
D.5.5.2	Allogeneic:	**No** •
D.5.5.3	Xenogeneic:	**No** •
D.5.5.3.1	If 'Yes', specify the species of origin:	
D.5.5.4	Specify type of cells (hematopoietic stem cells…):	

**D.6**	**TISSUE ENGINEERED PRODUCT**	
The indication which determines that this is a Tissue Engineered Product as opposed to a Cell Therapy product is given in section E.1.1.		
D.6.1	Origin of cells	
D.6.1.1	Autologous	**No** •
D.6.1.2	Allogeneic	**No** •
D.6.1.3	Xenogeneic	**No** •
D.6.1.3.1	If 'Yes', specify the species of origin:	
D.6.2	Type of cells	
D.6.2.1	Stem cells	**No** •
D.6.2.2	Differentiated cells	**No** •
D.6.2.2.1	If 'Yes', specify the type of cells(e.g. keratinocytes, fibroblasts, chondrocytes, …):	
D.6.2.3	Others:	**No** •
D.6.2.3.1	If others, specify:	

**D.7**	**PRODUCTS CONTAINING DEVICES (i.e. MEDICAL DEVICES, SCAFFOLDS ETC.)**	
D.7.1	Give a brief description of the device:	
D.7.2	What is the name of the device?	
D.7.3	Is the device implantable?	**No** •
D.7.4	Does this product contain:	
D.7.4.1	A medical device?	**No** •
D.7.4.1.1	Does this medical device have a CE mark?	**No** •
D.7.4.1.1.1	The notified body is:	
D.7.4.2	Bio-materials?	**No** •
D.7.4.3	Scaffolds?	**No** •
D.7.4.4	Matrices?	**No** •
D.7.4.5	Other?	**No** •
D.7.4.5.1	If other, specify:	

D.8 INFORMATION ON PLACEBO (if relevant; repeat as necessary)

D.8.1	Is there a placebo:	**No** •

D.8.2	This refers to placebo number:	
D.8.3	Pharmaceutical form:	
D.8.4	Route of administration:	
D.8.5	Which IMP is it a placebo for? Specify IMP Number(s) from D.1.1	
D.8.5.1	Composition, apart from the active substance(s):	
D.8.5.2	Is it otherwise identical to the IMP?	**Yes** ? **No** ? **Not Answered** ?

D.8.5.2.1	If not, specify major ingredients:

D.9 SITE(S) WHERE THE QUALIFIED PERSON CERTIFIES BATCH RELEASE[22]

*This section is dedicated to **finished** IMPs, i.e. medicinal products randomised, packaged, labelled and certified for use in the clinical trial. If there is more than one site or more than one IMP is certified, use extra pages and give each IMP its number from section D.1.1 or D.8.2 In the case of multiple sites indicate the product certified by each site*

D.9.1	Do not fill in section D.9.2 for an IMP that:<br>*Has a MA in the EU **and***<br>*Is sourced from the EU market **and***<br>*Is used in the trial without modification(e.g. not overencapsulated) **and***<br>*The packaging and labelling is carried out for local use only as per article 9.2. of the Directive 2005/28/EC (GCP Directive)*<br>If all these conditions are met tick ● and list the number(s) of each IMP including placebo from sections D.1.1 and D.8.2 to which this applies<br>**PR1**

**D.9.2**	**Who is responsible in the Community for the certification of the finished IMPs?**	
	This site is responsible for certification of (list the number(s) of each IMP including placebo from sections D.1.1 and D.8.2):<br>please tick the appropriate box:	
D.9.2.1	Manufacturer	**?**
D.9.2.2	Importer	**?**
D.9.2.3	Name of the organisation:	
D.9.2.4	Address:	
D.9.2.4.1	Street Address	
D.9.2.4.2	Town/City	
D.9.2.4.3	Post Code	
D.9.2.4.4	Country	
D.9.2.5	Give the manufacturing authorisation number:	
D.9.2.5.1	If No authorisation, give the reasons:	

Where the product does not have a MA in the EU, but is supplied in bulk and final packaging and labelling for local use is carried out in accordance with article 9.2 of Directive 2005/28/EC (GCP Directive) then enter the site where the product was finally certified for release by the Qualified Person for use in the clinical trial at D.9.2 above.

E. GENERAL INFORMATION ON THE TRIAL

This section should be used to provide information about the aims, scope and design of the trial. When the protocol includes a sub-study in the MS concerned section E.2.3 should be completed providing information about the sub-study. To identify it check the sub-study box in the 'Objective of the trial' question below.

**E.1**	**MEDICAL CONDITION OR DISEASE UNDER INVESTIGATION**	
E.1.1	Specify the medical condition(s) to be investigated[23] (free text):	
	**English**	**Aspartylglucosaminuria**
	**Finnish**	**Aspartyyliglukosaminuria**
E.1.1.1	Medical condition in easily understood language	
	**English**	**Aspartylglucosaminuria (AGU) is a progressive disease that results in severe mental retardation of the patients. The main symptom of the disease is the progressive loss of mental capabilities.**
	**Finnish**	**Aspartyyliglukosaminuria (AGU) on vaikeaa kehitysvammaisuutta aiheuttava, etenevä lastenneurologinen tauti. Taudin pääoire on henkisen kehityksen taantuminen.**
E.1.1.2	Therapeutic area<br>**Diseases [C] - Congenital, Hereditary, and Neonatal Diseases and Abnormalities [C16]**	
E.1.2	MedDRA version, system organ class, level, term and classification code[24]:<br>Version System Organ Class Classification Code Term Level	
E.1.3	Is any of the conditions being studied a rare disease[25]? **Yes •**	

**E.2**	**OBJECTIVE OF THE TRIAL**	
E.2.1	Main objective:	
	**English**	**Safety and efficacy of Cystadane in the treatment of AGU**
	**Finnish**	**Cystadanen teho ja turvallisuus AGU-taudin hoidossa**
E.2.2	Secondary objectives:	
	**English**	**Urine glycoasparagines**<br>**AGA enzyme activity in white blood cells**<br>**Cognitive function and psychological tests**<br>**Quality of life survey**<br>**Adaptive skills**<br>**MRI findings of the brain**<br>**Methionin level**<br>**Absence of adverse events**
	**Finnish**	**Glykoasparagiinien eritys virtsaan**<br>**AGA-entsyymin aktiivisuus valkosoluissa**<br>**Henkinen kehitys ja psykologiset testit**<br>**Elämänlaatukysely**<br>**Arjen toimintakyky**<br>**Aivojen magneettilödökset**<br>**Metioniinipitoisuus**<br>**Vakavien haittavaikutusten puuttuminen**
E.2.3	Is there a sub-study? **No •**	
E.2.3.1	If 'Yes', give the full title, date and version of each sub-study and their related objectives:	

E.3	PRINCIPAL INCLUSION CRITERIA *(list the most important)*	
	**English**	**Patients below the age of 15 years who have aspartylglucosaminuria**<br>**Preferably homozygous for the Finnish major AGU mutation, AGU-Fin-major**<br>**Written informed consent of the parents**<br>**No known hypersensitivity or allergy against betain**<br>**Compliance of the patient**
	**Finnish**	**Alle 15-vuotiaat potilaat, jotka sairastavat aspartyyliglukosaminuriaa**<br>**Erityisesti potilaat, jotka ovat homotsygootteja suomalaisen valtamutaation, AGU-Fin-major, suhteen**<br>**Kirjallinen suostumus vanhemmilta**<br>**Ei tunnettua yliherkkyyttä betaiinille**<br>**Potilaan hyväksi arvioitu yhteistyökyky**

E.4	PRINCIPAL EXCLUSION CRITERIA *(list the most important)*	
	**English**	**Age above 15 years**<br>**Patients who do not carry at least one allele of the Finnish major mutation**<br>**Known adverse reactions against Betain or components of Cystadane**<br>**Known history of cerebral oedema□**<br>**Participating in other interventional clinical studies**<br>**Patients who have undergone bone marrow tranplantation**<br>**Co-medication which would interfere with administration of Betain**
	**Finnish**	**Yli 15-vuotiaat AGU-potilaat**<br>**AGU-potilaat, joilla ei kummassakaan alleelissa ole suomalaista valtamutaatiota**<br>**Tunnettu yliherkkyys betaiinille tai Cystadanen ainesosille**<br>**Aikaisempi havainto aivopaineen noususta**<br>**Potilaan osallistuminen muihin interventionaalisiin kliinisiin lääketutkimuksiin**<br>**Potilaat, joille on tehty luuydinsiirto**<br>**Muu lääkitys, joka saattaisi häiritä betaiinin antamista**

E.5	END POINT(S):	
E.5.1	Primary End Point (repeat as necessary)[26]	
	**English**	**Primary endpoint is the urinary excretion of glycoasaparagines**
	**Finnish**	**Ensisijainen päätemuuttuja on glykoasparagiinien määrä virtsassa.**
E.5.1.1	Timepoint(s) of evaluation of this end point	
	**English**	**0 months (starting point)**<br>**3 months**<br>**6 months**<br>**12 months**<br>**15 months**<br>**24 months**<br>**36 months**<br>**48 months**
	**Finnish**	**0 kk (alkuarvo)**<br>**3 kk**

6 kk
12 kk
15 kk
24 kk
36 kk
48 kk

E.5.2 Secondary End Point (repeat as necessary)

English **AGA enzyme activity in white blood cells**
Psychological tests
Visit at child neurologists
Quality of life survey
Adaptive skills
MRI findings of the brain
Methionin Level in serum
Absence of adverse events

Finnish **AGA-entsyymin aktiivisuus valkosoluissa**
Psykologiset testit
Lastenneurologin tutkimus
Elämänlaatukysely
Arjen toimintakyky
Aivojen magneettikuvaus
Metioniinipitoisuus seerumissa
Vakavien haittavaikutusten puuttuminen

E.5.2.1 Timepoint(s) of evaluation of this end point

English **AGA enzyme activity in white blood cells: 0, 3, 6, 12, 15, 24, 36, 48 months**
Psychological tests: 0, 24, 48 months
Child neurologist: 0,3 , 12, 15, 24, 36, 48 months
Quality of life survey: 0, 12, 24, 36, 48 months
Adaptive skills: 0, 12, 24, 36, 48 months
MRI findings of the brain: 0, 12, 24, 36, 48 months
Methionin level: 0, 3, 6, 12, 24 months
Absence of adverse events: continuously

Finnish **AGA-entsyymin aktiivisuus valkosoluissa: 0, 3, 6, 12, 15, 24, 36, 48 kk**
Psykologiset testit: 0, 24, 48 kk
Lastenneurologin tutkimus: 0, 3, 12, 15, 24, 36, 48
Elämänlaatukysely: 0, 12, 24, 36, 48 kk
Arjen toimintakyky: 0, 12, 24, 36, 48 kk
Aivojen magneettikuvaus: 0, 12, 24, 36, 48 kk
Metioniinipitoisuus: 0, 3, 6, 12, 24 kk
Vakavien haittavaikutusten puuttuminen: jatkuvasti

**E.6**	**SCOPE OF THE TRIAL – Tick all boxes where applicable**	
E.6.1	Diagnosis	**No •**
E.6.2	Prophylaxis	**No •**
E.6.3	Therapy	**Yes •**
E.6.4	Safety	**Yes •**
E.6.5	Efficacy	**Yes •**
E.6.6	Pharmacokinetic	**No •**
E.6.7	Pharmacodynamic	**No •**
E.6.8	Bioequivalence	**No •**
E.6.9	Dose Response	**No •**
E.6.10	Pharmacogenetic	**No •**
E.6.11	Pharmacogenomic	**No •**

E.6.12	Pharmacoeconomic	**No •**
E.6.13	Others	**No •**
E.6.13.1	If others, specify:	

**E.7**	**TRIAL TYPE AND PHASE[27]**	
E.7.1	Human pharmacology (Phase I)	**No •**
Is it:		
E.7.1.1	First administration to humans	**No •**
E.7.1.2	Bioequivalence study	**No •**
E.7.1.3	Other:	**No •**
E.7.1.3.1	If other, please specify:	
E.7.2	Therapeutic exploratory (Phase II)	**Yes •**
E.7.3	Therapeutic confirmatory (Phase III)	**No •**
E.7.4	Therapeutic use(Phase IV)	**No •**

**E.8**	**DESIGN OF THE TRIAL**	
E.8.1	Controlled	**Yes •**
	If 'Yes', specify:	
E.8.1.1	Randomised:	**No •**
E.8.1.2	Open:	**Yes •**
E.8.1.3	Single blind:	**No •**
E.8.1.4	Double blind:	**No •**
E.8.1.5	Parallel group:	**No •**
E.8.1.6	Cross over:	**No •**
E.8.1.7	Other:	**No •**
E.8.1.7.1	If other specify:	
E.8.2	If controlled, specify the comparator:	
E.8.2.1	Other medicinal product(s)	**No •**
E.8.2.2	Placebo	**No •**
E.8.2.3	Other	**Yes •**
E.8.2.3.1	If 'Yes' to other, specify :	
	**Finnish**	**3 kk tauko lääkkeen käytössä 12 kk jälkeen**
	**English**	**Treatment break of 3 months after 12 months of use**
E.8.2.4	Number of treatment arms in the trial	**1**
E.8.3	Single site in the Member State concerned (see also section G):	**Yes •**
E.8.4	Multiple sites in the Member State concerned(see also section G):	**No •**
E.8.4.1	Number of sites anticipated in Member State concerned	
E.8.5	Multiple Member States:	**No •**
E.8.5.1	Number of sites anticipated in the EEA:	
E.8.6	Trial involving sites outside the EEA:	
E.8.6.1	Trial being conducted both within and outside the EEA:	**No •**
E.8.6.2	Trial being conducted completely outside of the EEA:	**No •**
E.8.6.3	If E.8.6.1 or E.8.6.2 are Yes, specify the regions in which trial sites are planned:	
E.8.6.4	If E.8.6.1 or E.8.6.2 are Yes, specify the number of sites anticipated outside of the EEA:	
E.8.7	Trial having an independent data monitoring committee:	**No •**
E.8.8	Definition of the end of trial: If it is the last visit of the last subject, please enter "LVLS". If it is not LVLS provide the definition:	
	**English**	**After 48 months of starting the test medication**
	**Finnish**	**48 kk päästä lääkkeen käytön aloittamisesta**
E.8.9	Initial estimate of the duration of the trial[28] (years, months and days)	
E.8.9.1	In the Member State concerned	**4 years months days**
E.8.9.2	In all countries concerned by the trial	**years months days**
E.8.10	Proposed date of start of recruitment	
E.8.10.1	In the Member State concerned	**2017-09-30**

E.8.10.2 In any country

F. POPULATION OF TRIAL SUBJECTS

F.1	AGE RANGE		
F.1.1	Are the trial subjects under 18?		**Yes •**
	If 'Yes', specify the estimated number of subjects planned in each age range for the whole trial:		**15**
		Approx. No. of patients[29]	
F.1.1.1	In utero	()	**No •**
F.1.1.2	Preterm newborn infants (up to gestational age < 37 weeks)	()	**No •**
F.1.1.3	Newborns (0-27 days)	()	**No •**
F.1.1.4	Infants and toddlers (28 days - 23 months)	()	**No •**
F.1.1.5	Children (2-11 years)	(**12**)	**Yes •**
F.1.1.6	Adolescents (12-17 years)	(**3**)	**Yes •**
F.1.2	Adults (18-64 years)	()	**No •**
F.1.3	Elderly (>= 65 years)	()	**No •**

F.2	GENDER	
F.2.1	Female	**Yes •**
F.2.2	Male	**Yes •**

F.3	GROUP OF TRIAL SUBJECTS	
F.3.1	Healthy volunteers	**No •**
F.3.2	Patients	**Yes •**
F.3.3	Specific vulnerable populations	**Yes •**
F.3.3.1	Women of child bearing potential not using contraception	**No •**
F.3.3.2	Women of child bearing potential using contraception	**No •**
F.3.3.3	Pregnant women	**No •**
F.3.3.4	Nursing women	**No •**
F.3.3.5	Emergency situation	**No •**
F.3.3.6	Subjects incapable of giving consent personally	**Yes •**
F.3.3.6.1	If 'Yes', specify:	
	**English** **Children, handicapped with a mental retardation**	
	**Finnish** **Lapsipotilaat, joilla on kehitysvamma**	
F.3.3.7	Others:	**No •**
F.3.3.7.1	If 'Yes', specify:	

F.4	PLANNED NUMBER OF SUBJECTS TO BE INCLUDED:	
F.4.1	In the member state	**15**
F.4.2	For a multinational trial**:**	
F.4.2.1	In the EEA	
F.4.2.2	In the whole clinical trial	

F.5	PLANS FOR TREATMENT OR CARE AFTER THE SUBJECT HAS ENDED HIS/HER PARTICIPATION IN THE TRIAL. please specify (free text):	
	**English**	**Standard treatment of the disease. In case a follow-up study is planned, possible inclusion.**
	**Finnish**	**AGU-taudin tavanomainen hoito. Jos tutkimukselle on suunnitteille jatkovaihe, mahdollisesti siihenkin osallistuminen.**

G. CLINICAL TRIAL SITES/INVESTIGATORS IN THE MEMBER STATE CONCERNED BY THIS REQUEST

**G.1**	**CO-ORDINATING INVESTIGATOR (for multicentre trial) and principal investigator (for single centre trial)**	
G.1.1	Given name:	**Minna**
G.1.2	Middle name, if applicable:	
G.1.3	Family name:	**Laine**
G.1.4	Qualification (MD……….)	**MD, PhD, child neurologist**
G.1.5	Professional address:	
G.1.5	Institution name	**Helsinki University Hospital**
G.1.5	Institution department	**Peijas Hospital**
G.1.5.1	Street address	**Sairaalakatu 1**
G.1.5.2	Town/city	**Vantaa**
G.1.5.3	Post code	**00029 HUS**
G.1.5.4	Country	**Finland**
G.1.6	Telephone number:	
G.1.7	Fax number:	
G.1.8	E-mail:	**Minna.Laine@hus.fi**

**G.2**	**PRINCIPAL INVESTIGATORS** ***(for multicentre trial ; where necessary, use additional forms)***
G.2.1	Given name:
G.2.2	Middle name, if applicable:
G.2.3	Family name:
G.2.4	Qualification (MD……….)
G.2.5	Professional address:
G.2.5	Institution name
G.2.5	Institution department
G.2.5.1	Street address
G.2.5.2	Town/city
G.2.5.3	Post code
G.2.5.4	Country
G.2.6	Telephone number:
G.2.7	Fax number:
G.2.8	E-mail:

**G.3**	**CENTRAL TECHNICAL FACILITIES TO BE USED IN THE CONDUCT OF THE TRIAL**<br><br>**Laboratory or other technical facility, in which the measurement or assessment of the main evaluation criteria are centralised** (repeat as needed for multiple organisations).	
G.3.1	Name of organisation:	**Islab**
G.3.2	Department	**Pohjois-Savon Aluelaboratorio**
G.3.3	Name of contact person:	
G.3.3.1	Given name	
G.3.3.2	Middle name	
G.3.3.3	Family name	
G.3.4	Address:	
G.3.4.1	Street address	**Puijonlaaksontie 2**
G.3.4.2	Town/city	**Kuopio**
G.3.4.3	Post code	**70211**
G.3.4.4	Country	**Finland**
G.3.5	Telephone number:	**358 44 717 8720**
G.3.6	Fax number:	
G.3.7	E-mail:	
G.3.8	Enter the details of any duties subcontracted to this central technical facility in this trial	
G.3.8.1	Routine clinical pathology testing	**No •**

G.3.8.2	Clinical chemistry	**Yes •**
G.3.8.3	Clinical haematology	**No •**
G.3.8.4	Clinical microbiology	**No •**
G.3.8.5	Histopathology	**No •**
G.3.8.6	Serology/ endocrinology	**No •**
G.3.8.7	Analytical chemistry	**No •**
G.3.8.8	ECG analysis/ review	**No •**
G.3.8.9	Medical image analysis/ review - X-ray, MRI, ultrasound, etc.	**No •**
G.3.8.10	Primary/ surrogate endpoint test	**Yes •**
G.3.8.11	Other Duties subcontracted?	**No •**
G.3.8.11.1	If 'Yes', specify the other duties	

**G.4**	**NETWORKS TO BE INVOLVED IN THE TRIAL (e.g. Paediatric Networks involved in the trial)**	
G.4.1	Name of organisation:	**University of Giessen**
G.4.2	Name of contact person:	
G.4.2.1	Given name	**Ritva**
G.4.2.2	Middle name	
G.4.2.3	Family name	**Tikkanen**
G.4.3	Address:	
G.4.3.1	Street address	**Friedrichstrasse 24**
G.4.3.2	Town/city	**Giessen**
G.4.3.3	Post code	**D-35392**
G.4.3.4	Country	**Germany**
G.4.4	Telephone number:	
G.4.5	Fax number:	
G.4.6	E-mail:	**ritva.tikkanen@biochemie.med.uni-giessen.de**
G.4.7	Activities carried out by the network:<br>**Scientific PI,<br>responsible for scientific data, contact with Orphan Europe<br>Measurement of leukocyte AGA activity**	

**G.4**	**NETWORKS TO BE INVOLVED IN THE TRIAL (e.g. Paediatric Networks involved in the trial)**	
G.4.1	Name of organisation:	**Prof. Taina Autti**
G.4.2	Name of contact person:	
G.4.2.1	Given name	**Taina**
G.4.2.2	Middle name	
G.4.2.3	Family name	**Autti**
G.4.3	Address:	
G.4.3.1	Street address	**PL 340**
G.4.3.2	Town/city	**Helsinki**
G.4.3.3	Post code	**00029 HUS**
G.4.3.4	Country	**Finland**
G.4.4	Telephone number:	
G.4.5	Fax number:	
G.4.6	E-mail:	**Taina.Autti@hus.fi**
G.4.7	Activities carried out by the network:<br>**MRI Imaging and analysis of the respective data**	

**G.4**	**NETWORKS TO BE INVOLVED IN THE TRIAL (e.g. Paediatric Networks involved in the trial)**	
G.4.1	Name of organisation:	**Prof. Päivi Helenius**
G.4.2	Name of contact person:	
G.4.2.1	Given name	**Päivi**
G.4.2.2	Middle name	

G.4.2.3	Family name	**Helenius**
G.4.3	Address:	
G.4.3.1	Street address	**Diagnostis-terapeuttinen osasto, PL340**
G.4.3.2	Town/city	**Helsinki**
G.4.3.3	Post code	**00029 HUS**
G.4.3.4	Country	**Finland**
G.4.4	Telephone number:	
G.4.5	Fax number:	
G.4.6	E-mail:	**Paivi.helenius@hus.fi**
G.4.7	Activities carried out by the network: **Neuropsychological testing, quality of life survey**	

**G.5**	**ORGANISATIONS TO WHOM THE SPONSOR HAS TRANSFERRED TRIAL RELATED DUTIES AND FUNCTIONS**	
G.5.1	**Has the sponsor transferred any major or all the sponsor's trial related duties and functions to another organisation or third party?**	**Yes •**

Repeat as necessary for multiple organisations:

G.5.1.1	Organisation name:	
G.5.1.2	Organisation department	
G.5.1.3	Name of contact person :	
G.5.1.3.1	Given name	
G.5.1.3.2	Middle name	
G.5.1.3.3	Family name	
G.5.1.4	Address:	
G.5.1.4.1	Street address	
G.5.1.4.2	Town/city	
G.5.1.4.3	Post code	
G.5.1.4.4	Country	
G.5.1.5	Telephone number:	
G.5.1.6	Fax number:	
G.5.1.7	E-mail:	
G.5.1.8	All tasks of the sponsor	**No •**
G.5.1.9	Monitoring	**No •**
G.5.1.10	Regulatory (e.g. preparation of applications to CA and ethics committee)	**No •**
G.5.1.11	Investigator recruitment	**No •**
G.5.1.12	IVRS[30] – treatment randomisation	**No •**
G.5.1.13	Data management	**No •**
G.5.1.14	E-data capture	**No •**
G.5.1.15	SUSAR reporting	**No •**
G.5.1.16	Quality assurance auditing	**No •**
G.5.1.17	Statistical analysis	**No •**
G.5.1.18	Medical writing	**No •**
G.5.1.19	Other duties subcontracted?	**No •**
G.5.1.19.1	If 'Yes' to other, please specify:	

H. COMPETENT AUTHORITY / ETHICS COMMITTEE IN THE MEMBER STATE CONCERNED BY THIS REQUEST

**H.1**	**TYPE OF APPLICATION**	
If this application is addressed to the Competent Authority, please tick the Ethics Committee box and give information on the Ethics committee concerned. If this application is addressed to the Ethics Committee, please tick the Competent Authority box and give the information on the Competent Authority concerned.		
H.1.1	Competent Authority	**No •**
H.1.2	Ethics Committee	**Yes •**

**H.2**	**INFORMATION ON ETHICS COMMITTEE**	
H.2.1	Name:	**Unknown**
H.2.2	Address	
H.2.2.1	Street address	
H.2.2.2	Town/city	
H.2.2.3	Post code	
H.2.2.4	Country	
H.2.3	Date of submission:	

**H.3**	**OPINION**	
H.3.1	To be requested	**Yes •**
H.3.2	Pending	**No •**
H.3.3	Given	**No •**
	If 'Given', specify:	
H.3.3.1	Date of opinion:	
H.3.3.2	Opinion favourable	**No •**
H.3.3.3	Opinion not favourable	**No •**
	If not favourable, give:	
H.3.3.3.1	The reasons	
H.3.3.3.2	The eventual anticipated date of resubmission:	

I. SIGNATURE OF THE APPLICANT IN THE MEMBER STATE

**I.1**	I hereby confirm that /confirm on behalf of the sponsor (delete which is not applicable) that:<br>• the information provided is complete;<br>• the attached documents contain an accurate account of the information available;<br>• the clinical trial will be conducted in accordance with the protocol; and<br>• the clinical trial will be conducted, and SUSARs and result-related information will be reported, in accordance with the applicable legislation.

**I.2**	**APPLICANT OF THE REQUEST FOR THE COMPETENT AUTHORITY** (as stated in section C.1):
I.2.1	Date:
I.2.2	Signature[31]:
I.2.3	Print name:

**I.3**	**APPLICANT OF THE REQUEST FOR THE ETHICS COMMITTEE** (as stated in section C.2):
I.3.1	Date:
I.3.2	Signature[32]:
I.3.3	Print name:

ENDNOTES

[1] Any translation of the protocol should be assigned the same date and version as those in the original document.
[2] International Standard Randomised Controlled Trial Number. Sponsors may wish to use an International Standardised Random Controlled Trial Number (ISRCTN) to identify their trial in addition to the EudraCT number; for instance if their trial is part of a multinational trial with sites outside the Community. They can obtain the number and guidance from the Current Controlled Trials website http://www.controlled-trials.com/isrctn to which there is a link from the EudraCT database website http://eudract.ema.europa.eu. When available they should provide it in Section A.6 of the application form.
[3] US National Clinical Trial (NCT) Numbers required on the FDA clinical trial application form.
[4] For a resubmission following previous withdrawal of an application or unfavourable opinion of an ethics committee, or previous withdrawal of an application or refusal of a request by the competent authority, enter a letter in the sequence, A for first resubmission, B for second, C for third et seq.
[5] In accordance with Article 19 of Directive 2001/20/EC.
[6] The contact point should give functional information rather than details of one "person", in order to avoid the need for update and maintenance of these contact details.
[7] This requires a EudraLink account. (See https://eudract.ema.europa.eu/document.html for details)
[8] According to national legislation.
[9] Available from the Summary of Product Characteristics (SmPC)
[10] According to the Community register on orphan medicinal products (Regulation (EC) n° 141/2000): http://ec.europa.eu/enterprise/pharmaceuticals/register/index.htm
[11] Committee for Medicinal Products for Human Use of the European Medicines Agency
[12] To be provided only when there is No trade name. This is the name routinely used by a sponsor to identify the IMP in the CT documentation (protocol, IB…).
[13] To be provided only when there is No trade name. This is a code designated by the sponsor which represents the name routinely used by the sponsor to identify the product in the CT documentation. For example, a code may be used for combinations of drugs or drugs and devices.
[14] Available from the Summary of Product Characteristics (SmPC).
[15] Chemical Abstracts Service.
[16] Complete also section D.4 Cell therapy as defined in Annex 1 part IV of Directive 2001/83/EC as amended.
[17] Complete also section D.5 Gene Therapy as defined in Annex 1 part IV of Directive 2001/83/EC as amended.
[18] Complete also section D.6 - Tissue Engineered Product as defined in Article 2(1)(b) of Regulation1394/2007/EC.
[19] Complete also section D.7
[20] The mode of action should briefly describe the chemical, biochemical, immunological or biological means the IMP uses to effect its pharmaceutical action.
[21] Guideline on strategies to identify and mitigate risks for first-in-human clinical trials with investigational medicinal products. EMEA/CHMP/SWP/28367/2007 19 July 2007
[22] In accordance with paragraph 38 of Annex 13 of Volume 4 of the Rules Governing Medical Products in the European Union.
[23] In the case of healthy volunteer trials, the intended indication for the product under development should be provided.
[24] Applicants are encouraged to provide the MedDRA lower level term if applicable and classification code. These can be accessed from the EMEA EudraCT website (http://eudract.ema.europa.eu/).
[25] Points to consider on the calculation and reporting of the prevalence of a condition for Orphan drug designation: COM/436/01 (http://www.ema.europa.eu/htms/human/orphans/intro.htm).
[26] The protocol will usually identify a single primary end point but there may be a co-primary end point in some cases and/or a number of secondary end points.
[27] The descriptions of the trial types provided are those recommended in preference to Phases. See page 5 of Community guideline CPMP/ICH/291/95. The development of a new indication after initial approval of a medicine should be considered as a new development plan.
[28] From the first inclusion until the last visit of the last subject.
[29] These numbers will be initial estimates. Applicants will not be required to update this information nor do they constitute an authorisation or restriction on the inclusion of these numbers of patients in the trial. The numbers of subjects whose inclusion is authorised are those set out in the authorised version of the protocol, or subsequent authorised amendments.
[30] Interactive Voice Response System: commonly used for randomisation of treatment and controlling the shipment of stock of product.
[31] On an application to the Competent Authority only, the applicant to the Competent Authority needs to sign.

[32] On an application to the Ethics Committee only, the applicant to the Ethics Committee needs to sign.
