# Supplementary material for: Pharmacological chaperone treatment with Cystadane in aspartylglucosaminuria: an open-label, phase Ib/II trial
Source: Nat Commun. 2026 Nov 11;17:11604. doi: 10.1038/s41467-026-78752-x (PMC13754814; doi:10.1038/s41467-026-78752-x)
Supplement: Supplementary file 2 [file 41467_2026_78752_MOESM2_ESM.pdf]

Supplementary Data 1. LC-MS raw data for the quantification of GlcNAc-Asn. 20 µl of 80-fold diluted urine samples were injected as technical triplicates into an Alliance HT 2790 High-Performance Liquid Chromatography system coupled to a Waters Micromass ZQ 4000 mass detector (Waters GmbH, Eschborn, Germany). The peak areas were recorded with the MassLynx software, version 4.0 (Waters GmbH). Aberrant read-outs due to injection errors were excluded from the data analysis, resulting in technical duplicates instead of triplicates in some cases (Table sheet "single values"). The mean peak areas were calculated (Table sheet "mean values"). For the quantification, different amounts (39 pmol to 1.2 nmol) of pure GlcNAc-Asn as calibrator were injected in triplicates, and the mean peak areas were plotted against the concentrations. The resulting standard curve showed high linearity ($R^2 > 0.99$). The slope and the intercept of the standard curve were used to convert the peak areas into pmol GlcNAc-Asn.
