# Supplementary material for: Pharmacological chaperone treatment with Cystadane in aspartylglucosaminuria: an open-label, phase Ib/II trial
Source: Nat Commun. 2026 Nov 11;17:11604. doi: 10.1038/s41467-026-78752-x (PMC13754814; doi:10.1038/s41467-026-78752-x)
Supplement: Supplementary file 3 [file 41467_2026_78752_MOESM4_ESM.pdf]

Corresponding author(s): Ritva Tikkanen

Last updated by author(s): Sep 3, 2026

Reporting Summary

Nature Portfolio wishes to improve the reproducibility of the work that we publish. This form provides structure for consistency and transparency in reporting. For further information on Nature Portfolio policies, see our Editorial Policies and the Editorial Policy Checklist.

Statistics

For all statistical analyses, confirm that the following items are present in the figure legend, table legend, main text, or Methods section.

n/a	Confirmed	
☐	☒	The exact sample size (n) for each experimental group/condition, given as a discrete number and unit of measurement
☐	☒	A statement on whether measurements were taken from distinct samples or whether the same sample was measured repeatedly
☐	☒	The statistical test(s) used AND whether they are one- or two-sided<br>*Only common tests should be described solely by name; describe more complex techniques in the Methods section.*
☐	☒	A description of all covariates tested
☐	☒	A description of any assumptions or corrections, such as tests of normality and adjustment for multiple comparisons
☐	☒	A full description of the statistical parameters including central tendency (e.g. means) or other basic estimates (e.g. regression coefficient) AND variation (e.g. standard deviation) or associated estimates of uncertainty (e.g. confidence intervals)
☐	☒	For null hypothesis testing, the test statistic (e.g. F, t, r) with confidence intervals, effect sizes, degrees of freedom and P value noted<br>*Give P values as exact values whenever suitable.*
☒	☐	For Bayesian analysis, information on the choice of priors and Markov chain Monte Carlo settings
☒	☐	For hierarchical and complex designs, identification of the appropriate level for tests and full reporting of outcomes
☐	☒	Estimates of effect sizes (e.g. Cohen's d, Pearson's r), indicating how they were calculated

Our web collection on statistics for biologists contains articles on many of the points above.

Software and code

Policy information about availability of computer code

Data collection	Software: LC-MA analysis: MassLynx software, version 4.0 ; Plate reader: Tecan i-control Version 3.9.1.0;<br>In the analysis of MRI images, we have used publicly available tools such as FreeSurfer, FSL, and ANTs, and an MIT license. The analysis scripts to run these programs are shared in Github with additional Python script that was used for the model fitting of the results.<br>The MRI analysis codes are freely shared via Github. https://github.com/vilsaira/AGU_SWI_analysis and https://doi.org/10.5281/zenodo.22141812.
Data analysis	Software: LMM model fitting: R Core Team, version 4.6.0; Statistics: GraphPad Prism version 11;<br>MRI image analysis: Two prediction models were constructed from the thalamic iron accumulation data derived from the SW-based MR images of the study participants at the beginning of the study. The linear model predicts a continuous increase of iron accumulation, whereas according to the Gompertz model, a plateau is reached around 15 years of age. Linear model and the Gompertz model were fitted to the extracted phase-signals of susceptibility-weighted images to obtain 95% prediction intervals, coefficient of determination (R2), and mean-squared error (MSE).

For manuscripts utilizing custom algorithms or software that are central to the research but not yet described in published literature, software must be made available to editors and reviewers. We strongly encourage code deposition in a community repository (e.g. GitHub). See the Nature Portfolio guidelines for submitting code & software for further information.

Data

Policy information about availability of data

All manuscripts must include a data availability statement. This statement should provide the following information, where applicable:

- Accession codes, unique identifiers, or web links for publicly available datasets
- A description of any restrictions on data availability
- For clinical datasets or third party data, please ensure that the statement adheres to our policy

Source data are provided with this paper. Individual participant MRI data that underlie the results reported in this article are available after de-identification (text, tables, figures). These data will be shared with investigators whose proposed use of the data has been approved by an independent ethical committee that should be identified upon submitting an application for data use as disclosed in the application. Applications are to be submitted to Ritva.Tikkanen@biochemie.med.uni-giessen.de. Study protocol and analytical codes are available as Supplementary Information. Patient materials (urine and blood samples) are not freely available due to data safety concerns.
The MRI analysis codes are freely shared via Github. https://github.com/vilsaira/AGU_SWI_analysis and https://doi.org/10.5281/zenodo.22141812.

Research involving human participants, their data, or biological material

Policy information about studies with human participants or human data. See also policy information about sex, gender (identity/presentation), and sexual orientation and race, ethnicity and racism.

Reporting on sex and gender	The sex (based on biological assignment) and age of the patients are reported in this manuscript. The data were reported without sex- or gender-based analysis due to the limited number of patients.<br>Sex and gender of the participants was not relevant for the study design due to the specific features of the study population.
Reporting on race, ethnicity, or other socially relevant groupings	Not applicable. No race, ethnicity etc. were reported due to the specific nature of the study population.
Population characteristics	25 patients with a confirmed diagnosis for aspartylglucosaminuria, age 3 to 17 years, were included in the study. All patients are homozygous for a specific pathogenic variant of the AGA gene.<br>The study population represents a specific vulnerable population, as all the patients were minors (below 18 years of age) who have a neurodevelopmental disorder with intellectual disability.
Recruitment	The patients were identified in collaboration with the Finnish AGU patient organization, Suomen AGU r.y., and recruited to the study by M.L. The study population represents a specific vulnerable population, as all the patients were minors (below 18 years of age) who are mentally handicapped who have a neurodevelopmental disorder with intellectual disability and are thus not capable of personally giving an informed consent. The informed consent form was signed by a parent, or a person who legally represents the patient. The patients did not receive any compensation for their participation.
Ethics oversight	Ethical comitte for women, children and for psychiatry, Helsinki and Uusimaa Region, Finland; permission number HUS/2604/2017

Note that full information on the approval of the study protocol must also be provided in the manuscript.

Field-specific reporting

Please select the one below that is the best fit for your research. If you are not sure, read the appropriate sections before making your selection.

☒ Life sciences ☐ Behavioural & social sciences ☐ Ecological, evolutionary & environmental sciences

For a reference copy of the document with all sections, see nature.com/documents/nr-reporting-summary-flat.pdf

Life sciences study design

All studies must disclose on these points even when the disclosure is negative.

Sample size	Original study population: 21 patients with AGU. Four further enrolled patients were not analyzed due to too short observation time. The number of samples (n) has been declared in the figure legends.
Data exclusions	Serum samples that were clearly red were excluded from AGA activity measurement, as haemolysis is known to inhibit AGA activity. This is also defined in our full study plan. Data of the four patients enrolled after 15 months were not analyzed due to too short observation times.
Replication	The AGA activity measurements were performed in technical triplicates using a validated activity measurement strategy. All attempts to replicate were successful. Some of the data, such as the MRI studies, neuropsychological studies, cannot be replicated due to the nature of the clinical study.
Randomization	No randomization was performed due to the low amount of patients and the nature of the study (open-label, single-arm, single-center).

Blinding | Blinding not relevant to the study as this is an open-label, single-arm, single-center study. Codified patient numbers were used.

Reporting for specific materials, systems and methods

We require information from authors about some types of materials, experimental systems and methods used in many studies. Here, indicate whether each material, system or method listed is relevant to your study. If you are not sure if a list item applies to your research, read the appropriate section before selecting a response.

Materials & experimental systems

n/a	Involved in the study
☒	☐ Antibodies
☒	☐ Eukaryotic cell lines
☒	☐ Palaeontology and archaeology
☒	☐ Animals and other organisms
☐	☒ Clinical data
☒	☐ Dual use research of concern
☒	☐ Plants

Methods

n/a	Involved in the study
☒	☐ ChIP-seq
☒	☐ Flow cytometry
☐	☒ MRI-based neuroimaging

Clinical data

Policy information about clinical studies

All manuscripts should comply with the ICMJE guidelines for publication of clinical research and a completed CONSORT checklist must be included with all submissions.

Clinical trial registration | EudraCT number: 2017-000645-48

Study protocol | Full trial protocol with statistical analysis plan has been provided in Supplementary Information.

Data collection | Single-center study at the University of Helsinki, Finland, carried out for 48 months between March 2018 and April 2022; recruitment period from Nov 2017 until Feb 2018. Biochemical data analysis: University of Giessen, Giessen, Germany.
Upon enrollment, the patients received a codified number that was used to track and label all the samples and the data obtained during the study. A database containing patient IDs and the codified numbers was established and kept by Dr. M. Laine in the University Hospital of Helsinki.

Outcomes | Primary endpoint: urine glycoasparagines (quantitative, by LC-MS); secondary endpoints: AGA enzyme activity in the serum (activity measurement), Neuropsychological Tests (WISC-IV), Visit to child neurologists, Quality of life Survey (Kid & Kiddo KINDL), Adaptive skills (ABAS-3), MRI findings of the brain, Methionine level in the plasma (laboratory test), Absence of severe adverse events (clinical examination & parent interview)

Plants

Seed stocks | *Report on the source of all seed stocks or other plant material used. If applicable, state the seed stock centre and catalogue number. If plant specimens were collected from the field, describe the collection location, date and sampling procedures.*

Novel plant genotypes | *Describe the methods by which all novel plant genotypes were produced. This includes those generated by transgenic approaches, gene editing, chemical/radiation-based mutagenesis and hybridization. For transgenic lines, describe the transformation method, the number of independent lines analyzed and the generation upon which experiments were performed. For gene-edited lines, describe the editor used, the endogenous sequence targeted for editing, the targeting guide RNA sequence (if applicable) and how the editor was applied.*

Authentication | *Describe any authentication procedures for each seed stock used or novel genotype generated. Describe any experiments used to assess the effect of a mutation and, where applicable, how potential secondary effects (e.g. second site T-DNA insertions, mosiacism, off-target gene editing) were examined.*

Magnetic resonance imaging

Experimental design

Design type | Measurements consisted of structural 3T MRI: T1, FLAIR, T2, diffusion, and susceptibility weighted sequences

Design specifications | Not applicable

Behavioral performance measures | Not applicable

Acquisition

Imaging type(s)	T1, FLAIR, T2, diffusion, and susceptibility weighted sequences
Field strength	3 T
Sequence & imaging parameters	Data used in this study:<br>T1-weighted 3D-MPRAGE (repetition time, TR = 2000 ms, echo time, TE = 2.74 ms, flip angle = 10°, thickness = 1 mm, matrix = 256 × 256)<br>FLAIR 3D (TR = 5000 ms, TE = 386 ms, inversion time, TI = 1800 ms, flip angle = 120°, thickness = 1 mm, matrix = 244 × 244)<br>T2-weighted turbo spin-echo (TSE) axial (TR = 4000 ms, TE = 82 ms, flip angle = 150°, thickness = 3 mm, matrix = 448 × 448)<br>Susceptibility-weighted imaging (SWI, TR = 27 ms, TE = 20 ms, flip angle = 15°, thickness = 2 mm, matrix = 256 × 232)<br><br>Data acquired as part of the protocol but not used in the present study:<br>Diffusion-weighted (TR = 3700 ms, TE = 108 ms, flip angle = 90°, thickness = 2.4 mm, matrix = 98 × 96, in a total of 113 gradient orientations with three diffusion-weightings: 800 s/mm2, 1500 s/mm2, and 2800 s/mm2)
Area of acquisition	Whole brain

Diffusion MRI ☐ Used ☒ Not used

Preprocessing

Preprocessing software	dcm2niix was used to convert data from DICOM to NIfTI<br>FreeSurfer segmentation was used to obtain likely location of the thalami.<br>ANTs and FSL registration tools were used to coregister all subjects.<br>Python code used in this study is freely available here https://github.com/vilsaira/AGU_SWI_analysis
Normalization	Data were normalized only for the purposes of atlas-based segmentation in Freesurfer and ANTs. Segmentations were used to extract thalami regions from the brain SWIs, which were not normalized as the interest was in their raw values.
Normalization template	Study specific templates drawn from the group were used.
Noise and artifact removal	Manual exclusion of subjects with large amount of motion was done. In the subsequent statistical analyses of MRI, robust analysis methods were used to minimize bias due to outliers.
Volume censoring	Volumes were censored based on expert review of the SWI images.

Statistical modeling & inference

Model type and settings	Permutation tests with FSL randomise were used to locate the iron accumulation regions in the thalami. Statistical analyses of the identified region were performed using the Python code available in Github.
Effect(s) tested	Test was performed to study correlation between SWI phase images and age of the subjects with linear and Gompertz models.

Specify type of analysis: ☐ Whole brain ☒ ROI-based ☐ Both

Anatomical location(s): ROI was based on fine-tuned thalamus segmentation.

Statistic type for inference<br>(See Eklund et al. 2016)	ROI-wise mean SWI phase signal was compared against subject ages using linear and Gompertz models.
Correction	Randomise was corrected for multiple-comparisons (FWE).

Models & analysis

n/a	Involved in the study
☒	☐ Functional and/or effective connectivity
☒	☐ Graph analysis
☐	☒ Multivariate modeling or predictive analysis

Multivariate modeling and predictive analysis	Iron accumulation in the thalami was modelled with linear and Gompertz models and used to predict the average outcome from the year 0 data. This group-average outcome was then used to compare subject-wise measurements from the years 1, 2, 3 and 4 data.
