# Supplementary material for: Pharmacological chaperone treatment with Cystadane in aspartylglucosaminuria: an open-label, phase Ib/II trial
Source: Nat Commun. 2026 Nov 11;17:11604. doi: 10.1038/s41467-026-78752-x (PMC13754814; doi:10.1038/s41467-026-78752-x)
Supplement: Supplementary file 4 [file 41467_2026_78752_MOESM5_ESM.pdf]

nature portfolio

Peer Review File

Pharmacological chaperone treatment with Cystadane in aspartylglucosaminuria: an open-label, phase Ib/II trial

Corresponding Author: Professor Ritva Tikkanen

This manuscript has been previously reviewed at another journal. This document only contains information relating to versions considered at Nature Communications.

This file contains all reviewer reports in order by version, followed by all author rebuttals in order by version.

Version 0:

Reviewer comments:

Reviewer #2

(Remarks to the Author)
The authors have declined to provide Zscores or age based normalization for the WISC. As such it is impossible to tell the degree of impairment at the start of the trial or during the treatment phase. In addition, given that this was an open label, there is no comparator group. The authors assert that there is historical data that shows continued regression but I do not find this to be convincing.

(Remarks on code availability)

Reviewer #3

(Remarks to the Author)
Thank you for responding to then comments of reviewer one

(Remarks on code availability)
NA

Reviewer #4

(Remarks to the Author)
The authors have addressed the remaining comments. They explained why they used raw WISC-IV scores, provided a mixed-model analysis with similar results, and added the healthy AGA reference range. The manuscript is suitable for acceptance.

(Remarks on code availability)

Open Access This Peer Review File is licensed under a Creative Commons Attribution 4.0 International License, which permits use, sharing, adaptation, distribution and reproduction in any medium or format, as long as you give appropriate credit to the original author(s) and the source, provide a link to the Creative Commons license, and indicate if changes were made.
In cases where reviewers are anonymous, credit should be given to 'Anonymous Referee' and the source.
The images or other third party material in this Peer Review File are included in the article's Creative Commons license, unless indicated otherwise in a credit line to the material. If material is not included in the article's Creative Commons license and your intended use is not permitted by statutory regulation or exceeds the permitted use, you will need to obtain permission directly from the copyright holder.
To view a copy of this license, visit https://creativecommons.org/licenses/by/4.0/

Responses to the remaining REVIEWERS' COMMENTS

Reviewer #2 (Remarks to the Author):

The authors have declined to provide Zscores or age based normalization for the WISC. As such it is impossible to tell the degree of impairment at the start of the trial or during the treatment phase. In addition, given that this was an open label, there is no comparator group. The authors assert that there is historical data that shows continued regression but I do not find this to be convincing.

We apologize for misunderstanding the request that was made by the Reviewer. We have now included the z scores for the baseline values in Table 1. The comparison was done with a control group of 6-year-old, healthy children. The z scores of our study participants are between -3 ≥ -1.8, showing that our study participants show a high degree of cognitive impairment at study begin. The study did not include a comparator group (i.e. placebo), as this would have been unethical, and the number of available patients was too low for splitting into two groups. AGU as a disease is known since almost 60 years, and the historical evidence for the cognitive impairment is substantial, as this is the main feature of AGU.

Reviewer #3 (Remarks to the Author):

Thank you for responding to then comments of reviewer one.

We thank the Reviewer for finding our manuscript acceptable.

Reviewer #3 (Remarks on code availability):

NA

We thank the Reviewer for finding our manuscript acceptable.

Reviewer #4 (Remarks to the Author):

The authors have addressed the remaining comments. They explained why they used raw WISC-IV scores, provided a mixed-model analysis with similar results, and added the healthy AGA reference range. The manuscript is suitable for acceptance.

We thank the Reviewer for finding our manuscript and responses to the comments of Reviewer #2 acceptable. We have added the mixed-model analysis in the Supplementary Figures, as requested by the Editor.
